# Holistic Practice in Traumatic Brain Injury Rehabilitation: Perspectives of Health Practitioners

**DOI:** 10.1371/journal.pone.0156826

**Published:** 2016-06-06

**Authors:** Courtney J. Wright, Heidi Zeeman, Valda Biezaitis

**Affiliations:** 1 School of Human Services and Social Work, Menzies Health Institute Queensland, Griffith University, Meadowbrook, QLD, Australia; 2 RECOVER Injury Research Centre, Griffith University, Meadowbrook, QLD, Australia; 3 ROBIN Team, Mater Children’s Hospital, South Brisbane, QLD, Australia; 4 Improving Treatment of Disease, Mater Research, South Brisbane, QLD, Australia; University of Perugia, ITALY

## Abstract

Given that the literature suggests there are various (and often contradictory) interpretations of holistic practice in brain injury rehabilitation and multiple complexities in its implementation (including complex setting, discipline, and client-base factors), this study aimed to examine the experiences of practitioners in their conceptualization and delivery of holistic practice in their respective settings. Nineteen health practitioners purposively sampled from an extensive Brain Injury Network in Queensland, Australia participated in individual interviews. A systematic text analysis process using Leximancer qualitative analysis program was undertaken, followed by manual thematic analysis to develop overarching themes. The findings from this study have identified several items for future inter-professional development that will not only benefit the practitioners working in brain injury rehabilitation settings, but the patients and their families as well.

## Introduction

Many thousands of people sustain a traumatic brain injury (TBI) in Australia every year, with a similar representation of diagnoses worldwide [1–6]. Defined as an injury to the brain arising from an external physical force (such as traffic accidents, falls and assaults, for example) [7], TBI results in heterogeneous outcome arising from factors such as the severity of impact, as well as the involvement of varied and often multiple areas of the brain [8]. Indeed, the burden of disability created from TBI not only creates significant economic and social costs to society [9,10], but can also cause overwhelming and lasting harm to individuals [1,11] with survivors experiencing increased long-term morbidity compared to the general population [12]. This situation makes traumatic brain injury and its rehabilitation a serious public health concern [2,13]. As a consequence of its heterogeneous nature, TBI leads to a varied range of physical, psychological and social difficulties [3,14] and as a result, requires a diverse range of rehabilitation efforts from a variety of health practitioners across different settings, over the recovery course.

### Rehabilitation in Traumatic Brain Injury

The overall goal of rehabilitation following TBI is to assist the person to achieve the highest degree of cognitive, functional and physical capacity to maximise an independent post-injury life [15,16,17]. According to Chua and colleagues [15], rehabilitation is broadly defined as, “a problem solving educational process aimed at reducing disability and handicap experienced as a result of disease or injury” (p. 33). Although there is some consensus regarding the overall definition of rehabilitation, there is no single, clearly defined theoretical basis for rehabilitation after brain injury [18, 19]. Further, the evidence-base in brain injury rehabilitation continues to emerge, allowing for innovative and diverse approaches to treatment and support. The consequence of this situation is some variation in rehabilitation therapies provided to individuals following their brain injury. Despite the lack of a single approach, there is general consensus among practitioners that rehabilitation in these settings ought to follow a holistic bio-psycho-social model [20].

### The Holistic Bio-Psycho-Social Model

Building on the work of both the medical model [21–25] and psycho-social model [24,26,27], researchers confirm the importance of a combined model of rehabilitation; one that moves away from the strictly medical approach and incorporates key psycho-social variables to provide a more holistic or ‘whole of person’ understanding [28,29]. This holistic model is otherwise referred to as the bio-psycho-social model of rehabilitation [21,27,28]. Building on the seminal work of Goldstein [30], Engel in 1977 [31] and Moos in 1979 [32] proposed a shift from understanding an individual’s disease or illness solely as a physiologic condition (i.e., medical model) to include an individual’s experience with disease or illness (i.e., psycho-social model) [33]. Put simply, the holistic approach can be defined as recognition of the dynamic relationship between the person and their environment [17,27,28,30,34]. It is not only the biomedical or social factors that are relevant, but rather the inclusion and *interrelationship* of all aspects of functioning for improved rehabilitation outcomes. The application of this best-practice interactive perspective of the bio-psycho-social model remains highly relevant to the practicing clinician [28,35] and reflects the key principles of the International Classification of Functioning, Disability and Health (ICF) proposed by the World Health Organisation [36].

### Implementing the Holistic Approach in Rehabilitation

According to researchers, clinicians and policy makers, providing holistic (that is, physical, psychological, social, emotional, and motivational) rehabilitation to injured individuals is a leading approach in brain injury rehabilitation and a determinant of good quality care [3,17,35,37,38,39]. Key strengths of the holistic framework are its adaptability to the needs of the individual person, its flexibility of application, and the creative approaches to healthcare that follow from its implementation [40]. Although it purports to be flexible in its design, the holistic approach does not offer a specific blueprint for health practitioners working with survivors of brain injury. It merely prompts the practitioner to *conceptualise* the patient’s health concerns in a more holistic manner by encouraging consideration of a diverse range of factors. Most of all, it requires practitioners to work in an inter-professional way in a complex health context, not only sharing discipline knowledge and resources but developing a shared view of the client’s needs and goals [17,41]. Thus, despite its significance in the literature, the implementation of holistic practice is inconsistent at best and not well understood [42], conceivably due to complex practitioner setting, discipline, and client-base factors.

#### Influence of rehabilitation services and settings

Rehabilitation settings specific to brain injury have emerged to address the unique needs of individuals with TBI [15]. These settings provide a complex continuum of rehabilitation services to support the individual in their recovery [15] and include the inpatient phase (comprising acute and sub-acute settings) as well as the community phase (comprising the tertiary community settings).

Different yet equally important strengths for both inpatient and community settings have been highlighted as each address different aspects of functioning depending on the person’s stage of recovery and availability of, and access to, resources [42]. While the delivery of services across the continuum aims to assist the person to achieve the maximum degree of return to their previous level of functioning [15,16], there is evidence to suggest that healthcare outcomes nevertheless vary across settings [43,44]. Indeed, researchers have suggested that different rehabilitation settings (and factors associated with each setting) can make a difference in the outcomes of individuals. For instance, Whyte and Hart [45] report that acute rehabilitation settings are associated with superior rehabilitation outcomes compared to community-based programs, although concede that, “We can only assume that greater comprehensiveness and intensity of rehabilitation services was responsible for enhanced outcomes as dimensions of treatment were not analysed” (p. 624). It is therefore possible that outcomes can be impacted by different settings, and factors associated with the amount, intensity, and comprehensiveness of rehabilitation delivery that each setting are able to provide, all influence the ability of practitioners to provide optimal (and potentially) holistic rehabilitation.

#### Influence of discipline specialisation

In addition to the diversity of settings impacting on the delivery of a holistic paradigm, practitioner specialisation within multidisciplinary rehabilitation likely influences the implementation of holistic practice by practitioners. The holistic or bio-psycho-social approach emphasizes the need for practitioners to view the person as a whole, particularly in the case of complex health issues such as brain injury [34,46]. This means incorporating the person’s unique physical, emotional, psychological, social and spiritual wellbeing into diagnosis and treatment plans to suit the person’s familiar home, vocational and social environments, regardless of practitioner-discipline orientation. It is evidenced by the literature however, that perceived functioning and health differs when viewed from either the medical or the allied health perspective, where traditional medical models contrast with social models of rehabilitation [47,48].

Different healthcare disciplines embody different philosophies [49]; ideologies that have been instilled into practitioners early in their professional career [50]. These practitioner perspectives naturally influence the interpretation of the holistic approach and its subsequent delivery. Although the biopsychosocial model (in conjunction with a phenomenological understanding of the patient’s experience [“lifeworld”] to more fully conceptualize the person’s issues [51,52]) features prominently in the agendas of current medical educational institutions [46], in practice, medically-oriented interventions led by medical practitioners generally continue to be targeted towards the disease process with limited consideration of broader psycho-social factors [53]. Further, the approach to medical training has largely focused on important medical outcomes rather than on lasting doctor-patient engagement, where a doctor’s immediate concern is to save the life of their patient rather than engaging and involving patients in their treatment and rehabilitation plans [49]. The doctor-patient relationship in acute care is understandably a pragmatic one, with patient self-management and holistic needs appropriately being the focus of post-acute rehabilitation as delivered by a different team of medical and allied health professions such as occupational therapy, social work and psychology [49]. It is in this transition from acute to post-acute care that holistic practice becomes most relevant.

From an allied health perspective, “patients’ functioning and health are associated with, but not merely a consequence of, a condition or disease” ([47], p. 934). That is, a person’s injury, disease, or illness state forms only part of the rehabilitation picture, rather than the whole of that picture. In keeping with the ICF model, important personal and environmental factors must also be considered in relation to the rehabilitation context for improved patient outcomes [28,54]. As suggested by Petrie [55], “each profession has a different ‘cognitive map’ and that, quite literally, two opposing ‘disciplinarians’ can look at the same thing and not see the same thing” (p. 35). Despite the primary diagnosis, patients may require different discipline specific therapy activities [45], however it is the way in which these diverse therapies come together to form a holistic program that is crucial. When working collaboratively, “individual team members undertake profession-specific roles, however as a team, they identify and analyse problems, define goals and assume joint responsibility for actions and interventions to accomplish those goals” ([49] p. 192). Medical, nursing and allied health practitioners must provide integrated care to individuals in an inter-professional context [41]. The literature clearly supports the need for expert discipline specific insights in brain injury rehabilitation, but also highlights the likelihood of different or contradictory interpretations of holistic practice and its subsequent delivery. Thus, the way in which practitioners combine their individual expert approach with a collaborative team effort to provide holistic rehabilitation within a single setting remains unclear. It is plausible that discipline specialisation and segmentation may actually get in the way of good (holistic) practice. Practitioner specialisation therefore, whether medicine (including nursing) or allied health oriented, can present a risk for practitioners intending to employ holistic practice.

There is also considerable variation in the terminology surrounding the holistic approach, where ‘bio-psycho-social model’ and ‘holistic care’ are used interchangeably with concepts such as the ‘social model of healthcare’ and ‘person (or ‘patient’; ‘client’; ‘consumer’; ‘individual’; or ‘family’)-centred care’ across much of the allied health literature [3,46,56,57]. While these concepts may be complimentary in theory, there are noteworthy differences between the bio-psycho-social model and the person-centred care paradigm, which evidently have distinct implications for practice. The bio-psycho-social approach is designed for use by practitioners to help them conceptualize patients’ health concerns and rehabilitation goals across all facets of a person’s life (i.e., physical, psychological, spiritual, emotional, social, vocational) for improved rehabilitation outcomes. In the person-centred approach, the focus is on early and ongoing engagement and participation of the patient and their family, and patient ownership and control over the rehabilitation process [3,57]. Both approaches operate in a complimentary fashion and reflect current clinical practice [48], however, when both are implemented fully, there is potential for uncertainty and ambiguity in rehabilitation roles and responsibilities.

Indeed, the increasing expectation of practitioners to adopt a person-centred approach to healthcare [57] results in ambiguity relating to the guiding principles of brain injury rehabilitation and its subsequent delivery [3]. As Wilson [58] explains, “Unlike medical treatment, which is something given to people to help them recover from injury or illness, or to make them feel better, rehabilitation, in order to be effective, must involve the disabled patient” (p. 487–488). From a person-centred approach, only the person can know their individual aspirations and desires for recovery [56,59]. Practitioners must therefore engage the person with impairment and their families as ‘expert-knowers’ to ensure the services provided directly meet the needs of the individual [56,60]. With no clearly defined theoretical basis for brain injury rehabilitation across the continuum, how practitioners adopt a holistic bio-psycho-social approach to brain injury rehabilitation while also incorporating person-centred methods to healthcare remains unclear.

#### Influence of adult-child client-base

Another factor influencing the delivery of holistic rehabilitation is the nature of the client-base, whether adult or child oriented. Researchers have found that the implementation of holistic practice varies between adult and child clients, where personal support networks and system coordination are more likely to support paediatric practitioners in providing holistic rehabilitation services due to the early and continual involvement of family, available friendship networks, and the support of the educational system [61,62]. This is in contrast to adult brain injury settings where clients may miss out on services all together [12], or it is difficult to re-engage adult social friendship networks and vocational services [63,64].

Overall, attending to the complex, holistic needs of individuals within the brain injury rehabilitation context remains difficult to achieve; particularly given the diversity of health professionals and multiple settings involved over time. Indeed, while there are useful studies exploring the views of health professionals in other injury and health issue populations (such as injurious falls in long-term care [65], pain in extremely low gestational age infants [66], and discussing sexual issues with patients [67]), a review of available literature has identified very few studies on how practitioners contemplate and subsequently implement holistic practice within brain injury rehabilitation settings, from the perspective of practitioners. Therefore, the aim of the present study was to more closely examine the day-to-day experiences of practitioners in their conceptualization and implementation of holistic practice in adult and child brain injury rehabilitation. Based on previous literature, it is anticipated that practitioners will describe an inclusive approach (i.e., multidisciplinary team approach that includes the person with TBI) to brain injury rehabilitation. It is also anticipated that clinicians will describe difficulties in implementing holistic practice depending on setting, discipline, and client-base factors. To address the research aim, the following research questions were proposed:

1. How do practitioners understand the established principles of brain injury rehabilitation?;
2. How do practitioners define holistic practice in brain injury rehabilitation?; and
3. How do practitioners implement holistic practice in brain injury rehabilitation?

## Methodology

### Research Design

This study draws on the phenomenological approach to qualitative research to understand the experience of providing holistic brain injury rehabilitation to injured individuals [68]. Alternative approaches that could have been used include grounded theory and ethnography. However, grounded theory is commonly used to generate a theory of human behaviour [68,69], and ethnography is often used to investigate human society and culture [68,70]. As the aim of the present study is to examine the experiences of practitioners in their conceptualization and implementation of holistic practice in brain injury rehabilitation, phenomenological principles were deemed the most appropriate to apply in this study.

### Participants

For this study, all health practitioners who were members of an extensive Brain Injury Network in South-East Queensland, Australia were invited to participate. From a potential sample pool of 80 network members at the time of the study, 19 members participated in individual interviews, representing a minimum response rate of 24% (note: given that members were asked to forward the invitation to their colleagues who were not necessarily formal members of the network, the maximum sample population is unknown. There was therefore insufficient information made available to the researchers to conduct a responders analysis to determine any potential bias between responders and non-responders). The age range of the participant sample (N = 19) was 23 to 57 years (*m* = 38.21 years of age; SD = 10.73 years of age) with the majority representing female respondents (94.7%; *n* = 18). Table 1 illustrates the cumulative sample of participants across their rehabilitation settings, disciplines and client-base.

**Table 1:** Crosstabulation of the Cumulative Sample of Practitioners Representative of their Settings, Disciplines and Client-base.

Setting^b^	Discipline^a^			Client-Base		
	Medical	Allied H.	*Total*	Adult	Paediatric	*Total*
	% (*n*)	% (*n*)	% (*n*)	% (*n*)	% (*n*)	% (*n*)
Inpatient Rehabilitation	25% (3)	75% (9)	100% (12)	50% (6)	50% (6)	100% (12)
% of Total	15.8%	47.4%	63.2%	31.6%	31.6%	63.2%
Community Rehabilitation	0% (0)	100% (7)	100% (7)	85.7% (6)	14.3% (1)	100% (7)
% of Total	0%	36.8%	36.8%	31.6%	5.3%	36.8%
**Total**	15.8% (3)	84.2% (16)	100% (19)	63.2% (12)	36.8% (7)	100% (19)
% of Total	15.8%	84.2%	100.0%	63.2%	36.8%	100.0%

^a^Discipline: Medical = 1 Medical Specialist and 2 Nurses. Allied Health = 1 Case Manager, 1 Music Therapist, 7 Occupational Therapists, 1 Physiotherapist, 3 Psychologists (including Neuropsychologists), 2 Social Workers, and 1 Speech and Language Therapist.

^b^Setting: Five practitioners provided both inpatient and outpatient rehabilitation to people with brain injury in their setting. Given that the rehabilitation occurred within the hospital, the views of these five practitioners have solely been classified as ‘inpatient’.

### Materials

Materials for the study included: (a) a participant information sheet and informed consent note; (b) a short, online pilot-tested (N = 5) demographic survey; (c) a standardized interview protocol; (d) a semi-structured pilot-tested (N = 5) interview schedule (see Appendix A); (e) a telephone; and (f) an audio-recorder. The interview schedule comprised ten individual items across three clusters reflecting the study research questions, namely: guiding principles of brain injury rehabilitation (Cluster 1), defining holistic rehabilitation in brain injury (Cluster 2), and implementing holistic rehabilitation in brain injury (Cluster 3) (see Table 2). The nature of the semi-structured design allowed the ten standardized questions to be asked of each participant, whilst remaining flexible to allow new questions, and subsequently new information, to be raised during the interview as a result of the responses given by the participant. All participants received the same materials. Rewards, including monetary incentives, were not used as an inducement to participate.

**Table 2:** Organisation of Clusters Based on the Study Research Questions.

Research Question	Cluster	Cluster Rationale	Interview Question
1	Cluster 1	To determine the guiding principles of brain injury rehabilitation	Q1, Q2
2	Cluster 2	To define and describe holistic rehabilitation in brain injury	Q3, Q4, Q5, Q6
3	Cluster 3	To examine implementation of holistic rehabilitation in brain injury	Q7, Q8, Q9, Q10

### Procedure

Ethical approval was sought and obtained from the Griffith University Human Research Ethics Committee (protocol HSV/10/11/HREC) and the Mater Children’s Hospital Human Research Ethics Committee (protocol 1852LNR). The participant information sheet and informed consent forms were emailed to all potential participants in the Network via third party recruitment procedures. Interested practitioners completed and returned the consent form (provided written consent) to the research team via email or fax. Consenting participants were then emailed the hyperlink to the demographic survey and provided the interview schedule. Supplying participants a copy of the interview schedule prior to the interview occurring provided each person the opportunity to prepare answers for their interview, if they chose to do so. All individual interviews were conducted by the first author over the telephone, digitally recorded, and transcribed verbatim (S1 File). It was acknowledged that practitioners were providing their time during off-peak hours and short breaks within a busy rehabilitation setting; therefore, interviews were kept to a maximum of 30 minutes per participant. On completion of the data analysis phase, all participants were offered the opportunity to provide feedback on the aggregated summary of results.

### Data Analysis

Qualitative analysis was conducted using a text analysis software package, Leximancer (Version 4, 2011), to identify a full list of emerging concepts. With adequate face validity, stability and reproducibility, the Leximancer program has valuable application for subjective data sources as it provides a more objective method of reviewing complex, but discrete blocks of text [71]. It purports to overcome reliance on subjective interpretations for large groups of data by producing a two-dimensional concept map that enables the researcher to develop grouped themes that characterize connected concepts [71]. Following the software-supported text analysis, a manual thematic analysis was completed to develop overarching themes.

In addition to using text analysis software, Fossey and colleagues [72] maintain that it is necessary to address issues relating to qualitative rigor in data analysis. For the current study, qualitative rigor was obtained through various methods, including auditability (i.e., the ability to trace the line of argument from raw data and replicate findings), investigator triangulation (i.e., comparing and cross-checking for consistency across data using two independent raters) [73,74], and member checking (i.e., consulting with participants to clarify meaning and interpretation) [75]. An aggregated summary of results was emailed to all 19 participants and two participants responded with no changes.

Reflexivity was also considered by the researcher who conducted the interviews (first author). Since the first author has no prior experience within the brain injury rehabilitation service system, she attempted to maintain reflexivity through in-depth discussion with participants who held differing views and by reviewing and reflecting on each participant’s transcript prior to subsequent interviews taking place. Finally, the qualitative data were subject to data preparation techniques (outlined below) to ensure an accurate and robust data set.

#### Data preparation

To begin the data preparation, interview data (N = 19) that had been transcribed verbatim were collated into ten separate Microsoft Word documents, reflecting the ten individual interview items asked of each participant.

Using the program’s default parameters, each document was individually and sequentially entered into Leximancer for an initial exploration of concepts and themes. A deliberate analysis strategy was then employed to set the parameters to remove ‘stop-words’ (i.e., functional words with low semantic content, such as ‘whereas’ and ‘guess’), and merge duplicate terms that did not provide additional meaning to related concepts (such as ‘rehab’ and ‘rehabilitation’). According to Smith and Humphreys [71], the presence of frequent stop-words and duplicate terms in the text can result in an overgeneralization of results. This preliminary analysis generated 38 themes and 264 sub-themes across the ten interview questions.

Following this preliminary text coding by Leximancer, further reduction of the sub-themes was undertaken to enable meaningful interpretation of the findings. To achieve this, the ten interview questions were subdivided according to the three cluster groupings reflective of the research aims and then subjected to secondary analysis in Leximancer for the purpose of within cluster thematic analysis. An average of eleven words were deemed stop-words or duplicate terms across the three cluster group analyses and subsequently excluded from the analysis. Following this step, several major themes across the three separate clusters that defined the way in which brain injury rehabilitation practitioners characterize and then implement holistic rehabilitation were identified. Seven overall themes were generated with a total of nineteen sub-themes.

## Results

### Cluster 1: Guiding Principles of Brain Injury Rehabilitation Themes

The results from Cluster 1 analyses showed two distinct themes of importance underlining how practitioners perceive the guiding principles of brain injury rehabilitation, namely: Specialised Rehabilitation and Goal-Directedness. Table 3 presents a summary of the results.

**Table 3:** Summary of Key Themes and Sub-themes for Cluster 1 Qualitative Analysis.

Cluster 1: Guiding Principles of Brain Injury Rehabilitation		
Key Themes	Specialised Rehabilitation	Goal-Directedness
Leximancer Connectivity^a^	(100%)	(70%)
Sub-themes	Multiple frameworks	Individual, needs based
	Person-oriented	Individual self-awareness
	Teamwork and collaboration	Discharge destination
	Participation	
	Family-oriented	

^a^The Leximancer Connectivity percentage indicates the relative importance of each theme (e.g., the higher the percentage, the more important the theme). The percentage is calculated using the connectedness of concepts within that theme.

For Cluster 1, the theme of Specialised Rehabilitation comprised a description of frameworks and practices tailored and targeted to brain injury rehabilitation. In relation to frameworks, it was evident that practitioners held multiple and competing frameworks in mind when they considered quality brain injury rehabilitation. For instance, practitioners identified the use of *“my [own] personal practice framework”* (ID: P3SW) in thinking about general rehabilitation, as well as international frameworks of practice (‘*the ICF’* [36], for example [*n* = 2]), discipline specific frameworks (*“my professional Australian Social Workers association”* [ID: P3SW]), client-base frameworks (paediatric developmental principles, for example [*n* = 3]) or more formal legal frameworks (the Mental Health Act, for instance [*n* = 1]). All practitioners were unclear as to which framework was of greatest importance, and believed they all contributed to some extent to their practice.

Practitioners also commented that any framework they held had to be person-oriented and tailored to brain injury depending on the individual capacity or circumstances of the client, *“It’s different injuries happening to different people in different contexts–you can’t have a one-size-fits-all approach”* (ID: P9CM), and *“there are often challenging behaviours that present themselves which may not be present in general rehab”* (ID: P4NUR). Paediatric practitioners highlighted this issue further by discussing the unique circumstances of children with a brain injury; *[It might be a little bit different for paediatrics in particular*, *due to developmental stages*.*] The injury happens to a developing brain and so any skills that may not have been acquired yet*, *the future development of those skills may be affected by the injury*. *So in terms of minimizing the impact of that injury damage*, *we don’t know what we’re aiming for in terms of the development of those skills in the future* (ID: P18PSY).

Teamwork, shared information and participation (of the person with brain injury as well as their families—especially when the injured person is a minor) were also important qualifiers to this theme. Although participants recognised that brain injury rehabilitation is best collaborative in its approach, this was dependent on a number of factors. These included how much the family were able to be involved in the rehabilitation (*n* = 8), how much the individual’s context was incorporated into the rehabilitation goals (*n* = 8), and how well individuals were encouraged to participate in their rehabilitation (*n* = 7). As highlighted by ID P1PSY, *“[A person’s context] is also very important*… *if you don’t look at their context then you really will never get anywhere with rehab”*. Further, participants emphasized system structures and their associated contexts (rehabilitation setting and functional approach used) as largely mediating these factors, thereby influencing the likelihood of collaborative principles being implemented into practice.

Goal-directed principles were also acknowledged by participants as necessary to the individual’s recovery. In particular, individualized goal setting (*n* = 4) using ‘client’-centred (adult sector) or ‘family’-centred (paediatric sector) (*n* = 8) and strength-based (*n* = 2) approaches that focus on re-engagement (*n* = 3) and identification of individual needs (*n* = 5) were identified as ideal. However, according to practitioners, the effectiveness of such approaches depended on the individual with the injury, as well as system structures. For example, the individual’s degree of self-awareness, insight, and ensuing capacity (i.e., *“[This can be challenging] if people demonstrate reduced awareness and insight”* [ID: P2OT]), ‘system time’ available to spend with each person, as well as the discharge destination of the individual are all illustrations of implementation factors that largely influence the efficacy of goal-driven principles.

### Cluster 2: Defining Holistic Rehabilitation in Brain Injury Themes

The Cluster 2 analyses revealed two distinct themes underlying practitioners’ definition and scope of holistic practice in brain injury rehabilitation: Person-in-Context and Patient-in-Care. Holistic practice in brain injury rehabilitation was defined by child and adult client-base practitioners as viewing the individual predominantly as a ‘Person-in-Context’ while incorporating ‘Patient-in-Care’ understanding. This potentially conflicting dualism essentially means to consider the current needs of the person and their social and psychological context, whilst actively managing their needs as a patient receiving healthcare and treatment. Practitioners described a number of influencing factors that subsequently determine how much the individual is viewed as a ‘Person-in-Context’ and/or a ‘Patient-in-Care’. Table 4 illustrates these themes and influencing factors.

**Table 4:** Summary of Key Themes and Sub-themes for Cluster 2 Qualitative Analysis.

Cluster 2: Defining Holistic Rehabilitation in Brain Injury			
Key Themes	Person-in-Context	Patient-in-Care	*Influencing Factors*
Leximancer Connectivity^a^	(100%)	(72%)	
	Healing	Functionality	*Environment access*
	Past, present and future	Treatment	*Service capacity*
	Control and coordination	Core activity capacity	*Physicality or visibility of injury* (versus invisibility of injury)
	Family support / context		

^a^The Leximancer Connectivity percentage indicates the relative importance of each theme (e.g., the higher the percentage, the more important the theme). The percentage is calculated using the connectedness of concepts within that theme.

To elaborate on the ‘Person-in-Context’ theme, *A holistic approach is seeing the person not just in the context of their brain injury*, *but seeing them as a whole*, *which is a person within the environment or context; a person within their social*, *family contexts*, *looking at what’s meaningful to them as a person* (ID: P5PSY).

Specifically, participants defined Person-in-Context as the interaction of the person with their environment. This included concepts such as healing (*n* = 8), *“rather than just the components …*. *of his disability*, *it would be looking at whether he’s [re]engaging in his occupation*, *how he’s going about those occupations*, *how he’s interacting with his environment and looking at his goals in relation to that”* (ID: P12OT); past, present, and future histories (*n* = 9), *“it’s about recognizing that the person has a life that existed prior to the brain injury and post brain injury”* (ID: P1PSY); and control and coordination of health services by the individual (*n* = 10), *“the participation has to be very much directed by the person themselves”* (ID: P9CM). While the importance of family support (and context) was reflected on by adult client-base practitioners, the support and active involvement of the family system was emphasized further by all paediatric practitioners in their definition of holistic practice: *I think [holistic practice and family-centred practice] go hand in hand*. *I don’t think that you can treat a child holistically in terms of rehab without considering them in their family context*. … *[However]*, *family-centred care is not necessarily the same as seeing a child in context either*. *You might be aware of the context but not necessarily modify your practice accordingly* (ID: P18PSY).

Also important in defining holistic practice but to a lesser extent was an acknowledgment from practitioners of the physical functionality, medical treatment and core activity capacity of the person with the injury (Patient-in-Care). Subsequently, the person is viewed as a whole where the injury is part of the package, rather than the injury being the entirety of that package, *“[the brain injury] is not the primary focus”* (ID: P5PSY).

Additionally, the scope of holistic practice in brain injury rehabilitation depends on a number of influencing factors that subsequently determine how much the individual is viewed as a ‘Person-in-Context’ and/or a ‘Patient-in-Care’. These factors include extent to which the practitioner has physical access to, or only background knowledge of, the person’s home environment (*n* = 9), service capacity (*n* = 8) and the physicality or visibility of the injury (*n* = 4). As ID: P14OT describes, *“It really depends on access–access to the client*, *access to their home situation*, *school*, *work environment and the different reasons why that access might not be provided”*. Thus, although participants agreed on how holistic practice in brain injury rehabilitation should be defined, it was also clear that a number of factors influence the likelihood of these definitions being realized in brain injury rehabilitation.

### Cluster 3: Implementing Holistic Rehabilitation in Brain Injury Themes

Three central themes emerged regarding the implementation of holistic practice in brain injury rehabilitation: Processes; System Supports; and Client Outcomes. Interestingly, as highlighted by participants, System Supports and Processes (assessment, relationship, and referral) greatly influenced Client Outcomes (refer to Table 5 for an overview of results).

**Table 5:** Summary of Key Themes and Sub-themes for Cluster 3 Qualitative Analysis.

Cluster 3: Implementing Holistic Rehabilitation in Brain Injury			
Key Themes	Processes	System Supports	Client Outcomes
Leximancer Connectivity^a^	(50%)	(30%)	(30%)
	Assessment processes	Funding and resources	Skill transfer
	Relationship processes	Appropriate frameworks	Referral outcomes
	Referral processes		Transition outcomes

^a^The Leximancer Connectivity percentage indicates the relative importance of each theme (e.g., the higher the percentage, the more important the theme). The percentage is calculated using the connectedness of concepts within that theme.

Various Processes incorporating assessment processes, relationship processes and referral processes were identified by participants as relevant to holistic practice implementation. In relation to assessment processes, practitioners reported that assessment methods needed to be comprehensive in their scope and facilitate mutual understanding between all relevant parties (including the injured person and their families) in order to inform and educate (*n* = 12). Also identified as important in the assessment process was the need for practitioners to think outside of their own specialist skill set (*n* = 4), and promote ‘whole of person’ practice through teamwork mechanisms that encourage the practitioner to endorse early and comprehensive assessment practices (*n* = 11), *“*… *no matter what our background*, *we all undertake the same holistic bio-psycho-social assessment process”* (ID: P9CM). According to practitioners, the success of assessment processes and subsequent outcomes was dependent on the early relationship and timely referral processes established.

Within relationship processes, rapport (*n* = 3), trust (*n* = 6), respect (*n* = 5), engagement (*n* = 15), family involvement (*n* = 7) and information sharing (*n* = 17) were highly valued by practitioners in the delivery of holistic care. It was reported by participants that the degree to which these relationship processes were positively established subsequently affected assessment processes (clarification of needs, feedback, collaboration, and teamwork) and referral outcomes. For instance, *Your relationship with the client and the willingness of the client to engage in a rehab program and their disclosure about particular things that are really important*… *[influences] whole of person practice*. *The client may not be willing to disclose that information [if a strong rapport is not developed]* (ID: P8OT).

Subsequently, client needs, necessary resources, and/or possible environmental facilitators and barriers may not be identified, leading to an approach that does not view the person within their context.

Participants also recognized that the implementation of holistic practice in brain injury rehabilitation is fundamentally driven by the system supports available in brain injury service delivery. In particular, rehabilitation was thought to be influenced by the capacity of the system to provide adequate funding (*n* = 7), access to resources (*n* = 12), as well as appropriate frameworks of practice (*n* = 10). According to ID: P5PSY, *“There’s not a lot of understanding of some of the more complex issues associated with brain injury”*, which in essence, can lead to fragmented service systems, lack of appropriate community-based resources, and frameworks that do not engage the person within their contexts. Although the majority of participants voiced concern over current system support structures, they felt that within the limits imposed by the system composition, their current processes underpinning holistic practice implementation as practitioners in brain injury rehabilitation was mostly adequate.

Individual outcomes as reported by participants related to how well the person’s learned skills during rehabilitation would transfer over to other environments (*n* = 3), the system capacity to refer to culturally appropriate services that are sensitive to the person’s needs (*n* = 9), and whether issues of transition from hospital to home or between children’s services and adult’s services were being adequately addressed (*n* = 4). As highlighted by ID: P6NUR, *It's very difficult for us to get that aftercare follow-up*… *[because] they don't fit into the mental health service quite often because they're not acutely unwell*, *the community mental health teams won't pick up most of our patients*, *even though we refer a lot of them to them*, *they don't see them as meeting their criteria*. *So they're just discharged and we hope for the best*, *you know?*

Referral processes, then, are at risk of not seeing the *“big picture”* (ID: P5PSY). That is, a person’s broader bio-psycho-social health needs may not be addressed once they have been referred out by the acute or subacute rehabilitation team.

Participants viewed individual outcomes as ultimately influenced by processes and system supports. In this way, implementation of holistic practice is not the sole responsibility of individual practitioners, but rather is a result of the systemic culture of the rehabilitation setting and its capacity to deliver. Implementation of holistic practice in brain injury rehabilitation was considered mostly adequate in terms of rehabilitation processes, reliant on the establishment of positive relationships between all stakeholders, and influenced greatly by structures and systems. As highlighted by ID P1PSY, *“*… *there’s nothing that brings it [brain injury rehabilitation] together over time*, *and there’s nothing that ensures that problems are picked up before they become a crisis”*.

## Discussion

The aim of this study was to qualitatively explore practitioner perceptions of the guiding principles, definition and scope, and implementation of holistic practice in the adult and child brain injury rehabilitation context. It was anticipated that most practitioners would describe an inclusive conceptualisation of holistic rehabilitation (based on well-established frameworks). The findings of this study supported this proposition, with participants confirming holistic practice was best defined by a collaborative bio-psycho-social approach (inclusive of patient- and family-centred philosophies) that was exceedingly tailored to brain injury rehabilitation. In this way, practitioners recognised the unique care and support needs of individuals with TBI. These needs included cognitive remediation, behaviour management, addressing self-awareness issues and facilitating adjustment over time. Indeed, teamwork, shared information, individualised goal setting, and participation of the person with brain injury (and their families) were also highlighted by practitioners as necessary to the individual’s recovery. These findings, unique to the perspective of brain injury rehabilitation practitioners, support previous literature investigating the perspective of patients, their families, and children’s school teachers [76,77], observation and intervention studies [78–80], and reviews [81–83].

It was also proposed in this study that practitioners would find implementation of holistic practice challenging for multiple reasons. The current study found that multiple challenges exist in implementing holistic practice ranging from patient factors to system factors. Despite the perceived benefits of what appeared to be an overarching approach to TBI rehabilitation (a bio-psycho-social approach), guiding principles and subsequent health professional practices appeared to be driven by multiple and competing discipline specific frameworks (for example, medical practitioners refer to Medical Board of Australia codes and guidelines; and social work practitioners refer to Social Work Australia guiding principles) and client-base frameworks (paediatric practitioners also refer to paediatric developmental principles). These results are consistent with those reported by Kuipers and colleagues [42] where the complexities of healthcare were acknowledged by practitioners working in this field, yet they struggled to manage these complexities in a larger system defined by discipline specific processes.

Participants also described other factors that contributed to holistic practice implementation success or failure, including available resources within individual rehabilitation settings, ‘system time’ provided to spend with each person, the capacity of the service system in supporting health professionals in referring clients to other services and settings, and how much the individual’s family were encouraged to participate in the rehabilitation program. The qualitative data indicated that despite a comprehensive approach (i.e., incorporating assessment, relationship, and referral management) to holistic rehabilitation, the translation of holistic theory or frameworks into practice and related outcomes largely depended on the processes undertaken by practitioners and were only as successful as the system supports that were available.

### Implications for Practice

Although system level factors (service capacity, for example) appear to be beyond the control of the practitioner, there are important implications from this study for health practitioners working within brain injury rehabilitation. For instance, findings from this study highlight opportunities for practitioners to maximise their holistic practice within their own setting by maintaining a strong focus on relationship and assessment processes. Specifically, rehabilitation practitioners have the capacity to ensure the following actions remain part of their everyday practice so as to facilitate positive outcomes for individuals with traumatic brain injury:

1. Build and maintain rapport, trust, and respect with patients and their families;
2. Purposefully engage the patient and their families in the process of defining (and redefining) their rehabilitation goals over the recovery course (i.e., reflects patient ownership and control over the rehabilitation process), while prompting broader conceptualization of particular rehabilitation goals across all facets of the person’s life (i.e., physical, psychological, spiritual, emotional, social, vocational). In this way, the *combination* of patient- (or family-) centred philosophy and the bio-psycho-social approach to brain injury rehabilitation may improve the person’s rehabilitation outcomes;
3. Practice inter-professional healthcare culture and communication;
4. Undertake timely and comprehensive assessment processes that facilitate mutual understanding between all relevant parties (including the injured person and their families) in order to inform and educate. Assessment processes should be collaborative in nature and respectful of discipline speciality; and
5. Provide appropriately detailed information to the person (and the person’s family) and to other team members, and maintain these open lines of communication across the rehabilitation trajectory.

Although these actions have been reported elsewhere [56,82], specific guidelines tailored to brain injury rehabilitation that detail *how* multidisciplinary practitioners can apply each action in practice is missing. The development of such guidelines tailored to brain injury rehabilitation would be especially useful for junior practitioners, or those new to the field, given that brain injury rehabilitation is vastly different to general rehabilitation settings [34].

### Implications for Policy Makers

Overall, the findings from this exploratory study revealed that practitioners in the current sample held a comprehensive understanding of holistic practice regardless of setting, client-base, and for the most part, discipline. The findings also confirmed that the implementation of holistic practice was dependent on environmental factors; most of which appear to be beyond the control of the individual practitioner and as mentioned above, rely on systemic level influences surrounding ‘access’: access to resources within the system, physical access to the person’s home, school, or vocational environment, and service capacity, in particular. The interrelationship of these environmental aspects appears important for policy makers to consider for optimal rehabilitation outcomes [84]. Indeed, the findings from this exploratory study suggest that the current brain injury rehabilitation system in South-East Queensland, Australia (and other service systems that are similar in composition) could further optimise the capacity of practitioners to provide best-practice rehabilitation. In the context of an aging population and increased brain injury survival rates, the provision of quality rehabilitation is imperative. Successful rehabilitation can significantly reduce the economic and social costs to both individuals and society, especially in the long-term.

Based on the findings of this exploratory study, the following items appear particularly important for future development in brain injury rehabilitation:

1. The communication of a unified framework of holistic practice, tailored to brain injury, to practitioners within an inter-professional context. Specifically, this framework should not represent competing models and professional standpoints, but instead combine important philosophical concepts of human rights with practical application of coordinated practice such as timely assessment and referral, and continuity of care. With a unified framework in place, increased collaboration and practice support across disciplines regardless of discipline background could enhance patient outcomes.
2. Further, patient-centred care was perceived by this sample of practitioners to be underlying one of the guiding principles of providing specialised rehabilitation, and is thought to be crucial to enhance patient outcome. This finding, together with the increasing expectation of practitioners to adopt a person-centred approach to healthcare [57], emphasises the importance of developing a unified framework of holistic practice that also provides guiding principles of patient-centred or family-centred care.
3. Using the abovementioned unified framework, practitioners would likely benefit from specific guidelines tailored to brain injury rehabilitation that detail *how* multidisciplinary practitioners can enhance their relationship and assessment processes.
4. A review of current referral processes and system supports available in brain injury service delivery in South-East Queensland, Australia could be undertaken to better determine transitional care needs and rehabilitation planning. Findings from the current study and previous research undertaken [85–89] confirm that the transition from hospital to home or between children’s services and adult’s services are of greatest importance. Participants in the current sample suggested their patients are at risk of ‘falling through the cracks’ because they often do not meet the eligibility criteria of community-based services. Given that brain injury is a ‘whole of person’ and lifelong injury, survivors must be adequately supported in the community.

These items are important to consider given that participants viewed client outcomes as ultimately influenced by processes and system supports.

### Limitations and Future Research Directions

The findings of the current study must be contextualised in relation to the study limitations. First, there was insufficient information made available to the researchers to conduct a responders analysis to determine any potential bias between responders and non-responders. Second, given the unequal representation of practitioner disciplines and settings, the generalizability of the current findings must be interpreted with caution. Future investigations could pursue greater sample representation (in scope: to include all disciplines involved with brain injury rehabilitation, as well as equivalence: obtaining equal numbers of practitioners across disciplines and settings). It is particularly noted that the current sample was limited in its representation of medical professionals, and that the involvement of medical practitioners were restricted to inpatient rehabilitation (i.e., no medical practitioners were involved in community-based rehabilitation compared to 100% allied health practitioners). This suggests that the clinical experience and contexts of practice were somewhat different between the medical and allied health participants. Females also represented a large proportion of the current sample, although this is not uncharacteristic of the allied health workforce which has high female representation. Future research ought to explore whether the findings from the current study may generalise to a broader range of aetiologies for diffuse encephalopathy (both of traumatic and non-traumatic origin). Further research also ought to investigate the efficacy of holistic brain injury rehabilitation by comparing the costs incurred in providing holistic rehabilitation with a rational assessment of validated outcomes (e.g., quality of life indices). Lastly, how practitioners might incorporate a phenomenological approach to holistic brain injury rehabilitation (in conjunction with the biopsychosocial model and person-centred care paradigm) is a noteworthy line of inquiry that could also be explored in future research studies.

## Conclusion

The present study extended our understanding of how brain injury rehabilitation practitioners conceptualise and subsequently implement holistic approaches in their everyday practice. Although untested, the study identified important key processes and influencing factors that might determine the eventual success of holistic practice. In line with previous research, the current study also highlighted the need for a systems-approach in achieving comprehensive rehabilitation in brain injury. Utilising a common framework could be the beginning of a changed system–multiple stakeholders (i.e., practitioners, patients, insurers, services, policy makers, families, and carers) ought to arrive at a shared understanding of holistic care facilitated by a consistent language.

Notwithstanding the reported limitations, and despite the potential usefulness and intuitive appeal of the holistic approach, practitioners have identified difficulties in implementing the holistic model in the brain injury rehabilitation of both children and adults. Indeed, researchers confirm that there is little guidance for how practitioners in Australia (and around the world) contemplate and then implement holistic theory into practice within the brain injury rehabilitation context [42]. The findings from this study have not only provided an insight into the challenges confronting practitioners when attempting to provide ‘whole of person’ rehabilitation for brain injured patients; but have also identified a list of items that could inform future development in brain injury rehabilitation. Addressing these items will likely benefit the practitioners working in this setting, the patients, their families, and the wider society as well.

## Appendix A: Semi-Structured Interview Schedule

Q1 Can you tell me what are the guiding principles of brain injury rehabilitation within your practice?
Q2 Are they different from general rehabilitation principles?
Q3 From your experience, how would you define holistic practice in brain injury rehabilitation? Please describe.
Q4 What do you think would be the opposite of ‘whole of person’ practice?
Q5 Does ‘whole of person’ practice look different for children and adults in brain injury rehabilitation?
Q6 How exactly might ‘whole of person’ practice change across practitioners and settings (acute; subacute; community)? Your answer can be as broad or specific as you like…
Q7 How do you achieve ‘whole of person’ practice in your discipline and setting?
Q8 How do you see other practitioners implementing ‘whole of person’ practice? Good examples / bad examples.
Q9 What external factors do you think might influence the real world delivery of ‘whole of person’ practice?
Q10 Can you provide an example of when you feel you didn’t or couldn’t achieve ‘whole of person’ practice within your discipline or setting, and why?

## Supporting Information

S1 File Interview Transcripts (Raw Data). (DOC)
