# Supplementary material for: Holistic Practice in Traumatic Brain Injury Rehabilitation: Perspectives of Health Practitioners
Source: PLoS One. 2016 Jun 6;11(6):e0156826. doi: 10.1371/journal.pone.0156826 (PMC4894634; doi:10.1371/journal.pone.0156826)
Supplement: S1 File — (DOC) [file pone.0156826.s001.doc]

**Transcription ID1:**

I: [Name withheld], thank you for agreeing to participate in this research on brain injury rehabilitation. I understand you have read the participant information sheet and have consented to complete a short interview. To remind you, our conversation will be tape recorded however any information you provide will remain confidential and will not be disclosed to anyone other than as part of a summary report which means results will be aggregated and your answers will remain non-identifiable. Your participation is also voluntary and you are free to withdraw from the project at any stage. Do you have any questions?

R: No.

I: Excellent. So to start with what do you think are the key principles of brain injury rehabilitation in general?

R: I think one of the main principles should be early intervention; so early as in while in hospital recovering. I think - these are my ideals aren't they?

I: Yes.

R: Yeah because I think a really ideal principle is family engagement and that doesn't happen. I think the other main principle should be something along the lines of continual engagement or something like that where there's a way of tracking people through their lives as they deal with new events because I think - what I've learned about brain injury is that each time a person hits a new challenge it can actually instigate a regression in terms of the brain injury that's quite significant to a level of disability that you wouldn't have predicted. So stress or change or something new - unfamiliar - can have a much more devastating effect on someone than someone with a spinal injury say.

I: Yeah definitely. So you've mentioned early intervention, family engagement and continual engagement.

R: Yeah I don't know what to call that really but some sort of continuity anyway.

I: Do you think continuity and these principles you've mentioned are put into practice within the sector at the moment?

R: No. No, not at all. I think there's some really good rehab in terms of content so the therapy, the actual delivery of services; things like that. Each individual element seems to be quite successful but there's nothing that brings it all together over time and there's nothing that ensures that problems are picked up before they become a crisis. There's nothing that engages the family. Very minimal anyway: you might get family counselling but that's different to engaging the family in the rehab process and actually enlisting them as part of the system and getting the whole family working together and so on.

I: Yeah definitely, it is very important. Can you tell me what are the guiding principles of brain injury rehabilitation within your own practice as a psychologist?

R: As a psychologist, my work is always guided by contextual based activity. So I work within the community, not in the hospital, so I had to find innovative ways of making things happen that actually matched with the community and the lifestyle and the family environment that that person was functioning in. So if I wanted to look at some cognitive issues that the person was dealing with my strategy was always to find something in the environment that would work already; something that was already happening, that the person could do more of to practice that activity, that skill or something in the environment that I could modify to change the outcome. Often it's more about modifying the environment than modifying the person.

I: Do you feel that these contextual based principles, from a psychologist's practice, differ to the general rehabilitation principles of early intervention, family engagement and continual engagement?

R: Actually the way - I probably should have listed context as another main principle because I really think that if you don't look at a - a person with brain injury if you don't look at their context then you really will never get anywhere with rehab. Even if you prescribe all sorts of things it won't happen unless you engage and enlist that context and support it to happen. So yeah I probably should have said that as a key principle.

I: So we'll add that one in there. Do you feel that these principles are easily implemented within your practice?

R: After a time yes, but I think it did actually take time to - for a start it takes more time to think about those things and sometimes the system doesn't allow you to do that. I was fairly fortunate in that I worked in a small service and we had a fair bit of freedom in terms of our funding body in what we could do. Otherwise if I had been - I suppose the other place was private practice: if I'd been in private practice and my interest had been in making a living from that private practice, because the bit of private practice I did was in addition to a salaried job. So I wasn't dependent on it but if I had been I suspect that I wouldn't have been able to spend as much time. So I think time is the biggest barrier - that we need to actually acknowledge that this is what's required - this is the baseline for people with brain injuries and change our thinking about how we allocate system time to brain injury rehab.

I: So the next few questions ask about your thoughts on what is holistic practice in brain injury rehabilitation. From your experience how would you define holistic practice in brain injury rehab?

R: I think it's definitely about all of those things that we just talked about, but all being done at once in a co-ordinated way. Also with input from a whole range of allied health practitioners, doctors, community people etc. All together so that they're actually learning from each other and benefitting from each other and that the person is part of that circle.

I: Would you say that person is the focus?

R: Yeah probably at the centre of that but somehow in control - more so than the focus. The focus definitely but in control of that process in some way: that they feel as though they have some capacity to say what they want to achieve from their rehabilitation and that whole team is looking at different aspects of their life but doing it together, and doing it at the direction of the person. It's kind of hard because I think in the system - in the rehab system - it actually breaks that up into components and it does that automatically whereas you and I just thinking about people's lives we can patch it all together; we can bring it together and say that I'm more than just my brain, my knee, whatever. I'm so many more things and it makes sense but once the system starts breaking those things down into manageable chunks you lose that holism. So I think it's more about how skilled the person is at managing the people in their life who are helping them. Then given that we're dealing with a population who probably aren't that skilled, perhaps even initially, pre-injury, but certainly not after injury, then we're dealing with the skill of the practitioners to enable that to happen and to communicate with each other and to not fragment a person. Once again I think it doesn't matter how much skill we all have, once we're put into a system that does it to us and has all the incentives to do that, then you can't pull it all together very well.

I: So do you feel that holism at a service level is there?

R: No, I don't think so. I think there's still very much little services - or big, some of them - who deal with certain things. I don't think that the answer is one service that deals with all the things because we've been there in history. We've been there where one service did everything for people and it led to problematic situations where people's lives were being treated in ways that didn't value them because the organisation had control of everything. So that wasn't so good. So I don't think it's one organisation doing everything but I do think there's got to be pathways: there's got to be clear connections and incentive for those parts of the pathway to connect. Then, not punishment, but consequences if they don't connect.

I: How would you define holistic practice at a setting level across acute - sub-acute - in tertiary settings?

R: I think probably a bit of the same. That it's recognised that it's a pathway over time that people are travelling and that they'll travel that pathway in their own way. So it's about recognising that the person has a life that existed prior to the brain injury and post brain injury and they don't suddenly become a patient and then at the end of that little piece of treatment they don't suddenly become a client and they're in the community and they've got [a service] for the case worker. Then at the end of that funding they don't suddenly become an isolate who doesn't have anything. So I think it's more about recognising the continuum of people's lives at each point from that - each of those levels.

I: What do you think would be the opposite of holistic practice?

R: Fragmented, specialist intervention - which is necessary - I'm not saying it's not necessary - but the whole system being made up of a lot of little bits that are fragmented, very specialised and not really interested in the person at the fundamental core.

I: Does holistic practice look different for children and adults in brain injury rehabilitation?

R: I think it has to just because there are different needs so with children I think it's much more obvious that a major need is to get the parent organised and understanding injury and brain injury. The education system is involved there and I think the implications of not being holistic for kids might be a little more contained than they are for adults. So for instance, a kid has a lot of other facilities and resources in their life like parents - hopefully - and extended family who are giving them that continuity and who - they have normal lives that go on around brain injury. But for an adult, I think, sometimes they can get lost in that whole process. Their family relationships - marriages - can break down, they may not have parents close to them anymore, the children - relationship with their children may become strained - and then they're left fairly much alone sometimes to manage things. So I think for them the - for adults the consequences might be greater.

I: Yeah. Thank you. So the next few questions are about how you might achieve holistic practice in your setting. How do you achieve holistic practice in your discipline and setting? Can you give me an example of how you'd go about putting it into practice using a holistic approach?

R: Well normally for me it starts with - it would start with getting to know the person so rather than focusing on test results and the injury - you have to do that, I know - but you need to actually learn about the person and who they are and what they live in, how they live, how they like things to be: that sort of stuff. So to me that's the basis of it is basing everything in who that person is. Then the next part of it would be actually working with them in a way that gives them choices and opportunities to tell you more about themselves. At the same time as sharing your expertise in this area, having seen so many more people with brain injuries, sharing some of that, but sharing it in a way that allows them to take it up rather than sharing it in a way that is imposing for them. So I think that's important.

Then I think it's important to ask and get permission - respectful treatment really I think is what it really comes down to - and just make sure that the person is comfortable and is feeling as though they can talk to you about the various challenges that they're facing without feeling threatened. Then I think it's about actually prompting people to think about all aspects of their life and how their rehabilitation can impact on that. So giving them the opportunity to talk about other things that they might not have come to you to talk about, but might actually be impacting on the problem. So, for instance, they might come for vocational rehabilitation say, but rather than focusing only on the job you might find that family situations are getting in the way of that or there's some personal adjustment work to be done. That all needs to be thought about all the time. So I think it's about having a very broad mind as you're looking at problems: being able to look at them from a range of different perspectives. That's probably about it. Follow up - just making sure that you've maintained some integrity to the whole process.

I: How do you see other practitioners implementing holistic practice?

R: I don't know that you see a lot of it which I think is sad. I think that it ends up being little tiny pockets of a very nice practice - I think out of all the services that exist at the moment I would say that [organisation name withheld] comes closest to holistic practice. Some of the work [name withheld] does. He has a really nice way of looking at a whole person, the bigger system, how it all fits together. So in that sense he's holistic about the work that he does with one person but he's also holistic about the work that he does in brain injury in general. He's able to think beyond one individual into how the whole system works a little bit better. I think that's very holistic practice when you can think like that.

I: That's how [organisation name withheld] is that little bit ahead compared to other settings?

R: I think so, yeah. Yeah I would think so.

I: Yeah. There are always challenges or difficulties in providing rehabilitation to people. What factors do you think might influence the real world delivery of holistic practice?

R: Yeah, mostly time. Discipline boundaries - so - and egos. I think the ego is probably the biggest barrier because to be a rehabilitation expert means to adopt that expert way of doing things but to actually work holistically means sometimes giving away some of that power. I think that's a bit of a challenge for some people. The discipline boundaries are really problematic because it sets people up to only do what they've been taught and often they're not taught to really understand what's coming - what could be dealt with through another discipline and to not appreciate what might happen if we all work together at those boundaries of the different disciplines. But then the main thing is time. As I said earlier time just - time and system constraints really prevent you from doing that.

I: Do you feel with the discipline boundaries that you touched on if, for example, practitioners start imposing on other disciplines thinking that they know how to do something which may be at a surface level?

R: Yeah I think one of the best things that could happen for holistic practice is that all the disciplines realise both the limitations of their discipline and the - where it meets other disciplines - and have a little bit more inter-disciplinary education to not necessarily avoid doing what another discipline does, but appreciate how that discipline might do it and adopt some of those strategies under the support of someone from that discipline. So it's a little more complicated than just having clear boundaries because we might transgress those boundaries regularly, particularly in rural areas and things like that. So I think in terms of transgressing boundaries it's really important that we do that in an informed way. We know where our limitations are but we also know a little bit about how different disciplines work and we get some supervision in terms of how we might adopt some of those strategies.

I: Do you have any ideas or suggestions as to how we might be able to do that?

R: I think it really starts back at the university. I think we spend a lot of time at the university pushing people into disciplines and in some cases completely ignoring other disciplines and I think that we need a lot more time teaching people at the university level how to work cross-disciplinary and how to appreciate what other disciplines are about. I know that some of the allied health disciplines each do a little bit of each other's subjects in first year. A lot of the disciplines do a little bit of psych in first year. I think it's more than that. I think it's more about when you get to third year, fourth year, projects that actually encourage us to collaborate across disciplines and show what we can achieve if we do that. The area where that need is probably greatest is across the medical and allied health area because the medical side of rehabilitation is often very separate from the rest of the allied health side. Then the physios sort of sit in that base a little bit but I just think it's about having collaborative skills and I don't think they teach us collaborative skill.

I: What are the challenges within your psychology discipline?

R: I think the main challenge for rehabilitation is not having enough psychology input so it's rare to have a psychology input and when we do have it, it's often fairly strictly about neuropsychology and I think there's an awful lot that clinical psychology can bring to the rehabilitation system. So I think other disciplines are not necessarily used to working with psychology so it's a little bit left out sometimes maybe. It's not considered as part of the team often. Only when there's mental health concerns; and in fact once you've hit mental health concerns then you're pretty serious. I think psychologists can actually contribute to a lot more of the general adjustment issues and family issues that people are experiencing. Often that's left to the social worker and the social worker has another particular job. That's more about organising the person's life and putting things in place so that they get support and things like that. Then there's often a case manager on top of that so it all gets really confusing and I think the biggest issue is just that. What is the role that each of us can play and how can we do that best?

I: So rehabilitation goals for patients may change across settings. Do you think this is the case?

R: Yeah I think people - the goals need to be the person's. At a certain point in the acute stage there are going to be goals that are jointly determined between the person and the medico, the physio, and they'll be much more therapeutically driven. So it'll be about getting certain body parts working again. That I think - it still has to be jointly driven so those goals still have to be something that the person is committed to as well. Then as the person moves through their life their goals are naturally going to change and I think that we need to be open to that. We need to be allowing that change to happen and actually checking in regularly rather than just assuming that everybody has the same goals and if we think it's good it's generally going to be good for them. Yeah I do think they change.

I: How might the focus of holistic practice change according to the clinician the patient is seeing?

R: In terms of discipline do you mean? A different clinician with a different discipline?

I: Yes. The focus of holistic practice though.

R: That's really hard because I don't necessarily think that every clinician has to do the same holistic approach. I think that it's how that clinician will work with the rest of the team. So it might be that as a psychologist I might have overall case management or whatever you want to call that. If I referred to a physio then I think it's about the way that that particular physio is brought into the mix, introduced to the person, clearly explain to the person what they're going to do and then how well they then work with that person and report back and it's all part of the discussion that involves the person. To me that would be how each individual discipline should work. They don't all have to do everything and all be focused on context and what have you. But someone has to. Someone has to sort all that out and be there for the person in their whole life rather than everyone just taking a little piece.

I: Just finally one last question. For a patient who has suffered a brain injury as a child to then move through the continuum as an adult, would holistic practice change or look different for them between the child injury services and the adult brain injury services?

R: Yeah I think it would. I think it would because we would have to be very careful that at some point we make that shift from you being the parent as the sounding board and checking in with parents all the time, to - and balancing that with checking in with the kids - but you'd have to make a shift to being driven by the young person. It'd be very difficult if you - to know when to make that transition and to know, particularly in cognitive rehab - cognitive disability - when the young person has that capacity to actually take over and manage their life themselves. I think that transition would be very, very difficult for the same service provider to manage. So if anything it feels like it might have to be even a special phase of its own: transitional type phase. That's - the purpose of that phase is to actually shift from a parent/family centred approach to one that really emphasises the individual as being in control of their own life.

I: Do you feel the sector currently accommodates for this transition?

R: I don't know a lot about that. I haven't done any paediatric brain injury. I've worked with young people and I'm not convinced that the sector really deals with them very well, no. I think that it just [unclear] them into the adult system and they're not always ready for that. So I can't really comment very much on that but I would say not.

I: Yeah, no that's okay. Is there anything you would like to add that we haven't talked about today?

R: No only that I really believe very strongly that whoever's operating in the brain injury rehab space needs to understand brain injury properly and I think it's such a subtle thing sometimes and has such a profound impact on people's lives and I'm not convinced that even the therapists who work in the area make - always fully appreciate that impact. I think you don't see that impact until you see people in their community, in their context and you spend a bit of time with them and you see that really subtle but incredibly profound impact that brain injury can have. So I would really argue that everyone who works in the sector right through to disability support workers have to be well trained in - or well predisposed to understand brain injury.

I: To provide optimum care?

R: Yeah. I don't think it can happen in the absence of that.

I: Yeah. Thank you very much. If there's nothing else you'd like to add I will stop recording now.

R: That's great.

**Transcription ID2:**

I: Thank you for agreeing to participate in this study examining brain injury rehabilitation. I understand you have read the participant information sheet and have consented to complete a short interview. To remind you, our conversation will be tape-recorded. However, any information you provide will remain confidential and will not be disclosed to anyone other than as part of a summary report, which means results will be aggregated and answers will remain non-identifiable. Your participation is also voluntary and you are free to withdraw from the project at any stage. Do you have any questions?

R: No.

I: Okay. To begin, can you tell me what are the guiding principles of brain injury rehabilitation within your practice as an OT?

R: I can, yep. I suppose one of the over-arching approaches or principles would be from the ICF, so really trying to focus and target therapy practice and interventions at activity and participation level, and then possibly breaking down to more impairment-based interventions.

I: Would you be able just to elaborate on that, using perhaps an example from a recent patient?

R: Yep, I can, definitely. I suppose to start with, at the moment in the brain injury rehab unit, our initial assessment forms and our discharge reports have just been updated and are being trialled and they're based on the ICF framework, so using consistent language with the ICF. I suppose how that would translate into practice - to start with an initial assessment; we'd be wanting to gain information from the patient and from their family about their participation in roles and activities prior to the brain injury. So getting a really clear understanding of what roles were really important to them, how they fulfilled those roles and, essentially, how they spent their time and engaged in occupations. Then, looking at their current occupational performance and their current activity and role participation and engagement. So looking at what impact the brain injury has had on those activities and that participation. So what activities are they currently able to participate in and what are their priorities for rehabilitation. I suppose that leads into the goal setting process, looking at what are the activities and roles that person would really like or is aiming to re-engage in. Obviously, within the brain injury population, there can be challenges to that process if people demonstrate reduced awareness and insight. So in that setting it might be the goal setting may be done in collaboration with the family and perhaps a therapist suggesting some goals given the likely discharge's destinations.

I: So, from what I'm hearing you say, is that the principles involve a lot of contextual factors and environmental factors that are surrounding the person and their injury and their recovery?

R: Yes.

I: Do you feel that these principles differ from the general rehabilitation principles of brain injury?

R: I don't think so. I think, at the end of the day, the purpose of rehabilitation is to facilitate or provide that person with opportunities to re-engage with as many things as possible. I suppose the ICF model I think makes us a little bit more aware about environment and not just the physical environment, but the cultural, the social and the broader community environment that would be in place prior to a person's brain injury and, also, the potential impact of the rehabilitation environment. I suppose - sorry, just trying to think of the right word. What are the barriers in terms of the environment? Because obviously, there are benefits to being in an inpatient rehabilitation facility in terms of access to therapy, but it also, in itself, creates barriers to engaging in activities.

I: The next few questions that I have ask about your thoughts on what is whole-of-person practice in brain injury rehab. From your experience, how would you define holism?

R: Now, I did read through this question before [laughs]. I suppose whole-of-person practice to me means when I am working with a client that I am working with them in the context of their person, their environment and, again, that environment being the social environment, the physical environment, so I'm not treating the person in isolation while they're in the ward here. I'm always, in my mind, thinking, well how is this relevant to the context that they're returning to? Just ensuring that exactly that, that it's not treating the person in isolation, that it's treating - working with a client in the context of their environment.

I: Some people may find it challenging to define whole-of-person practice. Do you have any suggestions as to why this might be?

R: I think I just found it difficult in parts to define.

[Laughter]

I think, potentially, it can depend on the background that you come from, in terms of the health professional background and the theoretical models that you work within. From an occupational therapy background, some of the models, like the Person-Environment-Occupation model, for us that's in itself, as a theoretical base, getting us to think beyond the person just sitting right in front of you, but about their environments and their supports. I think, also, because it's thinking whole-of-person, it could be overwhelming. There's a lot to think about.

I: Yes there is, isn't there? So what would you think would be the opposite of whole-of-person practice?

R: I think the opposite of whole-of-person could be impairment practice.

I: So the focus on the person's impairment or the injury that they have?

R: Probably even more specific than the injury. The actual impairment, so whether it's muscle weakness or whether it's visual disturbances.

I: So I'm just wondering if you could just elaborate a little bit more, just using perhaps an example.

R: Yes. I suppose for me the opposite to whole-of-person practice or holistic practice would be treating purely a muscle weakness, but not necessarily putting that weakness in the context of the activities that person wants to be able to do and the environment that person is going to do those activities.

I: Yes. Thank you for that. That highlights it well. Does whole-of-person practice look different for children and adults in brain injury rehabilitation?

R: Well, I haven't worked with children in brain injury rehabilitation. I would think that the approach wouldn't differ, that the background that you are treating the whole person including their environments, including their supports, including their communities, that philosophy would be the same with children and with adults. It may be that there might be more emphasis on particular things for the two groups. For example, social, like parents, might play an increased role working with children whereas for adults that might not be the same emphasis.

I: The next few questions ask about how you might achieve whole-of-person practice in your setting at [organisation name withheld]. You mentioned it at bit earlier with the forms that you have. Just wondering if you could elaborate. How do you achieve whole-of-person practice in your discipline and setting?

R: I suppose, within the discipline, is that we are using tools such as our initial assessment forms and our discharge reports that are prompters to therapists to cover all aspects of the person so that when they are completing their initial assessments they have a really good understanding of that person, of the activities that they participate in, of the roles that they engage in and what the environments outside of rehab look like for that person, so they really act as prompts and drive that process. Then, I think, depending - a number of different assessments might also help to feed into that. For example, the PRPP approach, which we're starting to use for more functional type assessments, is a very client-centred way of assessing of person's function and that would be one of the tools that we might use to guide our goal setting and intervention. Similarly, using a client-centred or family-centred goal setting approach, I think, again, assists to encompass what the person is wanting to achieve, what their family is hoping for them to achieve and, within those processes, you can build in information about the environment into the goal setting.

I: So it's all based on the assessment forms, the prompts that you mentioned? Just to remind you guys about those things?

R: Yeah. I think that, as okay as - the theoretical models already do that. As I mentioned before, we come from a background that when you think of a person, you're not approaching that person in isolation from their environment and their family and their roles. You're approaching them in that context. I suppose, if you think about models of intersecting circles where the person is right at the middle, but all of those other aspects are an integral part of them, of their lives and, subsequently, of their rehabilitation. The theoretical background that we come to a person with already has an approach where we're looking beyond that person just sitting physically in front of you to consider the broader picture for them and then, through the process, the initial assessment forms and things like that do act as prompts to ensure that we are conscious and aware of where that person sits within their environment.

I: How do you see other practitioners implementing whole-of-person practice?

R: Other practitioners being other OTs or…

I: Both, from other disciplines and other OTs as well.

R: Okay. I suppose, other OTs firstly to start with. It's similar to what I've just described. We're using tools that prompt us to do that. We're actively engaging family members and people's community or social supports in the rehabilitation process. We're, in the context of discharge planning, looking at home visits, looking at what supports are available to continue to support people once they get home. I think with other disciplines - in terms of specific models and approaches, I wouldn't be able to comment on those - but within a multidisciplinary team, I think all therapists or team members bring really valuable information to the team that can enable the team to have a whole-of-person approach.

I: You mentioned earlier that there are challenges and difficulties in providing rehabilitation. You touched on reduced awareness and insight as part of that. I'm just wondering what other external factors do you think might influence the real-world delivery of whole-of-person practice?

R: I think, firstly, the physical environment that we work within. It's obviously not a real-world environment. We're not providing rehabilitation in the context of that person's environment. We're not going to their home. We're not at their local shops. It's quite a different environment on the rehabilitation ward. It's a locked ward and there are routines that are unfamiliar to the patients who are in here and so it's a whole new - it's a different life. It's a different environment for them to get used to, so physically it's a different environment. In terms of the routines, it's a different environment. In terms of the supports and the people who are around, whilst there are quite lengthy visiting hours, there are periods of the day where the patients can be here, essentially, alone. I mean, obviously with the nursing staff and therapists, but away from their familiar social support networks. I suppose other barriers are more economic and resource driven. Looking at the therapy time as a resource and what's actually available to you, managing your caseload and knowing how much time you have available to interact or to provide interventions with each of your patients. Similarly, with economics, there's obviously a push depending on the setting, whether it's acute or rehabilitation. Ultimately, healthcare is expensive and we are frequently asked how long will this person be here, when will they have achieved their goals, when will they be safe to discharge home?

I: So there's somewhat a bit of, not a push, but an encouragement to get them through the process?

R: I suppose we need to be accountable for the time that they're here, so we need to be able to clearly outline the goals that we're working on and progress that the patient is making.

I: When you mentioned the physical environment earlier - I just wanted to make sure that I'm hearing you correctly - that translation from the ward environment to the real-world environment is a bit challenging at times?

R: It can be and there's been quite a bit of research done, especially for the population of people who have had a traumatic brain injury, looking at if, for example, you're in a rehabilitation setting, talking about strategies and providing education and practice of, for example, particular cognitive strategies to assist them to complete functional tasks, there's not necessarily the ability to generalise that across to different situations. I suppose for people that can be more challenging. Whilst we sit here and they might be able to complete a task in here, when it's being practiced for example, those skills will not necessarily transfer over to other environments. That can be challenging.

I: The reduced awareness and insights, could you just elaborate a little bit more on that?

R: Yeah. Quite commonly, the patients that we see - and, in the unit, we have a mixture of traumatic brain injuries and acquired brain injuries -, to generalise, often with people who have had a traumatic brain injury, reduced awareness and insight into the potential impact that the changes as a result of their brain injury might have on their function and on their return to their daily life. The way that we find that translates is that, if people don't necessarily identify that something has changed or that something is more difficult for them, they're less likely to engage in therapy to address those areas.

I: Definitely. Just to finalise the interview, rehabilitation goals for patients may change across settings. Firstly, do you think that this is the case?

R: Across settings being sort of acute hospital, rehabilitation and then community?

I: Yes, that's it.

R: I think that the patient's long-term goals may not necessarily change. On the acute ward, they may say that their goal is to return to work and to return to live independently and that may carry across from acute medical to an inpatient rehabilitation setting to a community-based rehabilitation setting. However, I suppose what may change and progress are the specific goals, perhaps the more activity breakdown goals. When it's broken down, it might be that their first goal is to be able to get up and shower independently, that might progress to being able to go to the shops, buy the ingredients and then make a meal, to then more complex community-access type goals, but I suppose all in the context of the patient's broader goal of being able to live independently.

I: My final question then is how exactly might whole-of-person practice change across settings?

R: I suppose if I think acute medical, then rehabilitation, then community as sort of the broad categories. Obviously, in an acute setting, there's a big focus on the medical issues and often therapists can be constrained by the medical conditions and restrictions that are on the patient and also by the physical environment. They may not have access to functional environments to provide interventions. The patient medically and physically may not be up to participating in more functional or more lengthy interventions. Sorry, can you just repeat the beginning of the question again for me?

I: Yeah, no worries. How exactly might whole-of-person practice change…

R: I suppose, to jump to the community, that's an opportunity to be more real-life focused and it really provides an opportunity where the interventions are being provided in that person's natural environment, so it's obviously more whole of person. It is in that environment. It's not simulating the environment, which might be the case in rehabilitation. It's actually providing the intervention and the input in the environment with, potentially, the supports that are usually there available to work through the tasks and potential difficulties the person's having.

I: So, I guess the subacute stage is kind of a mixture of the two? It's in the middle a little bit?

R: Yes, definitely.

I: Those are all the questions that I have for you. Is there anything else that you would like to add that we haven't talked about today?

R: No. I think we've covered it. Thank you.

**Transcription ID3:**

I: Thank you for agreeing to participate in this study examining brain injury rehabilitation. I understand you have read the participant information sheet and have consented to complete a short interview. To remind you, our conversation will be tape recorded. However, any information you provide will remain confidential and will not be disclosed to anyone other than as part of a summary report, which means results will be aggregated and your answers will remain non-identifiable. Your participation is also voluntary and you are free to withdraw from the project at any stage. Do you have any questions?

R: No.

I: All good?

R: All good.

I: Okay. To begin, can you tell me what are the guiding principles of brain injury rehabilitation within your practice as a social worker?

R: I've been working in brain injuries specifically as a social worker for about a month - because you didn't have your sheet, it said three months or three months or more. Mine's been less than that.

I: That's okay.

R: So basically what I'm doing at the moment is using my core social work skills. With that question, are you wanting to know about what my role is or what I do or…

I: Just what - what do you believe are the guiding principles of brain injury rehabilitation from a social worker's perspective?

R: Okay - as an outpatient or an inpatient?

I: Either or - or both.

R: [Pause] The guiding practical principles?

I: Yes.

R: So not my role, not my duty, but my actual principle of practice?

I: Yes, if you could.

R: That would be guided by the - so you're talking about personal practice frameworks, as in being the principles? I'm just - I guess I'm just wanting a little more…

I: A bit more clarification?

R: Yes, if that's okay?

I: Yes, that's fine. I guess - yes. The principles which - the practice framework principles, like you said - but however you interpret the question is fine. There's no right or wrong answers at all.

R: I guess when you're working with brain injury people, the way that I would work would be from my personal practice framework - which I guess is a social work tool - in terms of the theoretical models that we use to work with this particular group of people. So obviously when you're looking at brain injuries you're looking at the family system, because in some circumstances the individuals themselves don't yet have the capacity to process things because of the cognitive deficit. So you would actually work with the patient very, very differently to say, how you would work with the family.

So I guess some of the theories that I would be using to work with families would be cognitive behaviours therapy, solution focused problem solving, grief and loss. What other types of things would I be using? Off the top of my head - looking at the systems - so systems theory because obviously the systems involved in that person's life is very important. Looking at what their existing supports are, that type of thing. Then with the individual person that actually has the brain injury, that would be very, very different in terms of - a lot of that would be from a strength focus in terms of acknowledging, validating.

Depending on their cognitive capacity you could do some CBT and that type of stuff but it'd be more narrative therapy - so getting them to tell you their story. You might be able to do some grief and loss stuff with them again, depending on cognitively how they are going. So a lot of it would be supportive work rather than - or goal orientated work with them, because that's the concrete - because lot's of them are about that concrete thinking. Then I would be using my professional association which is the ASW - using their ethics to guide my practice as well. I can look up those, I can't think of those off the top of my head but I can look them up for you.

I: Yes.

R: Just bear with me.

[Interruption]

R: Okay. So of course you're looking at things like integrity - practicing with integrity - so that's obviously a very core principle.

I: Yes.

R: Human dignity and worth. Social justice. Service to humanity. Competence. Priority to clients. Having a cultural awareness - because obviously, especially with brain injuries as well, we do have people come in from a different cultural background - non-English speaking background - that also come in. That can be quite challenging work. So being really aware and being culturally appropriate. So they would probably be some…

I: Some of the guiding principles?

R: Yes, that I would practice by.

I: You've listed a lot. Thank you. Those principles that you've mentioned from a social work perspective, do you think they differ from the general rehabilitation principles at all?

R: Can you give me an example of some general rehabilitation principles?

I: I'm not sure.

R: Okay.

I: I guess I'm just thinking, because brain injury rehabilitation is a multidisciplinary approach, those principles that you've listed, do you feel that other disciplines may differ in their approach in general?

R: In general probably not, because you work as an interdisciplinary or multidisciplinary team. So as a team you work very collaboratively and closely together to ensure that you're all working towards the same goal. I'd be very surprised if the other professions didn't value someone's human dignity and worth and work towards social justice and integrity, looking at their professional competence, practice competence and the service that they were providing to that individual person or family. So I'd be very surprised if it was different. But I think the skill sets are different. I think that that's where [organisation name withheld] - that's where you have your mission and your value statements and stuff like that that the service has that states that that's the way the team works - which I guess in essence is a principle.

I: Yes. The next few questions I have ask about your thoughts on what is whole of person practice in brain injury rehabilitation. From your experience, how would you define holism?

R: Holism?

I: Yes.

R: As in the whole person or…

I: Yes. It's also known as whole of person practice.

R: Right. I've never heard of that in my whole entire life.

I: [Laughs]

R: I guess, again, I haven't been in brain injuries for very long, so it's really hard to answer some of these questions because - I guess again, at the end of the day, your primary client is the person with the brain injury. But then you've got other factors like your family systems, their support systems, their cognition, their - I have no idea how to answer that question.

I: No, that's okay. So before what you were saying is it's not just - from what I hear you're saying it's not about the individual person but their environments as well and other people that may be around them?

R: Yes. It's the systems. Like I said, I just have not worked in this area for very long. I've worked in other areas. So you're talking about spiritual stuff as well in terms of…

I: It could be. Yes - I guess there's not really a definition as such.

R: If you're looking at someone in terms of their whole from a social work perspective, you're looking at their spiritual, physical, emotional, psychological, sexual, financial - you're looking at everything. So you're working not just with the brain injury, you're working with everything around that person. The brain injury impacts those other areas of that person's life because they're no longer able to function or the way they function is very different to what they did before their injury or accident.

I: Yes. In that regard, what do you think might be the opposite of whole of person practice?

R: If you weren't focusing on someone's [unclear] or you weren't focusing on - you were, I guess, being a little more targeted or narrow in your approach. You only focused on one of those things and didn't acknowledge the other factors of what might impact that person's ability to be rehabilitated.

I: Sure.

R: So if you were just focusing on the cognitive stuff but then you didn't look at the financial stresses or impacts and that type of thing which would eventually create a crisis, I guess, if it wasn't addressed.

I: Yes. In your opinion, does whole of person practice look different for children and adults in brain injury rehabilitation?

R: I guess you would probably implement or practice it differently. But I would say it would be very similar.

I: Yes, no worries. I know - you've mentioned you haven't really been working in brain injury rehabilitation for very long, but I'm just wondering if you could elaborate just a little bit more on that? Your thinking behind why it might be different?

R: Because the issues would be similar but not the same because they're a child. You could still have the same types of financial pressures, you would still have the same grief and loss, you'd still have all those other factors around financial, emotional, social, psychological, financial, spiritual. You'd still have all those things that could potentially change for the family but you'd be doing a lot of work with the parents like you do anyway with adults. Again it depends on the level of the child's functioning. So you would still work from those same principles of practice. I don't think that those would change.

I: Yes. Just maybe the way they go about implementing might be different.

R: Yes, the way you go about implementing it might be different because a child's needs are different to an adult's needs. A child's awareness is different to an adult's awareness, depending on their level of cognitive function and things like that.

I: Yes, definitely. So the next few questions ask about how you might go about achieving whole of person practice in your setting. Firstly, how do you achieve whole of person practice as a social worker - because you work at [organisation name withheld] and the [organisation name withheld], is that right?

R: Yes, that's right.

I: So I'm just wondering how you achieve whole of person practice within those two settings?

R: Okay. I guess what I've been doing for the last month or so is basically - obviously social workers do what we call a psychosocial assessment. Within that psychosocial assessment we look at all of those factors. So we look at all of the family systems, the social support, the family situation, the family history. We look at their finances, their employment. We look at their accommodation. We look at their emotional coping skills and strategies. We look at their strengths. We look at a whole range of factors - we look at their adjustment to diagnosis. We then write that up in a report and then make an assessment which the interdisciplinary teams can then refer to if they need to regarding the person's psychosocial and psychological stuff that's going on.

So I do that for both inpatients and outpatients. From that initial assessment that I do with the family and with the person - in day hospital it's generally with the patients because obviously they've progressed with their rehabilitation and they're not in PTA. Sometimes they've got more of a cognitive capacity to participate in a one on one session. Sometimes I do joint sessions with a patient and then a family member. But generally when it's an inpatient I generally have to get a lot of my information from families because the person's in PTA. So once I get all of that I then work with the person around what some of their goals are - what are some of things that they would like to achieve?

It may be helping them identify possible stressors or pressures that they're currently experiencing and then help them put strategies in place to help manage that. So trying to decrease some of that stuff. Then I provide that feedback to the team. So what the team then has is an awareness of some of the psychosocial factors that may be impacting the person's ability to participate in their therapy sessions or maybe what some of the barriers are - them not being able to concentrate and that. Sometimes what we do - especially in day hospital more so than inpatient - is that if their psychosocial circumstances or their social circumstances are impacting their ability to participate in therapy, I'll then take that therapy session and spend some time with the person to unpack some of that stuff, put some strategies in place so that when they do come in for their therapy sessions they can concentrate on their speech, their OT or their physiotherapist.

I: It sounds like the environmental factors are really important in where your practice leads and what it involves?

R: Yes. If someone's especially socially isolated, that makes them much more high risk than say if they've got ongoing family support. So I guess my role is really quite crucial in making sure that it doesn't reach to a crisis situation where they're not coping and then something happens where they're placing themselves at harm - at risk of harm.

I: Yes. The next question I have might be a bit tricky but I'm just wondering if you could tell me how you see other practitioners implementing whole of person practice?

R: Okay. I guess from what I've done - the minimal stuff that I've done so far - is that the interdisciplinary team works very closely together. So what I'm seeing is that we'll sometimes do joint sessions, which I actually consider a whole of person practice, because we're not just thinking about our own specialist skills. We're thinking about how can we as a multidisciplinary team work together for the best interests of this particular patient to create opportunities for them to maximise their rehabilitation. So I'll sit in on sessions with the speech therapist or I might sit in on sessions with the OT and we might do some joint work together at that time.

I: Sure. You mentioned earlier that finding culturally appropriate rehabilitation guiding stuff may be a challenge or a difficulty in providing rehabilitation to people. I'm just wondering if - yes, what other external factors you think might influence the real world delivery of whole of person practice?

R: In terms of your cultural awareness and some of the barriers that you face, obviously some people that come through are from overseas on holidays so they don't necessarily speak English. Then all of a sudden to have a brain injury, be in a rehabilitation service - that's a scary thing to start with when English is your first language, let alone when English is your second language.

I: Definitely.

R: So I think that what we really need to be aware of if we're going to be practicing holistically or as a whole person is be mindful of what's culturally appropriate. So ensuring that we do things like booking in interpreters when we want to have a session to gain information. So I will always book an interpreter - asking them if they feel comfortable with an interpreter being booked because some people don't. Making sure that if I think that there's some psychological issues that I'll refer that out to [organisation name withheld] for them to do an assessment, because they will do it in a culturally appropriate way that is sensitive to their needs, rather than it being done from, say, [name withheld] or some other service provider. Again, always asking their permission to do that. Also, I think it's just being mindful of their culture and the fact that they're going to have some different challenges to some of the other people that might not come from an English speaking background.

I: Definitely. That's such a good point being raised. Are there some more external factors that you think might influence the real world delivery of whole of person practice?

R: I guess it's really contextual in terms of when you're looking at that cultural stuff. If you've got someone visiting from overseas, yes, the environment once they've been discharged, that's going to be very different in terms of their follow-up rehabilitation. Are they going to have their rehabilitation here? Are they going to have their rehabilitation back home? Is that offered back home - that type of thing. So I guess they're some of the differences. If they're already living within the community, then I think it's then about trying to find therapists - speech therapists - that actually speak their own language. So what we're actually starting to do is that when we're doing the assessments, instead of having to go through interpreters - which sometimes is fantastic - but you also can lose then things through that process. So if we could find professionals that could provide speech, physiotherapy in their native language, then that means that you're going to be practicing, I guess, holistically - or for the whole person.

I: Yes sure. Within their own culture and to try and find those services that's right for them.

R: That's right, yes.

I: What about Australian brain injury - those people who are from the mainstream culture I guess - what external factors do you think might influence the real world delivery of whole of person practice for them?

R: I guess again it's all those contextual factors. It depends on the person's social supports. It depends on how much they can finance and all the rest of it. But certainly once they get discharged and if they're needing quite a high level of care, lots of the barriers that we generally find is the resources in the community to assist and sustain their ongoing care needs. So a lot of it has to do with that community funding I would say, from a social work perspective, in terms of suitable accommodation - if they don't have family that are able to take care of them for various reasons or - that type of thing.

I: So it's a bit more of - yes, lack of access would you say?

R: Yes, I would say so - from my minimal experience here, yes.

I: Rehabilitation goals for patients may change across settings, through the acute, the subacute and community. Firstly, do you think this is the case?

R: Yes I do, because obviously as they go from acute to subacute back into the community, obviously their rehabilitation goals change because they're meeting their initial goals and they're re-evaluating and reviewing and setting new goals in terms of their rehabilitation and recovery. Each of those three stages is a different adjustment for the person. When we think about someone with a brain injury, it changes in some things that they cope very well with. Most people don't cope very well with change let alone if you have a brain injury. So those transitions from those three different areas can be quite an adjustment. So you're really going to have to redefine what your goals are because as things pop up along the way they can sometimes change the focus of the goals and what's happening for that person for them at any particular point in time.

I: Sure. My final question is how exactly might whole of person practice change across these settings?

R: Well, I guess that's really dependent upon what's happening for the person. So I guess I'm taking a very person centred approach when you're talking about holistic…

I: Practice.

R: …holism - whole of person practice.

I: [Laughs]

R: So - yes. I think it's very contextual and dependent. I think if you're using your basic principles of practice and you're working in an interdisciplinary framework, a lot of it is about reviewing and evaluating as you go along. I don't think that it's stagnant. It's a continually evolving process.

I: Sure - as to where the person is up to in their rehabilitation and, like you said, the goals may readjust as they're reaching them?

R: Yes, that's right. Yes.

I: Sure. That's all the questions that I have for you this afternoon. Is there anything else that you might want to add that we haven't talked about today.

R: No. But what I might suggest is to other people that are having the telephone conversation, just to mention to them in an email that you're going to be talking specifically about a particular theory. Did you have that in the…

I: I sent out a copy of the interview schedule. So you would have a copy of the questions there…

R: Did I read that?

I: …but that's…

R: I obviously didn't read that.

I: No, that's not a problem at all. [Laughs]

R: So that's my fault. I'm just having a look at my email. You did do that, so that's my fault. Sorry.

I: No, that's okay.

R: I should have done my research.

I: No, that's fine. The answers that you gave are very informative and I thank you so much for participating.

R: That's okay.

**Transcription ID4:**

I: Thank you for agreeing to participate in the study examining brain injury rehabilitation. I understand you have read the participant information sheet and have consented to complete a short interview. To remind you, our conversation will be tape recorded. However, any information you provide will remain confidential and will not be disclosed to anyone other than as part of a summary report, which means results will be aggregated and your answers will remain non-identifiable. Your participation is also voluntary and you are free to withdraw from the project at any stage. Do you have any questions?

R: No.

I: All right. So to begin, can you tell me what are the guiding principles of brain injury rehabilitation within your practice?

R: Oh, that's a very complicated question.

I: It's a bit of a tricky one. [Laughs]

R: Yes. [Pause] I'm not really sure of what context you're asking.

I: As a clinical nurse, what would be the guiding principles from a nursing perspective?

R: The same as anywhere else. So holistic patient care. Evidence based practice - all those sorts of principles.

I: Yes. What does evidence based practice mean to you - could you perhaps provide an example?

R: Okay, so basically it means that where there is a change in research for example and there's been shown to be a change in previous practice. For example, we often in the past had used rectal Diazepam for status epilepticus. There is now quite a large body of research to show that IM Midazolam but used buccally or intranasally works more effectively or is more easy to administer. So we are looking into trialling that in the future.

I: Sure - perfect example. So in your opinion, would the guiding principles of brain injury rehabilitation from a nursing perspective differ from the general rehabilitation principles?

R: I wouldn't think so. I think the only component would be the acquired brain injury. When there's - because there's a neurological deficit, then there are often challenging behaviours that come that present themselves which may not present in general rehab. For example, if somebody has a frontal lobe injury, then their personality changes will be evident in their rehab. Then that can be an issue for staff to be able to understand and how to manage those behaviours, for example.

I: Yes. Okay, so the next few questions ask about your thoughts on what is whole of person practice in brain injury rehabilitation. From your experience, how would you define holism?

R: Where you are looking at the entire person so that incorporates not only their physical situation but their mental situation, their spiritual situation and also their psychosocial situation - so how their interaction with the community and family and all those sorts of things needs to be taken into consideration.

I: Sure. Some people may actually find it challenging to define whole of person practice. What are your thoughts on why this might be the case?

R: I think because people - I know nursing for example in the past has been very task orientated. Sometimes it can be a little bit confused when we're talking about holistic person care because often nurses - and I'm talking very junior nursing - they're thinking about showers and breakfasts. They're looking at everything as a task. Whereas what we really try and do here is that we're looking at how that person reacts in their environment to certain situations. So for example, when you would normally - some nurses who perhaps haven't done work in rehab before might say to themselves well, it's much easier if I do the shower for them rather than thinking this is part of their rehab. If I can take a little bit longer then they're more likely to actually be able to relearn those skills again. Whereas just getting the shower done because I've got 20 others to do - I think that's why people get a little bit confused because they're not looking at the big picture, that this person really needs to rehabilitate so they can re-enter the community. They're not here just for me to do certain tasks to them for a short time while they're here.

I: Sure, definitely. So what do you think might be the opposite of whole of person practice and could you please provide an example of what that might look like?

R: Well, I think that probably takes me back a few years ago, probably 20 years ago when we used to line people up at 6:00 in the morning and you'd have one person taking off their clothes and they'd be pushed through into the shower. Then another person would do the shower and they'd be pushed through to another side where that person dries them off. Sort of really like a cattle call, where the actual person's experience wasn't really taken into consideration - it was just very task focused and all about the particular jobs that needed to be done. Whereas now for example we might think well, that person's always had an afternoon shower all their life so they might prefer to have an afternoon shower, rather than having to have a morning shower - just as a very simple example.

I: Yes - so accommodating to the person with the injury?

R: Yes, that's right. Also to their past life - whatever they've done up until this point in their life, they've chosen to live a certain way and we can try and incorporate that into their rehab programs.

I: Yes, definitely. The next few questions are about how you might achieve whole of person practice in your setting. So I'm just wondering how you might achieve whole of person practice as a nurse in your setting at [organisation name withheld]?

R: Something that we are looking at changing is the nursing skills mix. One thing we have is quite a number of assistants in nursing or personal carers. They've done a TAFE course so they're not actually governed by the registration board, they're not registered nurses. We want to change those particular roles through attrition over to endorsed enrolled nurses. So these are nurses that have done a Diploma in Nursing and they have an endorsement in medication. So the idea is that we would have a registered nurse and a couple of endorsed enrolled nurses working in say, a pod of six people, where they would give all the care for that particular patient - that group of patients.

So they would make sure that their ADLs were met and that they had adequate nutrition and that they had all their medications. But also looking at what sort of social interactions they might need and trying to be responsible in some way for the actual holistic care of those patients, rather than having [sides] and having the AINs doing all the showers and the registered nurses wandering around with the pill trolleys administering the medications. But actually looking at the individual with the team to focus on their care for that particular day.

I: Yes. Just to make sure that I've understood you correctly, just that the patient will more or less be interacting with the same nurse in their rehabilitation care with other team members?

R: That's correct, yes. So having a primary nurse who would look after the majority of their care - that's correct.

I: Excellent. So how do you see other practitioners implementing whole of person practice?

R: I think with the allied health for example, I know that particular therapies - say for example the occupational therapist wants to do an assessment of how they shower for example, they would work with the nursing staff in assessing what that patient's needs are and then trying to formulate a program so that improvements can be made. So they work together, it's not just the OT does their bit and the nurses do their bit. They try and have a team approach. This is evident in our case review. We have a weekly case review where everybody in the team is present. That's usually a clinical nurse and then all the allied health. We all give our report to our area of expertise and then try to come up with a plan of action, I suppose, to facilitate that patient's rehabilitation.

I: So really trying to collaborate within - with each other as a team in your meetings?

R: That's correct.

I: Yes, excellent. There are always challenges or difficulties in providing rehabilitation to people. I'm just wondering what external factors do you think might influence the real world delivery of whole of person practice?

R: I think budgetary constraints. That incorporates staffing levels, especially nursing staffing levels, but also availability of Allied Health staff. For example, we at this current time do not have a physiotherapist on staff. So that impacts greatly on the rehabilitation program of all of our clients. Probably another thing is the pressure from the bigger hospitals wanting to discharge their patients and hoping that they can come here but perhaps they don't really fit into our - they don't fit our criteria for example or - so there's a lot of misinformation about what we do and how we operate. So that can be an issue. Let me think - I guess the education level of nurses at times can be an issue. Because we can have such challenging behaviours, we require quite a specialised staff - so that can be a bit of an issue. If there's lots of leave and we have to backfill with casual staff who perhaps aren't familiar with the environment - yes, all those sorts of things.

I: I'm just curious about when you mentioned the patients that get discharged from the bigger hospitals and they may not fit in at [organisational name withheld]. I'm just wondering what may happen to those people?

R: If they don't come here?

I: Yes.

R: Well, that's a very good question. It depends. There is [organisation name withheld] and there's [organisation name withheld] on [location withheld]. They’re both slow stream rehabilitation facilities. But I think [organisation name withheld] does have some long stay beds as well. So there's a possibility that they go there. There are - there is a tendency for them to stay in hospital too long. The other problem is too that they might have to go to a nursing home which is unsuitable, especially if they're a young person. There are some young care beds but obviously not enough. So yes, there is…

I: It's not a good picture is it?

R: No, it's not a good picture at all. We only have - we've got 19 beds here. We can't - we're supposed to cope for all [location withheld]. It's ridiculous.

I: Yes, for 19 beds.

R: Exactly.

I: Yes. So you've mentioned a lot of external factors within the nursing discipline. I'm also wondering about external factors - contextual factors, for example, family and social networks - how that might influence the real world delivery of whole of person practice?

R: Okay. I think the discharge plan is a huge issue because there's just simply not enough places for people to go. That's one of the criteria that we accept people here - because it's a short-term slow stream rehab program - we really need for them to have a discharge plan in their head when they come here.

I: Something to work towards.

R: That's right because we can't - we don't want a bed block here either. So that can be an issue if say patients have plateaued for example and they don't make very good gains and really there is nowhere - their family isn't willing to take them home and look after them or they can't access funding from DSQ, then nursing home placement might be the only alternative. That is very difficult - it's very difficult as a practitioner to have to recommend let alone for a family to have to accept that that's what's going to happen to their loved one.

I: Definitely. It'd be very hard.

R: Yes, extremely difficult.

I: So further to your work as a nurse, I'm just wondering some challenges you may face specifically within your discipline as part of the rehabilitation team?

R: I think time is always an issue, trying to get everything done. Resources can be an issue, especially when budgets are pulled in and there isn't - for example, sometimes when staff are off sick, if there's no casual staff there's just nobody replaced. So we sometimes work down at times. Resources isn't usually an issue as in - it's [unclear] then that could be an issue. Sometimes access to - for the patients - to have equipment prescribed. For example, if they need to go through [unclear] to get a wheelchair for example, it could take quite a number of weeks or months before that chair arrives. So that can slow down the rehabilitation. Yes, that'd be about it.

I: So by resources you mean physical resources? For example, medication or a wheelchair…

R: Yes.

I: …and those kinds of things?

R: Yes.

I: Okay. So rehabilitation goals for patients may change across settings. Do you think this is the case?

R: [Pause] I think as - because when somebody has an acquired brain injury, in the initial phases of their injury, it's not - because the brain is so complex it's not known how much improvement they're going to make. So you might start a plan that this person, for example, can sit upright. Then as the goals are achieved then you would add on to those goals. You might have - I'm thinking of an example that we had of a fellow who was in - he came here. He couldn't do anything for himself, he was completely dependent on staff for everything. He wheeled himself out of here in his wheelchair and he could dress himself, he could feed himself. I'm sure the goals for him at the first stage of his rehab were extremely different to what they were at the end.

I: Isn't that amazing that he was able to do that?

R: Yes, it was fantastic. It was really great.

I: You guys must be so - must be feeling so fulfilled when you see something like that happen.

R: Definitely, yes. All the bad stuff - all the good stuff definitely outweighs - definitely.

I: Yes - when you see someone achieving their goals like that. That's great.

R: It does. It makes you realise when you look at somebody who perhaps comes in a [regency chair] and can't do anything for them, that you can't say well, this is how they are for the rest of their life. You have to think that there are possibilities for them to gain - to make gains. Yes.

I: Do you feel all practitioners might see it that way? We hope they do I guess.

R: I think that if you work here long enough you see it - perhaps not as a novice, people wouldn't think that way. But after they've been here for a while and they see the improvements that the patients make through different therapies - yes, definitely - it definitely changes the way you think about it.

I: Yes. So just to summarise across the settings, goals - you're saying goals for the acute, subacute and community phases will change for the patient?

R: Yes.

I: Just one final question, does whole of person practice look different for children and adults in brain injury rehabilitation?

R: I wouldn't think so because you would have to incorporate the same things for both children and adults. How that was delivered possibly would be different but no, I don't think there would be very many changes at all.

I: Okay. Is there anything else that you would like to add that we haven't talked about today?

R: I think there needs - we haven't really touched on the money side of it - the funding side of it. I think that's a really big issue. For example, somebody who comes in, has a third party claim, there's no problem for them to get equipment. I've even seen people who've been happy to take their loved one home because they know they're well supported with equipment and funding et cetera. I think that has a lot to do with how families approach the idea of taking one of their family members home to look after. If it's made a little bit easier with equipment and support, it's definitely more attractive than if they have to struggle and penny pinch and that becomes a big issue. But I think that whole insurance issue is a big, big factor that needs to be - I know that there is some work being done about that at the moment.

I: Yes, it makes it more realistic doesn't it?

R: Yes, definitely.

I: That they can actually continue to achieve their goals?

R: Yes.

I: That the rehabilitation doesn't stop when they discharge?

R: [unclear]

I: Yes, exactly.

R: That's probably another issue too, about community resources and how that impacts on further rehabilitation for clients when they leave here, if they have access to some other ongoing therapy.

I: You've actually just got me wondering - how you said that whole of person practice wouldn't be different or shouldn't be different for children and adults in brain injury rehabilitation, because I guess that it is - holistic practice needs to be there for both…

R: That's right.

I: …for both people. Do you feel that it's currently being implemented that way, the way the service is being set up at the moment?

R: Well, there aren't enough resources, in that there's not enough rehabilitation beds for young people - just because statistically I know how many beds we have here and the pressure that we have to accept people. Our actual age is 18 to 45, even though we do at times take outside of those age criteria. That's a very small window that - what happens to the people who are under 16 and what happens to the people who are over 45? They can easily get lost in the system. I think because of the way the health system is, it's very restrictive on who can access a lot of different things, not just rehab. So I think - I can't remember now where we were coming from. [Laughs]

I: [Laughs]

R: Drive myself around in circles.

I: You were saying that it should - holistic practice should be implemented for both age groups.

R: I think it would be, yes.

I: But whether it actually is is another…

R: I mean, here we strive to achieve that but we're probably just one of many, many places. I guess it depends on whether you talk about acute or the different stages of an injury for example. So that could change.

I: Yes. Is there anything else you'd like to add, with that one?

R: I don't think so.

I: That's everything?

R: Yes, I think so.

I: We'll stop recording now then.

**Transcription ID5:**

I: Thank you for agreeing to participate in this study examining brain injury rehabilitation. I understand you have read the participant information sheet and have consented to complete a short interview. To remind you, our conversation will be tape recorded however, any information you provide will remain confidential and will not be disclosed to anyone other than as part of a summary report, which means results will be aggregated and your answers will remain non-identifiable. Your participation is also voluntary and you are free to withdraw from the project at any stage. Do you have any questions?

R: No, that's fine.

I: Excellent. So to begin, can you please tell me what are the guiding principles of brain injury rehabilitation within your practice?

R: Within our practice here and within my practice, the guiding principles are client centred, participative or collaborative approach to rehabilitation or working with individuals with brain injury and their families. The approach uses essentially individualised goal setting and planning to assist with identification of needs, interests, goals and hopes for the future. The approach is, as I said, collaborative so the concept of that is to work with each individual on their goals and to facilitate the setting of goals and the achievement of goals. Another guiding principle I would say would be one which is an approach which encourages access to information, education to inform and to empower the person in regard to their understanding of their circumstances including their disability and in regard to the options available to them.

I: Sure. So you've listed quite a few. There's client centred approach, participatory approach, obviously collaborative and everything you've just said. Do you believe that these principles differ from the general rehabilitation principles?

R: I think it depends. If you can trust - it's probably community based versus more inpatient or outreach or outpatient rehabilitation. I think some of the principles are quite different. There's more of an expert driven or medically based generation of goals rather than participative or collaborative. There's more of a process in rehabilitation where the professional sets the goals and the patient or the client very much then receives the service that's offered as opposed to perhaps having more of an empowering role in determining the kind of service delivery and the pace of the service delivery and the process that's used. So that's quite different, I think. Community based rehabilitation principles are very different to inpatient or even outpatient day hospital rehabilitation. Certainly very, very different to the rehabilitation that's offered say in slow stream rehabilitation contexts such as [name withheld] you know, any of the other sort of more longer term stays or slow stream rehabilitation concepts.

I: Sure. So as a result of these principles differing across the continuum, I just want to make sure I'm understanding you correctly that the goals for patients may change across these settings as well?

R: Absolutely goals would change considerably both - but that's also a reflection of the stage of recovery and the kind of rehabilitation options that people need. But it does also reflect the process used in community based rehabilitation. So it is quite different and it will change for individuals with brain injury and for their families.

I: Sure. I'm just wondering if you could highlight that by possibly using a patient example?

R: Well, for example, you would have - I can give a current example. A young man, 23 years old who had a - a passenger in a significant motor vehicle accident, referred to our service after he had been in inpatient hospital treatment and rehabilitation at hospital and discharged home to his family's care with goals of returning to work, returning to driving, living independently again in his own home. Now, essentially he came from a medically based rehabilitation process where there's limited information and education, limited ability to self direct or guide goals. The rehabilitation is based on medical management of injuries, you know, medical treatment, therapy, nursing care that people dictate essentially that he should receive at that interval. Then when he moves to our service, it's all about well, let's look at what other goals that you want to set, what's important to you in your life, what things are going to make a difference to your life. Let's look ahead and see where you want to be in this time frame and let's look at the resources around how those goals might be achieved.

Let's look at some of the barriers and what we can do about those barriers. How can we work together on that? How can we, you know, move you more forward to where you want to be. So it's an individual or client centred approach as opposed to an approach which says well this is what the hospital service offers and this is what - this is best practice obviously. This is best practice in rehabilitation of hospital process. You need this kind of therapy. You need physio, you need speech pathology, you need this. We've determined that you need all those things. They're the things that we'll offer as opposed to what are the things that I really want. What are the things that are important to me. What are the things that make sense to me and that make me feel like I'm recovering and moving towards my - back to my life.

I: Sure. So I guess really putting it into the context of each individual person and moving forward?

R: Absolutely and that will be different. So his goals are different to say the next person I might work with who might not be quite so preoccupied with work or driving, but might be much more focused on some other area of their life. They want to put all their resources or most of them right now into that. Those other things may come later, but the timing in that, sort of the importance of their goals will change. So our service is very much about no preconception about the importance but let's look at what things are important to you.

I: To that person, definitely.

R: For that person at that time in that context that they're living in. Then we'll move along from that when other things come up that are equally important or equally need to be addressed, then you address them in a way that is responsive to the timing of that rather than, you know a constructed or a rigid process.

I: Sure, sure. Thank you. The next few questions ask about what are your thoughts on whole of person practice in brain injury rehabilitation. From your experience how would you define holism?

R: Holism. Well, I don't know what you mean by holism, but whole of person practice or holistic practice is typical of what I see in rehabilitation in brain injury in the community and certainly one I've used in psychology all of my professional life. So a holistic approach is seeing the person not just in the context of their brain injury, but seeing them as a whole, which is a person within the environment or context; a person within their social, family contexts, looking at what's meaningful to them as a person. The brain injury is obviously an important aspect of that, but it's not the only aspect of that. So it focuses on the person as an individual within their context or environment as opposed to just a disability or injury related concept.

I: Sure, so really taking into consideration those contextual factors?

R: Absolutely. I think essentially you're seeing beyond the injury. You're looking at the injury very centrally and it's of critical importance of course, but that's not the only thing you can consider. You're seeing the person as a whole, all of their strengths, all of their difficulties, all of the issues that come with them. The injury is then part of that rather than the whole of that or it's not the primary focus.

I: Sure. Some people might find it challenging to define holistic practice. I'm just wondering your thoughts on why this might be.

R: Well, I think it might depend on what perspective they come from in terms of why they would find that difficult. I think it does require you to be very flexible in the way that you practice and very responsive and to be adaptable. So instead of offering essentially one service, you'd have to shift your mindset to think outside of that and to think very differently. What you might want to offer may not be what the person may need. So essentially as a clinician, you have to be someone who's not concrete and inflexible. You have to be someone who can be responsive, who can change according to the circumstances. For some people that's just not possible. That's not what suits them best. That's not what they feel comfortable with. It's not something that they really can do. They can't think more abstractly. So some professionals just aren't suitable for this kind of work.

It also takes a lot of experience to be able to do it well because you need to be thinking ahead and thinking - and using lots of different clinical skills and resources. You have to use lots of approaches. You sometimes have to be open to trying something different and to make it work. Not everybody is able to do that or finds that a comfortable place to be because there's less certainty about that as opposed to someone comes in and I offer this particular kind of therapy every time I see - this is the kind of patient I see. This is the kind of therapy I offer. That's very predictable. It's very structured. It's very reassuring. People get a lot of satisfaction from knowing that they do one thing really well. If you take people outside of that, it's very hard for them to see and understand those concepts and think about what that means. It's more nebulous. It's more, as I said, it's more abstract at times.

I: Exactly. You know like we've sort of talked about with brain injury, it is individualized? So yes…

R: Absolutely, so each person that comes you have to be prepared to say well, what next?

I: Exactly. Definitely. Just going along those lines, what do you think might be the opposite of whole of person practice? Could you perhaps provide an example of what this might look like?

R: Well, let's see. The opposite of whole of person is - well I guess it's hard to probably conceptualise that there's one thing, but the opposite of that is someone in terms of rehabilitation who just offers a single point of service, I think. The example I gave before is probably a good one. You know, for example, the speech pathologist who just does [dysgeusia] work, work on swallowing or a physiotherapist for example who just does work on gait or a psychologist who only does adjustment counselling and does nothing else. Or there's probably a multiple number of examples or in fact a doctor who only treats a particular kind of tumour. You know, that's another very single example of someone who specialises to a high degree. So that would be the opposite of someone who focuses on a very singular area of the person as opposed to a holistic approach which is thinking about and considering all of those other aspects and being able to respond at least to those issues or to - what we do here is essentially is we don't provide all of the services, but we think about all of the services and then facilitate access to what's necessary for the person to respond to their needs.

I: Sure, definitely.

R: So it's not like as a clinician we're doing everything but it's thinking about all of those needs.

I: Taking into consideration in planning and implementation. Sure.

R: Absolutely.

I: This next question might be a bit tricky because I understand at [organisation name withheld] you work predominantly with adults. I'm just wondering whether you think holistic practice might or should look different with children and adults in brain injury rehabilitation.

R: Look I don't. I don't believe it should. I think it's actually more important with children because there's so many developmental and family issues and educational issues. So children are very much placed within a range of overlapping circles and contexts. Without the capacity to really look at all of those things I would think paediatric rehabilitation it would be even more difficult to effect change and to provide effective rehabilitation. So you can't for example provide services to children outside of the context of talking with their family. So it's very simple. At the same time that's very important in the adult rehabilitation construct as well. So I would think it would be the same, but probably of higher level of importance for children given their lack of ability perhaps too to articulate all of their needs and to work essentially on some of those goals. You'd have to work with other people as well.

I: Sure. The next few questions ask about how you might achieve holistic practice specifically in your setting. I'm just wondering how you achieve holistic practice in your discipline as a clinical neuropsychologist and setting at [organisation name withheld]?

R: How you achieve it? Well that's a complex question, isn't it? Well essentially the process that we use here at [organisation name withheld] facilitates that for all disciplines I think. That's that when we receive each referral - and the way that I work is that when I receive a referral I obtain as much information as I can so that I'm well informed and have an understanding of some of the issues that bring them to our service. So I would really seek to have a very good understanding of their brain injury and the treatment or rehabilitation they've had, the kinds of difficulties that they may be experiencing or the kinds of issues that may have already started to emerge in their early rehabilitation. The process that I would use then, that we all use here which is a process of engagement, information gathering and developing a relationship with the person and their family and using those things to facilitate the process of starting to think about, to identify, to do some of the work about thinking about goals and planning around that.

So the process essentially is one where you take time, you take the effort to listen to the individual, to reflect, to elicit more information from time to time, to start setting direction through using the tools that we have here. We use a number of tools with planning and goal setting. As a psychologist I would also use concepts from within my discipline to help the person think about what's important to them, to start identifying some of the psychological issues or adjustment issues, starting to identify some of the issues that are of importance to them as a result of their injury and the circumstances that they're currently living in. So the process is one of then continuing to work, essentially collaboratively, identifying issues, working on issues, checking back with the person, reflecting on change or reflecting on lack of change, identifying obstacles, identifying resources. Then essentially a process of working alongside the person for as long as that takes as they're achieving or working on those particular goals or starting to participate in life as they would have done before.

I: Before their injury.

R: Before their injury. So it's kind of a hard thing to describe really in practice. I mean, there's very practical things like goal setting tools and identity maps and life maps. There's things that we use around behaviour to allow the person to gain more insight and awareness and just to reflect and think about their behaviour and think about what they want to do and what they don't want to do. So there's a lot of very practical tools and very specific tools. The process itself however is very unpredictable and flexible.

I: Yes definitely. It sounds like the information gathering really lays the foundation…

R: It does. I think it does.

I: …for everything else, yes.

R: Well, if you don't get to know the person, you know, how can you really make assumptions and think about what their needs are and what resources are necessary and what the limitations might be or the barriers might be because you have to be - you can't be naïve about this. There are often longer term significant individual barriers, psychological barriers, family barriers, practical barriers like money or transportation or lack of education, all of those things. So the less informed you are…

I: The less able you are able to implement them yes.

R: Absolutely. You know, you are there because you - and if you don't inform yourself - and in the process you're also informing the person and their family too because you're getting them to think about what they're doing and think about why they're doing it. So if you're becoming aware of a barrier say, for example, an educational barrier or a psychological barrier, if they're aware of that then that's actually a greater resource for them to make a life change. If you're not aware of it how can they be aware of it?

I: Yes, that's exactly right.

R: You know, it kind of seems self evident. I know some clinicians in other context work, they say well I don't need the history, I just work in the moment. I don't believe you can do that, that's my personal belief.

I: Yes. Just going along those lines, I'm just wondering how you see other practitioners implementing a holistic practice.

R: Well I think everyone does it a little differently. I mean I think each clinician has particular strengths and has particular skills that they bring to that. Some people are much more concrete about it than others, so they work on things in a much more ordered way. Still with a holistic view but perhaps in a much more planned or organised way and an organised agenda that they might work through. As I said there are some clinicians who work - who don't feel they need to see the big picture. That suits them very well. I think the more effective people in rehabilitation have a more holistic approach and they get better outcomes and not only that, if you take a holistic approach, you should be achieving more independence in the longer term. So what I see holistic approaches do ideally if they're done well, is you teach the person to do the things that you're doing with them, so that ultimately they can be working on some of these issues independently of a professional. So you're teaching them to think about thinking.

I: Yes definitely. So being able to, as you say, work independently when they get home, back into their own environment, that context?

R: Well, most of the people we see are already at home but certainly once our service finishes, you want people to be still able to work on their goals.

I: Definitely.

R: Still able to prioritise and still able to say what might I need to do about this, how am I going to plan this or who do I need to call on to help me with that. If they can't do that on their own then they're always going to be dependent on a service, aren't they?

I: Definitely.

R: Which is not good I don't think.

I: No, not at all.

R: So you should be in the way that you work and I guess this comes back to how you do it, which is one of the previous questions, is you should be almost talking aloud with people about what we're doing. We're doing this because, we're talking about this because, so that they can see the process that you're using with them. It's not a hidden or mysterious thing.

I: So they better understand and are able to, like you say, collaboratively work together? If they're uninformed they won't be able to do that.

R: That's right.

I: Exactly. There are always challenges or difficulties in providing rehabilitation to people. I'm just wondering what external factors do you think might influence the real world delivery of holistic practice?

R: That's a good question. Well a key issue is lack of appropriate community based resources for people particularly with ABI. There's lots of resources for people with mental health difficulties, intellectual disability and other disability. Lack of funding. Lack of understanding of ABI has historically always been an issue and will always continue to be an issue, just in terms of individual needs. Practical barriers I think are around essentially funding and service provision. There aren't sufficient services available to people for that ongoing support. So lifestyle support work, counselling, therapy top ups, accommodation that's suitable for people that's affordable, you know, that offers a quality of life so that people can be independent and can participate. Lack of opportunity for supported employment or assisted employment that gives people a purpose and a sense of meaning. Lack of opportunity to contribute in a way that's meaningful for people who can't work.

So I think they're things that I see is that, you know, you can only link people if the services are there and the support is there. I think we still haven't addressed and we still don't have enough meaningful services that are available to people.

I: Sure. I'm just wondering what are some challenges within your discipline that you experience?

R: Well, let's see. There's not a lot of clinical neuropsychology in the community. Psychology generally I think - challenges. Well there's a lack of services, there's a lack of appropriate services that are accessible for people. I think particularly in the discipline there's not a lot of understanding of some of the more complex issues associated with brain injury. So particularly the behavioural and personality changes and how they impact on adjustment and mood and social relationships. So I think there's certainly a lack of understanding of those subtle changes. I think when I see people who undergo cognitive assessments I see a very narrow view of the individual. They don't have a holistic view of the person in their context and so they often don't have a good understanding through those assessments of the difficulties that people are encountering or the needs that they have or the needs that their family or carers may have.

I: So I guess really including the environment and contextual features from the very beginning?

R: Absolutely, yes. You know, sometimes you see these assessments and they say oh the person is doing really well because they're doing - you know getting scores on this test that are within the average range. They don't see in the everyday life, you know living at home with their family, that they've got enormous behavioural difficulties or they've got loneliness and isolation because they don't have any friends, they don't have anything to do in their day, they don't have any purpose, they don't have any sense of participation. They don't understand the everyday struggles that people presenting really well in an office for a couple of hours, that that doesn't reflect their everyday life.

I: It sounds like the assessments implications are twofold. One, just not giving a score per se to a person and putting the assessments in real life situations?

R: Yes. Again it's probably that compartmentalisation of the person rather than seeing the bigger picture. I think the challenge in psychology is that they think well look, I've done this really great assessment but it's such a small part of the person.

I: Part of the bigger picture, yes.

R: Absolutely. They don't see that it's such a small part either which I think is unfortunate. There's lack of understanding of that.

I: My final question. We spoke a little bit earlier about how goals may change across settings. I'm just wondering how exactly might holistic practice change across settings?

R: How it might change across settings. Well I think that's the reality. We're working with clients who are going to be working with a range of other people in different settings who don't have the same approach that we do. I think educating clients about that is actually quite helpful and educating their families about that. What we seek to do is when we work with other service providers who have different approaches is again to try and collaborate with them and to inform and educate and to assist them in perhaps seeing a little bit outside the square. We often have to do that with funding services to say well look, there's different ways that you could look at this or do this. I've done that for example with - recently with a client living in nursing care where you go and do some training and you say well look, you could do it this way but here's some other ideas that you could think about. So the approach that we have is and the challenge essentially is to challenge some of that thinking and to encourage and support and work with people so that perhaps they can offer a much more holistic service or a more responsive service. Or at least kind of occasionally think outside the square and say oh yes, we could do this or we - you know, that would actually be quite possible for us to do that.

So that's actually really important I think and a very rewarding aspect of what we do here is working with other services across the spectrum. The reality is they're - everybody's got different approaches depending on their eligibility, their funding, the staff they have at the time, the resources they have at the time. What our role is in working with our clients is to get the very best we can out of those services. They're not going to change essentially to a great degree, but there's no reason that you can't work towards systemic change and changes in attitudes and beliefs and willingness. You know, it's very exciting when you work with someone who goes oh my goodness, I never thought I could do that. That's really exciting - and I will try that and it works and that individual then sees that there's actually more than one way to work with somebody.

I: You mentioned earlier that in the acute setting the patients are less participative because it is more medically based. As practitioners implement holistic practice in that setting, I'm just wondering if you could provide an example as to how it might differ to you guys as a community setting?

R: Well I mean if they did taka a holistic approach say in the acute hospital setting, you mean? How would it differ?

I: Yes.

R: I think it would still be very much driven by the therapy or medically based goals. There's no reason why hospital services or a more inpatient service couldn't strive towards being more holistic. Certainly it just takes a language around that. It takes a willingness around that to say well what's important for this person. I think there's a cascade and an avalanche essentially of force essentially that keeps things essentially tied to that approach. I'm not sure. I think that's a very difficult question to answer easily actually.

I: It is a bit isn't it? It is. [inaudible]

R: I mean I think the only way they could do it would be to have a language around holistic approaches and more individual centred or client centred work or patient centred work to have an approach that facilitates the discussion of those perspectives. Certainly they try to do that with things like case conferences. Essentially having been in that context myself as a clinician and working with other people, even when you have that, each of the therapists are still concerned about their work. You know, they're still concerned about their intervention or their contact. It's not that they're not interested in those other things. They are. It's essentially driven by the need to get that work done within a timeframe. The timeframe imperative in inpatient settings and day hospital settings, that's a constraint that they work within that I think, you know, you can't not take into account and understand, is that they have huge numbers to push through. They have limited time and limited resources.

In the community we have a much more flexible approach. We can work with people for years if that's what it takes. Sometimes you have to. Goals don't get achieved quickly in the community. They have an imperative say in the hospital to treat the person, get the maximum recovery in the minimum time, move them along and discharge. They have an urgency about that. So that drives some of the work. So you just have to understand that I guess. It's not unreasonable, it's just different.

I: Yes, definitely.

R: Of course if you think about it too, if you've got a medical problem, you want your doctor to be absolutely focused 100 per cent on solving that problem. You know, you want them to be not distracted by those other things and to be as a jack of all trades. You want them to know absolutely with every certainty the specialty that's necessary to get you the best treatment and outcome. This is a different kind of specialty once it comes to that in the community.

I: Yes, it's very interesting, isn't it? That's all the questions that I have for you this morning. I'm just wondering if there's anything that you would like to add that we haven't talked about today?

R: No not at all.

I: We covered a lot.

R: That's covered it all thanks [name withheld].

I: No worries. I'll stop recording.

**Transcription ID6:**

I: Thank you for agreeing to participate in this study examining brain injury rehabilitation. I understand you have read the participant information sheet and have consented to complete a short interview. To remind you, our conversation will be tape recorded. However, any information you provide will remain confidential and will not be disclosed to anyone other than as part of a summary report, which means results will be aggregated and your answers will remain non-identifiable. Your participation is also voluntary and you are free to withdraw from the project at any stage. Do you have any questions?

R: No.

I: All good to go.

R: That's great.

I: Firstly, can you tell me what are the guiding principles of brain injury rehabilitation within your practice?

R: Okay, I've just written down a couple of notes here regarding - it needs to be an individual rehabilitation program for each consumer. I felt that the introduction of a routine is good for ABI rehabilitation. Familiar staff - the environment needs to be positive, heavy patient focused - not ward or staff focused, which sometimes rehabilitation can be. The environment also needs to be calm, low stimulus and care needs to be delivered in a non-judgemental, respectful way. I also work under the *Mental Health Act* so I guess we're always trying to promote optimum quality of life for the person. So they may not reach so-called normal or what they were previous to their acquired brain injury but they need to achieve - or want to be working towards achieving optimal quality of life. Something that can be sustained once they're back out into the community.

I: Yes, definitely.

R: I feel that the consumer should be involved in all the decisions regarding the treatment and care. We need to encourage participation by them and their carers and delivered in a least restrictive environment, which is something that comes from the *Mental Health Act*. I guess that's about all.

I: I'm just curious about environment and what you mean by that. If you could elaborate using an example, that would be great.

R: It's the whole - it's more like a therapeutic milieu. It's the whole atmosphere of the unit needs to be positive, the staff needs to be empathetic rather than sympathetic - not trying to do things for them all the time. Encouraging the individual to achieve their optimal function.

I: Sure.

R: But it's the whole feeling in the unit. I think it needs to be calm, relaxed and happy and routine. I think routine - people with an acquired brain injury function well in a routine - daily routine. Because they've forgotten so many things and - it just gives them a bit of - a sense of security and structure to their day. So if they know that breakfast is going to be every morning at 8:00 that's one less thing that they have to worry about. They can concentrate perhaps on their walking or some other aspect of their rehab without worrying about just those everyday routine things.

I: Sure, yes. I see what you mean.

R: Good. [Laughs]

I: [Laughs] So I'm just wondering whether the general rehabilitation principles of brain injury differ in some way to the principles of your practice within a nursing perspective?

R: I don't think so. I think the nursing principles are always the same in any rehabilitation approach. It's always about assessment, planning, implementation and evaluation and looking at the patient as a whole. So their biological, psychological, social and spiritual needs.

I: Yes, sure. So the next few questions I have ask about your thoughts on what you think is whole of person practice in brain injury rehabilitation. From your experience, how would you define holism in brain injury rehab?

R: Probably what I just said. It needs to look at the patient as an entire person. You need to look at their biological, biopsychological, social and spiritual needs and prioritise them with the patient - including them. Involving them in as many decisions as you can.

I: Definitely.

R: I think it's not always just about mainstream care either. I think that to look at a person as a whole you have to think about alternative therapies or complimentary therapies I think they call them. So massage, aromatherapy and taking people to the mosque if they're Muslim - looking a bit outside the square of what we traditionally do in hospitals.

I: So accommodating for the patient and their previous lifestyle, is that what you're saying?

R: Yes.

I: Yes, excellent. So some people might find it challenging to define whole of person practice. I'm just wondering if you have any suggestions as to why this might be?

R: I think there's always a tendency to focus on the problems and not the person. So if that person isn't walking then that can sometimes become the whole focus of the entire team.

I: Sure.

R: Sure those things have to be prioritised - that might be one of the main goals that the patient has but at the same time you still have to look at all their other functions as well.

I: Definitely. So putting them in context I guess?

R: Yes.

I: No worries. So what do you think might be the opposite of whole of person practice.

R: I think what I just said - just focusing on the problems and the staff deciding what the main goal of rehabilitation would be. It might not be what the patient is wanting to be rehabilitated in. Just looking at individual problems, not the person as a whole.

I: Sure. So does whole of person practice look different for children and adults in brain injury rehabilitation?

R: Yes, this is a hard one for me because I've never looked after children in a rehab setting. I thought the principles would probably be the same but the - maybe the delivery of care would be different. I think it would be basically the same but there would be a lot more family involvement and - yes. I think the basics are still the same.

I: But a little bit hard to say whether it's actually implemented, I guess?

R: Yes.

I: The next few questions ask about how you might achieve whole of person practice in your setting at [organisation name withheld]. So how do you achieve whole of person practice in the nursing discipline within [organisation name withheld]?

R: I think that we involve the entire team in our recovery process. We talk about recovery in a mental health sense, like returning the patient to their previous environment and trying to introduce things that they previously did. So we involve the entire team in our recovery meetings, as well as the patients. I guess it goes back to the assessment, planning, implementation and evaluation - we're always assessing anything that - any part of their program is always assessed. Then we have to re-plan with the entire team that it's implemented with the nursing staff and the therapists and then always evaluating again with the entire team and the patient.

I: It sounds…

R: That's just an ongoing process that never really stops.

I: Yes, that sounds really good. I'm just wondering by team who you're referring to?

R: Physiotherapists, social workers, occupational therapists, psychologists, the psychiatric registrar, music therapy, the nurse unit managers involved, dieticians and the nursing staff.

I: That's a lot of people. [Laughs]

R: Yes. The recovery model refers to - it's driven by the patient, it's what they want. So it has to come from them. Then the rest of the team get on board and try to make it happen.

I: Excellent. So with the rest of the team, how do you see other practitioners implementing whole of person practice?

R: They by participating in the team meetings and the recovery meetings or the case review meetings we all get a chance to share information at our meetings. We have a system of data collection called [name withheld]. I don't know if you have heard about that?

I: I haven't heard of that one, no.

R: It's a mental health specific data collection online. We're required to use it as mental health practitioners. So if we've got a care plan, it's a general care plan, that's sent out - the nursing staff would generate the basic care plan. Then it's sent out to the entire team. So the social worker might add their bit about maintaining contact with the family or doing social outings. The physiotherapist might add their bit about regular - daily physiotherapy or whatever. So the entire team gets to contribute. Then it's taken to the patient and they either agree or disagree or get to takes things out or add things.

I: That's fantastic that it's taken to the patient to have their input as well. I guess it's really involving them in their goals and in what they want to achieve…

R: That's right.

I: …and how they think things are happening.

R: Yes, that's right. They're they participate in the assessment part and then we take it away and transfer the assessment into the goal - without getting too complicated. Then we take it back to the patient and read through it and generally they're quite happy to participate in everything we suggest.

I: So it's really…

R: They usually want to add more.

I: [Laughs]

R: Weekends home and more outings. [Laughs] But generally it works out well.

I: Yes. So it's really striving for a more collaborative approach I guess.

R: Definitely. We have a real team approach here. I know in a lot of other mental health facilities they're all mental health practitioners, they're not role specific. But here we work very role specific, even though we overlap with each other all the time, we all know what our job is and that's what we focus on with the patient. But by sharing information we make sure that everybody knows what everybody else is doing and what we're all working on together.

I: Yes, excellent. So there's always challenges or difficulties in providing rehabilitation to people. I'm just wondering what external factors do you think might influence the real world delivery of whole of person practice?

R: I guess - there's always budget and resources which - I mean, we really struggle to get any resources to help with our patients here. The answer is always that no, there's just no money. The other thing that I've identified in our acquired brain injury unit, which makes it different to the other brain injury units in Queensland, is that we employ assistants in nursing. They're basically untrained staff, so they're not - they just know the basic - how to wash and shower a person, those sort of basic cares - make a bed and that sort of stuff.

So their focus of care is very different to anyone who's had some training. They find it very hard to let the patients do things for themselves. They tend to want to wash them all quickly and get it over and done with or help them make their bed so that it's done. Whereas some who's trained may let the patient was as much as he can by themselves and choose their own clothes, make their own bed - it mightn't be perfect but at least they've made it themselves. Whereas I think the untrained staff do present a barrier to rehab here. I know the other acquired brain injury units have enrolled nurses and registered nurses. They don't use any assistants in nursing.

I: Are the differences between the enrolled and the registered nursing, they have the training?

R: Yes, they do.

I: They do. Okay. So at [organisation name withheld], there's some untrained nurses there that may not take the time necessarily to help the patient do it themselves?

R: That's right. Yes, they're task focused rather than patient focused. So we have issues - they'll have all the patients in their pyjamas at 3:30 in the afternoon, because they see themselves as being really efficiently - you know, we've got everyone ready for bed. [Laughs]. When in reality people don't usually get into their pyjamas…

I: At three o'clock.

R: …at 3:30 in the afternoon. That's just an example but…

I: No, it's a great example.

R: They put people to bed at 6:30 in the - at night because they think that they're going to get a really good night's sleep if they go early or I don't - it's not - whereas a registered nurse might say - give them a choice. Do you want to watch this TV program or do you want to do something else. You don't necessarily just have to go to bed at 6:30 at night. They just have- yes. They have a different focus. They're task focus rather than…

I: Person…

R: …patient focused.

I: Yes. When you mentioned resources, were you - did you mean financial as well as objects, as in…

R: Both.

I: …medication and - a bit of both?

R: Yes. Medication's fine because the patients pay for that themselves. But actual resources to facilitate things like a cooking group. The occupational therapist says that it's a vital part of her role to assess people's skills in cooking and kitchen safety and all that sort of thing. Yet there's no budget to…

I: Allow…

R: …provide the ingredients. We have to share a kitchenette between two units which sometimes gets - it's not ideal. Whereas if we actually had a kitchen in both units then it would be more therapeutic and beneficial for the patient, as an activity.

I: More accessible as well I guess.

R: Yes. That's right. They could do it more often. There's less distractions. They'd be more relaxed in their own environment. As it is now they go next door and use the kitchenette there and it's not ideal. But that's something that we have put in a submission for and it's never - it's always - the answer is always it costs too much. The same with a computer. We'll do a business case for the patients to have a computer that they can use because - sending emails to family and - that's what the normal population do these days. They don't write letters or - there's never any money to buy a computer for the patients to use or there's never just basic things really for the patients sometimes.

I: That just won't be addressed.

R: No.

I: In terms of contextual barriers or factors that may influence the real world delivery have you noticed anything along the lines of family or social networks for example?

R: Just say that again?

I: In terms of contextual external factors that might influence the real world delivery of whole of person practice, for example maybe family or social networks that the patient may have around them, have you seen anything or can you think of a client example where contextual external factors may be influential?

R: I guess for our patients discharging them to a safe place where they have appropriate support is always an issue - is that sort of thing you're wanting?

I: Yes.

R: Because we - they reach the rehab goals but a lot of our clients still need supervision or assistance with some things. But there's just no support in the community for them. They just don't fit into any - they're not in aged care because most of them are too young. They don't receive - for instance we've got a spinal patient at the moment and she's just lost all of her funding for community care by coming here.

I: Oh no.

R: She had a 40 hour package while she was in the spinal unit but when she came to the acquired brain injury unit she lost those 40 hours because she was in care here. It's very difficult for us to get that aftercare follow-up…

I: Yes, so once they're discharged.

R: Yes, they don't fit into the mental health service quite often because they're not acutely unwell, the community mental health teams won't pick up most of our patients, even though we refer a lot of them to them, they don't see them as meeting their criteria. So they're just discharged and we hope for the best, you know? We try and put in as many supports as we can but it's not ideal.

I: Yes. I guess being a lifelong injury as well…

R: That's right.

I: …it can prove massive implications.

R: That's right, yes - and having suitable places for them to be discharged to. There really just isn't anywhere. It's hard - if they can't go to their family, then the real most obvious place is aged care.

I: Poor things when they're not - when they're quite young as well.

R: Yes, that's right.

I: We have spoken a little bit about this but I'm just wondering whether there's anything more you'd like to add. Rehabilitation goals for patients may change across settings, across the acute, subacute and community. I'm just wondering how exactly might whole of person practice change across these settings in relation to the goals that the client might have?

R: I guess when they're in the acute service they're really just looking at - quite often it's just survival and are they going to live.

I: Yes.

R: So their needs are very medically based and their goals are basic things like how are they going to eat and drink, go to the toilet. Are they going to be able to mobilize. Whereas when they come to extended care our goals are much wider and more to do with actual quality of life. Also looking at - yes, I think in acute areas they look at the bio - biological and psychological factors first. It's almost like a needs base - like they're being triaged I guess.

I: Sure.

R: As they get better they become more complicated and less urgent - their needs.

I: Somewhat expanding I guess is they're able to - like you said - they're stable - they're stabilized and able to try and rehabilitate in a functional capacity.

R: Yes, that's right.

I: Excellent. Is there anything more that you might like to add that we haven't talked about today?

R: No, I don't think so.

I: Pretty much covered it all?

R: Yes. I hope so. Will I get some feedback from this?

I: Yes. I'll just stop recording if there's nothing more that you'd like to add?

R: No there's not.

**Transcription ID7:**

I: Thank you for agreeing to participate in this study examining brain injury rehabilitation. I understand you have read the participant information sheet and have consented to complete a short interview. To remind you, our conversation will be tape recorded; however any information you provide will remain confidential and will not be disclosed to anyone other than as part of a summary report. Which means results will be aggregated and your answers will remain non-identifiable. Your participation is also voluntary, and you are free to withdraw from the project at any stage. Do you have any questions?

R: No.

I: All good to go? Firstly, can you tell me what are the guiding principles of brain rehabilitation within your practice?

R: The guiding principles would be to work with the patients to help them achieve their goals in regards to physical return.

I: That sounds really interesting, could you perhaps tell me a bit more using, maybe, a recent patient as an example?

R: We have a patient in at the moment that had a skateboard accident. His main goal at the moment is to return to walking, he’s having quite a lot of difficulty with particular movements and positioning of his feet.

We have been working with him doing some stretches, some mobilisation to get him up so that he can walk. He’s progressed to where he is going out with his family on weekends; he’s been able to integrate back into the community, so he is seeing friends socially. He’s going out for dinner and he is able to get around over short distances with walking.

I: In your opinion, do these principles differ from the general rehabilitation principles of brain injury?

R: I don’t think so.

I: No? The next few questions will ask about your thoughts on what is whole of person practice in brain injury rehabilitation. From your experience, how would you define holism in brain injury rehabilitation?

R: I would define it as treating the whole person in all their aspects. You’re considering their previous lifestyle, their previous social supports, their family, their previous occupation, their previous functional level, fitness level, all those things. You have to take into account the effect of the injury and what potential, either cognition or behavioural issues are going to impact on how they recover.

Similarly with the family, needing to know their history or their… what they are bringing to the table in regards to the rehab so that you can help to work with them as well, to get the best outcomes.

I: You’re saying it’s not just about the patient, but the people around them as well and all those environmental factors that may come in?

R: Exactly yes.

I: Some people may find it difficult to define whole of person practice. Do you have any thoughts as to why this might be?

R: I think sometimes you can lose… you get so focussed on one particular thing that you sometimes lose the bigger picture. But I think once it comes up or is mentioned in any of the conversation, it’s generally fairly easy to then reintegrate it into what you are planning to do or trying to do with people.

I: What do you think might be the opposite of whole of person practice using an example of what that might look like?

R: Opposite would be very specialised, specific treatment. If a patient had a knee injury and they came in and all I did was focus on the knee injury and treat that without any consideration for what they were using the knee for, what they needed to be able to do with it, any other problems that were in the body, any other health issues, family support that was at home, who was going to be able to help them with the rehab, all those things. They just came in, I did the treatment and they left.

I: In your opinion, does whole of person practice look different for children and adults in brain injury rehabilitation?

R: I would say yes. Just simply the impact of, one is the family dynamics and the, I’m going to say, the power of the patient to direct their own care is somewhat different. In the two age groups, the families may have a bigger role with the paediatrics than with your adults.

I: And with what you said before, is the family being involved in the rehabilitation quite important as well?

R: I think so yes.

I: The next few questions ask about how you might achieve whole of person practice in your setting, over in [name withheld]. How do you achieve whole of person practice in your discipline at [name withheld].

R: Not so much specific to our discipline, but within the unit we have family meetings. Once the patients are actually admitted, within that first couple of weeks we do meet. The family meeting, family is a lose term for interested parties and people that would like to come along and be involved in their rehab. We also have an open door policy for the gym, so we’re more than happy to have families come in and be here during treatment sessions so that we can coordinate ongoing care with them.

We do things, which again, isn’t probably any different to other units, but we do things like preparing for home visits and doing training with families and all that in regards to car transfers and wheelchairs. That’s probably the main thing specific to us that don’t have any other input from the rest of the unit.

I: How do you see other practitioners implementing whole of person practice then?

R: Similar to that. Other things that we do here in the unit, they do have what we call a Peer Support Group where all the patients can get together, and it’s an opportunity for them to express concerns. So again not only just treating their physical issues or cognitive issues or whatever we have assessed as being a problem.

It’s a chance for them to voice from their perspective what they are having difficulties with. Whether it be something as simple as wanting to change the meals because they are not happy with the meals they are getting, wanting to have variety with their shower times, it’s an avenue for them to have a voice which is helpful for them.

I: There are always challenges or difficulties in providing rehabilitation to people.

R: Yes.

I: What external factors do you think might influence the real world delivery of whole of person practice?

R: In regards to, probably some of the limitations can be, just the times that we work. We work eight to four, as do a lot of families and a lot of the support from patients who generally tend to be here in the evenings so a lot of times, just even being able to touch base with them because of the times can be a little bit of a challenge.

In regards to having any of your normal supports out in the community as well, just your general funding issues, access to things if you’re disabled all those things come into it as well which make it a little bit more difficult. I think because of the nature of the injury as well, generally what tends to happen is they do lose a lot of their social supports and a lot of their social links which then leads into that more isolated ongoing rehab for them once they’re in the community.

I: Yes, it does make it difficult doesn’t it?

R: Yes.

I: Can you provide an example of when you feel that you couldn’t achieve whole of person practice and why?

R: Some of the times in here I would say, because our patients may have a psych history prior to actually coming into the unit. So sometimes that actual psych side of their presentation may have a big impact on what is going to happen rehab wise. I think because of my lack of knowledge in that area, sometimes I feel that I’m a little bit limited in what I can offer for both the families’ perspective and for the patient.

I: I hear what you’re saying, definitely. I know you’ve spoken about [name withheld] as a setting, but just a little bit more from the physio point of view, what might be some challenges within the physiotherapy team, like just your discipline?

R: Challenges?

I: Bit of a tricky one.

R: Yes, I’m just supposing, working out work load sometimes can be a bit of a challenge, again, because of the patient mix it can differ between quite heavily dependent patients needing a lot of physical input up to the very mobile, very well, physically, with mainly cognitive issues. That can be a bit of a challenge sometimes if it is too far either way, it can frustrate the team a little bit.

Burn out, I think it is quite a difficult area to work and I think with all the cognition and the, as you said, that longer term involvement and the need to be involved with family and have, as you said, that holistic side can be quite draining on people from a work perspective.

We’re pretty lucky turnover wise, we do have staff who are very committed. We’re lucky we have a core group who do stay within the unit. Succession training is always a big problem, just to train people up to be aware of the issues within the brain injury population so they are going to be able to address them.

I: Definitely, and having that experience with the population as well.

R: Exactly, yes.

I: So rehabilitation goals for patients may change across settings from acute, sub-acute and community. Do you think this is the case?

R: Yes, I do. I think that’s appropriate because, I think, there are different priorities and different needs in each of the settings and I think your focus does change a little bit. In the acute, it is more managing the medical issues, making sure that they are not going to develop any bad habits or compensations or a lot of prevention until they get to that point where they can be actively involved in rehab. I would say then, through the rehab stage, it is much more of a joint between patient and therapist as to where they are heading, and their goals. And then, much more in the community that, that, I was going to say control but that’s not the word. [Inaudible] …goes over, back to the patient again, they’re the person that then has to be driving their rehab. They are the person than then has, often, a lot more understanding of the difficulties they are going to face, once they actually are in the community.

Things can become a lot more real, in regards to the problems that they are having, and so their focus does change. Things that they might not have thought were a big problem when they were inpatients, or they had a lot of support, can become a big problem once they get home and are trying to be independent. I think it’s natural that it would change.

I: Yes. For patients, this is the final question, for patients who may fall through the cracks a little bit, what do you think happens there?

R: What do I think happens?

I: Yes, service wise?

R: I’m not sure what you’re asking. My thinking is that they deteriorate; they don’t have access to some of the supports, some of the services that are out there. There’s a potential that they could…

[OVERSPEAKING]

R: …deteriorate. They might be quite fine without it, might have no problems whatsoever. It may have been a choice to fall through the cracks, as you say. But yes, I would potentially say that they would be at a disadvantage just from the fact that they are not aware of the options is the best word, that they do have, more so than actually being involved, it’s just knowing that those options are there.

I: Is there anything else you would like to add that we haven’t talked about today?

R: No, I don’t think so.

I: It kind of covers everything.

R: Yep

I: No worries, I will stop recording then.

**Transcription ID8:**

I: So [name withheld], thank you for agreeing to participate in the study examining brain injury rehabilitation. I understand you have read the participant information sheet and have consented to complete a short interview. To remind you, our conversation will be tape recorded. However, any information you provide will remain confidential and will not be disclosed to anyone other than as part of a summary report, which means results will be aggregated and your answers will remain non-identifiable. Your participation is also voluntary and you are free to withdraw from the project at any stage. Do you have any questions?

R: No.

I: Okay, excellent. So firstly, can you tell me what are the guiding principles of brain injury rehabilitation within your practice?

R: Basically it's about maximising recovery, so adults who have had a brain injury. Regaining independent functioning in all areas. We work from a client centred client goal based program and our focus is on community re-integration and engagement again with community activities.

I: Excellent. Do you feel they are different from the general rehabilitation principles of brain injury?

R: No. I guess the focus is different depending on the client goals which change as recovery occurs, but the general principles are still the same about maximising recovery and independent functioning.

I: Yeah, definitely. So the next few questions ask about your thoughts on what is whole of person practice in brain injury rehabilitation. From your experience, how would you define holism in brain injury rehabilitation?

R: Okay. It's working with the person in their own environment, working across all areas of their lives, working with their supports and their networks, which may be informal networks such as family and friends but also other professional supports that they have.

I: Excellent. Some people might find defining whole of person practice difficult. Do you have any reasons or thoughts as to why this might be the case?

R: Possibly it's to do with our training in different disciplines where the focus might be on a specific area of functioning and it also might be about workplace and protocols and the type of job you have and the type of area that you're working in.

I: So from a service level, what are your thoughts there?

R: In what way?

I: Across different settings; presentation along the continuum.

R: It may be easier to work in a more holistic way once somebody's back in the community living at home with their families, but I still think that therapists and professionals working in a rehab or hospital inpatient setting, they still attempt to do that, working across teams and working with the families and significant others.

I: Okay. So the next question may be a bit similar as to what we've already spoken about, but I just wonder if there's something more you might be able to add to it. What do you think would be the opposite of whole of person practice?

R: I guess that would be just working with the person in one area of functioning and not considering the impact on other areas that that person's functioning in life.

I: Okay. Does whole of person practice look different for children and adults in brain injury rehabilitation?

R: I don't think so apart from the fact that decision making would be with the child's parents, parents or guardians where many of the adults we work with are able to make their own decisions and give consent.

I: Okay. So the next few questions are asking about how you might achieve whole of person practice in your setting. So how do you achieve whole of person practice in your discipline as an OT and in your setting and can you please give me an example?

R: Okay. We work in a trans-disciplinary team, which means the team is made up of a number of different professionals but we work in a case management role and we work in a similar way to each other, but we draw on our discipline background to inform our practice. We also draw on the discipline professional background of other team members as needed, so we each have a caseload. We work in a community based setting where we engage with the individual with the brain injury and the networks as I was saying before, informal and formal networks that the person has. It's probably easier in a community setting to look at whole of person. From our initial interviews we do a very broad psychosocial assessment with a person and look at all areas of functioning, where they're having success and where they might be having difficulties. From there we work with the individual about setting goals and working on a plan for their rehab, so it's very much goal directed and client focused in that way. Is that enough?

I: Yeah, that's perfect. How do you - this one might be a little bit tricky to answer - but how do you see other practitioners implementing whole of person practice?

R: I guess as I said it is easier in a community setting and our other team members I see working, like they work the way I do. Other practitioners that I work with around a client, I guess it's about communicating with the whole team that's working with a person and it's about considering - if that professional isn't working in a particular area of functioning or around a particular goal for that client, that they're still aware of that need and might link the person with appropriate supports.

I: Okay. There are always challenges or difficulties in providing rehabilitation to people. What external factors do you think might influence the real world delivery of whole of person practice?

R: Okay. I guess some of that is about your relationship with the client and the willingness of the client to engage in a rehab program and their disclosure about particular things that are really important or you think are important to a whole of person practice. The client may not be willing to disclose that information. I guess the other thing is the engagement of the family and significant others and people in the network. People may not always be willing to participate and engage. The other issue might be the level of interest or information or knowledge someone else has about brain injury and their understanding of say the difficulties a person might be having, so that I guess if people lack some of that understanding they might not see the need for a particular intervention or a way of practice. I guess the other thing about particularly family members is their level of energy or their capacity to actually engage and assist their family member. It might be that there's so many things that they've got to cope with they would prefer to leave it to somebody else if you like.

I: What about from a service point of view as well?

R: Well, it might be the history of a service and how that's delivered. It might be the model that the service is built around in relation to service delivery. We work around community based rehab model and of course that model there is depending on the setting. So the who definition is very broad and that's the one we tend to strive for but in reality it's often less than that.

I: So what would the challenges be within your discipline?

R: Have in OT?

I: Yeah.

R: I guess what I've already said about the engagement of the client or their willingness to participate. That's a challenge sometimes. But our discipline, I guess we're trained in holistic practice and I haven't found any difficulty in working in that way at all.

I: Yeah, no worries at all. So rehabilitation goals for patients may change across settings. Do you think this was the case?

R: Yeah.

I: So you've sort of mentioned earlier that you think whole of person practice may be more implemented in the community settings. Do you feel there's a different way that it could be carried out in the acute and sub-acute settings to make it more like the community?

R: I haven't worked in that area personally so I guess I would be reluctant to say how I could improve something I don't have direct experience in. But I do know that the goals are different because of the different stages of recovery, so an inpatient setting the focus might be more on return to physical functioning and ADL's and that kind of thing, where we're seeing people further down the track where those goals are being achieved and it's more about the re-engagement with community.

I: Yeah, no worries at all. Is there anything you would like to add that we haven't talked about today?

R: No. I don't think so. That's it.

I: That's about it. No worries. I'll stop recording now.

R: Alright. Thank you [name withheld].

**Transcription ID9:**

I: Thank you for agreeing to participate in this study examining brain injury rehabilitation. I understand you have read the participant information sheet and have consented to complete a short interview. To remind you, our conversation will be tape-recorded however any information you provide will remain confidential and will not be disclosed to anyone other than as part of a summary report which means results will be aggregated and your answers will remain non-identifiable. Your participation is also voluntary and you are free to withdraw from the project at any stage. Do you have any questions?

R: No.

I: To begin, can you tell me what are the guiding principles of brain injury rehabilitation within your practice?

R: I think the main thing is that no two brain injuries are the same. In fact I've heard it said if you've seen one brain injury, you've seen one brain injury. I think the important thing about it is that because it's different injuries happening to different people in different contexts – you can't have a one-size-fits-all approach. Your approaches have to be individually tailored and flexible.

I: I'm also just wondering whether these guiding principles of brain injury rehabilitation differ to the principles within your practice as a rehabilitation coordinator.

R: I think I was probably talking about those. I'm probably talking about a bit idealistic. I think those should be the key principles. I think in general the brain injury rehabilitation principles are to restore, as far as possible, people's abilities to function and that tends to have a little bit of an impairment focus so it's probably more in answer to question one. With ours, question two is within our own practice we're often seeing people whose recovery is either slowed or plateaued and so it's really more about where to from here given that there are residual, possibly permanent, impairments. So I guess our focus in our end of the practice is more about participation in the presence of existing impairments rather than the recovery of function or the reduction of impairment.

I: So just to make sure that I've heard you correctly, it's more about helping the person adapt to their environment around them in order to facilitate a way of functioning with their injury.

R: No.

I: Not quite?

R: I didn't say that exactly. I guess what we tend to focus on more is looking at changing the environment around the person so that they're more able to access it or interact with it.

I: That makes a lot more sense. The next few questions ask about your thoughts on what is whole-of-person practice in brain injury rehabilitation. From your experience, how would you define holism?

R: It means the whole person and I guess that extends beyond just the physical and cognitive functions of people into their emotional and psychological and social functions as well.

I: Could you possibly provide an example using a patient?

R: Sometimes we have clients who have made what would seem on the outside to be a very complete recovery and they're able to get back into a lot of the things they were able to do before but yet they are maybe feeling psychological distress because they perceive themselves to be a different person than they are before. It might not be evident to other people but it's evident to them. So we would focus on what's important to them.

I: What they describe as their goals, would you say?

R: Yeah, I guess so. That's more client-centred practice, I guess. Holistic is making sure that you're not just chopping a person up into parts and saying your hand's not working, let's work on your hand or your balance isn't much good, let's look at your balance. Holistic practice is looking at what are you wanting to achieve in your life and what can I do to help you get there.

I: My next question is why might it be challenging to define whole-of-person practice.

R: I guess because in rehab circles the professionals are trained up in [size] so they'll all have different ideas about what is holistic practice. If you come from a professional background you might not have all the skills to deliver all facets of holistic practice and so I think sometimes people tend to focus on what they know and maybe don't ask questions about the other stuff.

I: What would you think would be the opposite of whole-of-person practice and could you provide an example?

R: It's impairment based, focusing on impairments. One example I can remember is a referral that we had for someone who'd been to see a multidisciplinary rehab team and they said you've got a high level of balance and coordination problems so we'll offer you that and the person said well actually I'm quite happy with my balance, I was actually quite happy with my balance and coordination, it's adequate for what I need; what I really want is work on my speech because I help my husband in the business and I need to be able to talk to people on the phone. So that was the opposite of holistic practice to me in that the professional assessed and decided what the problem was, proposed a treatment plan without discussing with the client what was their priority and what were their needs.

I: Within a functional capacity in their life and what they needed to achieve?

R: Yeah, what did they particularly need help with; didn't ask them what they needed help with. They, as the professional, did an assessment, identified some deficits and said I'll help you with those without actually checking that that was a priority for the client.

I: Does whole-of-person practice look different for children and adults in brain injury rehabilitation?

R: Probably it does but probably shouldn't. I think with kids professionals are much more likely to recognise that the children are a part of a family system and engage with the whole family. Once people become adults and there's changes in who can give consent and there's issues to do with confidentiality, I think the tendency then is to focus more in on the individual and perhaps not include family and friends and other people who would interact with that individual. So I suppose the holistic involves more than working with just the person; it involves working with their whole environment which includes the people in that environment. I think we probably would tend to naturally be a little bit more holistic with kids than with adults because of that whole health professional/patient relationship.

I: To include the family as well as a minor?

R: Yeah.

I: The next few questions are about how you might achieve whole-of-person practice in your setting. I'm just wondering how you go about achieving whole-of-person practice within [organisation name withheld].

R: I guess it starts initially by making sure that we build a good rapport with the person so that there's a trust, whether they're prepared to discuss things with us and then no matter what our background, we all undertake the same holistic biopsychosocial assessment process. So we actually ask questions in all areas of people's lives, extending into emotional, psychological, financial. We look at not just the changes because of their brain injury but how those changes are impacting on all areas of their life. So we've all got the same - I suppose we're looking at them with an interdisciplinary eye. It's called case finding. We're actually looking for areas where we might need to engage people with other skills than our own and make sure we connect the person with those other professionals if that's required. It might not be other professionals; it might be other services. I guess the most important thing is holistic biopsychosocial assessment. If you don’t ask the right questions, you won't get - if you don't ask the questions you won't get the answers.

I: Yeah and I guess it's having a real collaborative approach.

R: Yes and we often involve more than just the person with the brain injury in the initial assessment. We often include family members if that's okay with the client.

I: Just talking about the biopsychosocial model, I'm just wondering if you'd be able to provide an example using a recent patient.

[Pause]

R: I had a recent new assessment of a fellow who had a brain injury a couple years ago. He was a sole carer, a custodial parent for a 12-year-old boy. When I spoke to him the main things that were worrying him was his parenting. He felt as though he had not been sufficiently good at setting limits or [unclear]. I think his words were he'd utterly spoilt his child and the child was now not taking much notice of him. So basically, even though there are lots of other issues, things to with household maintenance and budgeting, that were quite evident, those weren't the things that were identified as a priority by him. So what I'll be looking to work on with him is the parenting support because that's what he said was important to him.

I: There are always challenges or difficulties in providing rehabilitation to people. I'm just wondering what external factors you think might influence the real-world delivery of whole-of-person practice.

R: I suppose there are quite a lot of contextual factors if people don't have good natural supports and I think one of the biggest problems is lack of resources or fragmented service systems. You know what they need but there's no service that provides that or if there is a service then you can't access it because they're not eligible or they don't have any money. So quite often it's lack of services or lack of ability to access services or even lack of funding. I suppose the other factor that can occur is changes in the person themselves, particularly if there's changes to their insight. If people don't actually understand that they either have an issue or are contributing to a problem, they don't tend to be able to address that problem. They attribute it to other people around them and so lack of insight can be a big barrier to holistic practice.

I: How would you go about increasing a person's insight? I'm just wondering.

R: I guess sometimes we support people to try something which we don't think that they're going to be able to achieve. The benefit of that is that it's validated that we're prepared to see them as guiding the process and if it doesn't succeed, if they're not able to achieve what they thought they could, we're also there to help support them through the recognition that that's the case. I suppose that measured risk is a really good way of helping people develop insight and so that means also not - helping people break things down. If they're saying they want to enrol in a university course and you think I don't know whether that's going to happen, if they've got the abilities, you might start them, say it's been a while since you studied, how about you try this TAFE course first and if that goes well then maybe think about uni next year or whatever. So we'd often then help them get started on something more bite-sized and doable and see how they go. I suppose there's two benefits of that. Sometimes people surprise you and they can actually do something that you didn't think that they'd be able to. Otherwise, if they can't manage it then you can be there to help them, to support them through the recognition that their abilities have changed and also help them to refocus on something else.

I: Thank you for that example. When you mentioned resources earlier, was it just in relation to services or more generally?

R: I suppose it's in general. There's a lot of variability in the range of services available to people depending on where they live. Also access to services can vary a lot as well. You might have services in different regions of the state that are funded under the same model but one will accept a certain type of client and one won't. One of the big issues that we have is people who've got funding, for example for lifestyle support services or what have you, they can often live more the way that they would choose, i.e. independently with support, not with family members, than people who aren't able to access that money. So people who, for example, are compensable through motor accident insurance or have income protection insurance so they're not having to subsist on pensions and things, they can often get a lot more going in their lives than people who aren't, people who are just on Centrelink benefits and trying to access generally-available community services like HACC services and other services. It tends to be very difficult to find enough support for them. So money is important.

I guess another really thing that's important is functional informal support networks. They're really important. People who've got supportive families and supportive friends who can actually get involved in rehabilitation programs and help a person apply what they've learnt in therapy or in rehab, they tend to do a lot better than people who perhaps come from less functional families. Families where there might be ageing parents, problems with substance and drug abuse, other people with disabilities who live in the family, low socioeconomic groups; all of those factors can help make it harder. People who've got a lot of those factors in their background tend not to do as well. Our approach is that recovery's a lifelong thing; it's not a sort of mythical two-year timeframe [unclear] and if people keep going, they can keep improving, albeit slowly but sometimes if they don't have the support to do that nothing happens. If they do have the support to do that, all sorts of things can happen. People can continue to improve years and years down the track.

I: Like you said, really surprise you with what they can do.

R: Yeah.

I: Rehabilitation goals for patients may change across settings. Firstly, do you think this is the case?

R: Yes, I think when people are first injured, rehabilitation goals between patients, as they are then, and health professionals are very closely aligned. The focus is on restoration of function and minimisation of disability. So basically everyone's focused on getting back as close to - their recovery. It's recovery focused. Then as they proceed along the rehabilitation continuum to the secondary rehab area where, if it's evident that there's going to be some residual impairments, the focus then switches onto adaptive strategies and equipment and those sorts of things that will help them to keep doing the things that they used to do even if they don't have the same abilities that they used to do. Then once they get out into the community setting, the focus is really much more on participation in life roles and social roles that are unique to the person. So it's how to get back into their own lives in the presence of ongoing residual impairment with the adaptive strategies and equipment that they may already have.

I suppose at this point of the continuum we're finding that people are really focusing a lot more on their identity as an individual: who am I, who am I going to be now, I used to be a worker or a bread winner or a student or whatever, now I'm not doing those things, what can I do. It really changes much more to the participation end of the spectrum and that will involve sometimes a lot of our input is at the environmental or the contextual factors around people who are in the community. So it could involve providing education and training to people around them rather than to them themselves and looking at those other sorts of barriers that are not necessarily connected with the individuals themselves; it's the environment. We spend a lot of our time looking at environmental barriers and facilitators and trying to get that side of things working.

I: When you mentioned that it's really about participating back into their community, I guess like you mentioned earlier, these barriers of not being able to access services becomes so fundamental.

R: That's right and I suppose, too, the participation has to be very much directed by the person themselves. They're the only ones that know what's important to them and they're the ones that know what they need to be able to do, too, to fulfil their own family and social roles. We can't presume to know that. So it's really very much at that end of the spectrum it really has to be very client-directed because anything else will mean that they'll disengage from the service and there's no point. If it's not a client goal we don't work on it because if it's not their goal they won't work on it so there's no point wasting everyone's time and energy. Having said that, once you've engaged with a person and have spent some time working on their goals and you develop a good therapeutic relationship or a good rapport, they can sometimes then revisit things that…

I: Initially may not have been open to.

R: Yeah and might be prepared to actually - a bit more give and take once they know that you're not going to try and push them into something that they don't want to do. They can sometimes - things change and I guess also, too, people's needs change over time. There's a transition in their own lives and their relationships change, friendships change, all those sorts of things happen. The extent to which people want to participate can vary over time as well.

I: Like you mentioned, they're the only people who can really drive their rehabilitation. That is so important. Thank you for those wonderful examples. That's all the questions that I have for you this afternoon. Is there anything else that you'd like to add that we haven't talked about today?

R: Probably not. I should've made a few more notes before we talked. I meant to do that but I didn't get around to it.

I: That's okay. It's been very helpful. If there isn't anything more I will stop recording.

R: Okay.

**Transcription ID10:**

I: Thank you for agreeing to participate in this study examining brain injury rehabilitation. I understand you have read the participant information sheet and have consented to complete a short interview. To remind you, our conversation will be tape recorded. However, any information you provide will remain confidential and will not be disclosed to anyone other than as part of a summary report - which means results will be aggregated and your answers will remain non-identifiable. Your participation is also voluntary and you are free to withdraw from the project at any stage. Do you have any questions?

R: No, that's fine. We can go ahead.

I: Excellent. So to begin, can you tell me what you feel the guiding principles of brain injury rehabilitation are within your practice?

R: So basically to look at patients - how severe they've got injury to their brain. What is the goal of the patient? What sort of support they need and how are we going to do that…

I: Sure.

R: So that's our basic broad guidelines I would say.

I: Excellent. Do you feel those guidelines differ from say, general rehabilitation principles?

R: Yes, sort of - a little bit because the unit I'm working with here is more specific to brain injuries, as you know. Compared to the other rehab setup which is - this one is more specialty so we look more in detail about patient's communication, cognition and their ability to be independent after having a brain injury. So yes.

I: Sure, sure. The next few questions I have ask about your thoughts on what is whole of person practice in brain injury rehabilitation. From your experience how would you define holism?

R: My experience is very little - less - as I mentioned on the questionnaire. I started here since 15 August so it's like probably one or one and a half months. [Unclear] full of rehabs and the exposure previously I had, I had a couple of … from that. All I can say is - as I mentioned before - it's looking at getting patients back into the community, trying to get them back as much as possible as they were before the brain injury. So that will basically include everything as a whole person, rather than just patients having any particular problems. That will make us look at communication, eating, drinking, not having any acute problems - medical problems - and treatment and the social support. Also how functional they were before and then what sort of exercises and therapies they need from an occupational therapists or physiotherapists' point of view. So if we aim trying to see how much functional we can make patients and get to go - make them reach the same sort of state as they were before. So I guess that would make the holistic approach - from my opinion.

I: Sure. So I guess really trying to get them through rehabilitation, looking at their pre-morbid functioning in a way?

R: Yes.

I: Sure. What do you think would be the opposite of whole of person practice? Could you possibly provide an example of what that might look like?

R: Sorry, I couldn't get that question.

I: What do you think would be the opposite of whole of person practice and could you possibly provide an example?

R: Opposite of whole…

I: Whole of person practice.

R: Whole patient approach?

I: Yes.

R: Okay, [unclear] very simple. So if it's like - if anybody looks at a patient as based on their problems - just like treating just the medical problem alone might not solve the issue completely because that can - I can give a treatment with starting some medications. For example, if a patient needs anti-coagulation I can just start them on anticoagulation medication, blood thinners, and then say go away and then come back and see me. But it needs some support as community monitoring and then regular follow up and then advices on how they can go about - so even simple medical treatments may need support and information apart from just medical treatment alone. So I guess that would be an example.

I: Sure. So I guess really bringing into - bringing in with their environments and other disciplines as well.

R: Yes. It's like if I just read the medical problem alone and if I think in the end if everything is fine, then it will come down to whether the patient is ready to go home. Then, is there any support? Is it okay? Is the patient okay to manage himself at home or not? So it's again a prolongation of the hospital stage which is not looked at initially.

I: Yes, definitely. Does whole of person practice look different for children and adults in brain injury rehabilitation?

R: Children might be different given if they are getting older and they are coming into adulthood from being a child - from childhood to adulthood transition - it might be a difficult part. So I think we've got the transition service and support people for that so it's more like giving them more social support, psychological support and parenthood support - especially allowing parents to stay with the kid as much as possible. If it's a really young child so obviously the child will freak out if the child is alone or if [unclear] alone in the ward … medical staff and nursing staff. So it's a little bit different from it. It's a little more extra support will be required for a child in rehabilitation compared to an adult.

I: Sure. Do you feel that it should be that way? I'm just trying to get a sense of what your thoughts are about that.

R: Yes, I guess it's just the nature of itself. So the child, being a child - as I said, they need a bit more access support compared to the adults. I think it doesn't make - it won't make any - if you give the right support it doesn't make any impact on the rehabilitation process as such.

I: Yes, definitely. The next few questions I have ask about how you might achieve whole of person practice in your setting at [organisation name withheld]. I'm just wondering how you go about achieving whole of person practice in your discipline and setting?

R: Yes, sure. So we basically do the meetings often - it's done every week on a Tuesday. It's just to make sure that the patient is getting every sort of therapy - whatever they require, whatever their need from each therapist. There is progress happening, there is support being provided and if at all if there's any new issues it's being addressed. So it's sort of a constant update, not just on the meeting perspective alone but also it's - all the other professionals are approachable and you can [unclear] anytime to clarify things. If there is any hindrance interfering with the rehab process - so social workers and all the therapists along with the medical team. So we make sure that everything is progressing fine so that, as I said, the patient is getting every opportunity and therapy so that he or she can go back to their usual pre-morbid function level.

I: Sure. It sounds like it's a really collaborative approach.

R: Yes, exactly.

I: Yes, excellent. Just along those lines, I'm just wondering how you might see other practitioners implementing whole of person practice?

R: In particular to rehab if you ask me, I'm not sure because this is my first rehab registrar position so I'm just getting my feedback from this one.

I: Yes, that's fine.

R: The previous ones which I have worked at they're general rehab settings. I guess I think we could do the same sort of plan in general in most of the rehab settings. So I guess there shouldn't be much of a difference except for when people have a brain injury, they have more disabilities regarding their cognition, communication, memory and things like that - which I looked at in detail - and specific therapies are given by the speech pathologist and occupational therapist. Given that those issues are addressed here in the brain injury unit - of course we have patients without brain injuries who will be in the general rehab setup.

I: Yes, definitely. There are always challenges or difficulties in providing rehabilitation to people. I'm just wondering what external factors do you think might influence the real world delivery of whole of person practice?

R: Anything can affect it I guess. So the family - if things are considered outside the rehab unit - so a family basically it's social support and financial support. As there are questions on the questionnaire - so basically policies - the rules and regulations - and then how patients can access community. Is there any problem given there are any disabilities or things? So I think there is - there are chances that the factors can come from any department, any side - in day to day life actually.

I: I'm just wondering if you could elaborate a little bit further just using an example perhaps?

R: Yes, sure. For example, if the patient is not - for example the patient is from overseas. As we know, a lot of students come in from overseas and they're studying here. So if they have [unclear] brain injury - yes. So they have, given that the medical cover - the health insurance they take. If they have a significant disability, there are only a few things that are covered or given support in terms of like say, for example, there's no accommodation and no community support accessible. So that is a little bit of a difference from what the patient is as such if the patient is an Australian citizen or a permanent resident or from overseas. So I guess that's the best example that I can [remember].

I: Yes, that highlights it well. Thank you. Rehabilitation goals for patients may change across settings through the acute, subacute and community continuum. Firstly, do you think this is the case?

R: Yes, of course. As patients improve then their needs change. Of course, in an acute rehab setting they need full support and therapy sessions daily which can be tapered down already infrequently as the patient improves. It goes to a community where they get less frequent sessions as required. Also going to the community helps the patient making them more orientated and feel comfortable in their house and their own home. So that sort of transition, I think it's working all right at the moment.

I: Yes. How - seeing as the goals do change across settings in your opinion, how exactly might whole of person practice change across settings?

R: I think the goals don't change. It's the patient's improvement and the level of improvement. That's the one which will be the basic deciding factor. If the goals are set too high, probably the goals need to be changed in that instance. Otherwise, if the patient is progressing well there's usually no problems. So basically they - all the therapy and sessions are directed towards a particular goal. So the problem will be - the problem arises if the patient is not progressing as expected. If a patient is having persisting disability then the goals need to be changed or the care level needs to be adjusted and changed, given what level of supervision of the carer is required in the community or when the patient is getting discharged.

I: Sure. That's all the questions that I have for you this afternoon. Is there anything else that you might like to add that we haven't talked about today?

R: No, I think pretty much you've touched all the aspects I guess - that's really nice, yes.

I: Okay, I'll just stop recording now.

R: Sure.

**Transcription ID11:**

I: Thank you for agreeing to participate in this study examining brain injury rehabilitation. I understand you have read the participant information sheet and have consented to complete a short interview. To remind you, our conversation will be tape recorded however, any information you provide will remain confidential and will not be disclosed to anyone other than as part of a summary report, which means results will be aggregated and your answers will remain non-identifiable. Your participation is also voluntary and you are free to withdraw from the project at any stage. Do you have any questions?

R: No.

I: Okay, so to begin, could you please tell me what are the guiding principles of brain injury rehabilitation within your practice?

R: Within my practice. I suppose - all I could say about that is that I would use, what do you call it, social work framework and practices. I think I'm a bit eclectic because I tend to use strength based practices, some evidence based; what else am I using? Probably task centred and case work approach. Does that seem to answer the question?

I: Yes, yes it does. I'm just wondering if these few principles that you've mentioned, if you believe they differ from general rehabilitation principles?

R: Do they differ. No, I don't think so.

I: No? No worries. The next few questions ask about your thoughts on what is whole of person practice in brain injury rehabilitation. From your experience how would you define holism in brain injury rehabilitation?

R: Holism in terms of practice?

I: Yes.

R: I guess holism for me would be like using holistic approaches. So it's all about, you know, good communication within the team, ensuring the patient and family and whoever support, they're involved. That's what I think about holism.

I: Sure. I'm just wondering if you could just elaborate a little bit more perhaps using a recent patient example?

R: Aha. Okay, so with a patient that would come into this unit for rehabilitation, it would first be discussed with - you know, we would get a referral. It would be discussed in the multi-disciplinary team thing. Then a team would go out and do an assessment. We would encourage the treating team at the facility that the assessment is held to be present as well as family members or you know whoever's involved in their care. So we would collect all that information, bring it back, write out a report. Then it would again be discussed in the multi-disciplinary team. Then as the team finds that, you know, the patient has potential to participate in the brain injury rehab program, then they're offered a bed. Then family are involved. So it's all that, I think.

I: It sounds like it's a very multi-disciplinary collaborative approach.

R: Yes.

I: Also involving the patient's environment, such as family and friends around them.

R: That's right.

I: Sure.

R: Whatever protective factors that they have as well.

I: What do you mean by protective factors?

R: You have protective factors. Sometimes people don't only have family. It's about whoever they're associated with that provides support, whether it be - you know, if it's someone with a vision impairment it could be Vision Australia. If it's someone with diabetes it could be Diabetes Australia. So it's all - I suppose all the stakeholders involved in terms of providing them support.

I: Sure. I guess because each brain injury is different I guess each case is going to be so unique as well.

R: Sure.

I: Some people might find that challenging to define whole of person practice. I'm just wondering your thoughts as to why this might be?

R: Whole of person?

I: Mm.

R: I suppose working in a medical - and this is my view. Because we work in a medical setting, it's all about treating symptoms.

I: Yes.

R: Sometimes that does not - is not always congruent with a person's sense of what wellbeing is, if you know what I mean.

I: Just that there's a little bit more just beyond the injury itself?

R: Yes.

I: To see the person I guess as a person and not just an injury, is that what you're saying?

R: Yes. Sometimes we don't always take into consideration all the other, you know, whether it be environmental factors, whether it be family or other things that they have connections with, you know, that may be able to help in the process.

I: Yes, as a support.

R: Or in the therapy.

I: Sure. What do you think would be the opposite of whole of person practice?

R: Whole of person practice. What would be the opposite of that. [pause]

I: It's a bit of a tricky one.

R: Yes. I'm trying to think. Rather than the whole of person practice. Hmm.

I: Maybe providing an example might be an easier way to summarise.

R: Yes. I'm trying to - I suppose the only thing that could come to mind is really treating symptoms or you know, without getting patients and family involved. I don't know where I'm going with this.

I: I guess it's really hard to describe as a social worker because your work does involve a lot of the families and a lot of the systems around the person.

R: Yes. It's kind of like, very, very - it's sort of a holistic approach I suppose. I suppose not getting, you know, not being very collaborative when patients and families are involved can be the opposite that I would see.

I: Yes.

R: Where it's about okay, I'm the professional. I feel that after assessing you this is what you need and this is what you have to do. You know, that's what I would see as the opposite of whole of person practice, I guess.

I: Sure. So really not including the patients in their…

R: Not very consumer friendly, not - hmm.

I: Yes. Sure, thank you for that. Does whole of person practice look different for children and adults in brain injury rehabilitation?

R: Is a lot different for children than adults. I suppose they would.

I: Could you just elaborate a little bit more?

R: Well, you know, just I think that like children's view of things and their needs are totally different to that of an adult.

I: So really putting whole of person practice, implementing that could be different throughout a person's lifetime if they sustain an injury in childhood?

R: Yes.

I: I guess as they work through the transition period into the adult sector could really change?

R: Well that's what I think anyway.

I: Yes, sure.

R: I think whole of person practice would be totally different. How that practice with children would be totally different to adults as well.

I: Just different needs I guess.

R: Yes, because their needs are different.

I: Yes.

R: But it's also about children would always have their parents, so there's always ongoing consultation with family members because they're not adults, you know, they're young people. Whereas with adults it would be totally different. You could do all those with the adult. You don't necessarily have to have family members there all the time or, you know.

I: As you mentioned earlier it's not always - are family always there? Like sometimes it's Vision Australia etcetera.

R: That's right. Not all - everybody has family support.

I: Sure. The next few questions are about how you might achieve whole of person practice in your setting. I know we've touched on this a little bit. I'm just wondering if you have anything more to add about how you achieve whole of person practice as a social worker at [organisation name withheld]?

R: Yes. So I suppose it's about ensuring that family actually gets enough information about what [organisation name withheld] does. So it's about providing some psycho-education really about what the service provides and to ask them about what their expectations are and all those sorts of things. Then share that with the team, because sometimes you know, it's always - it's different. They would come here thinking - and I suppose it's part and parcel of a grieving process as well that they don't always get the information, you know when they're in the acute setting, about [organisation name withheld] and what it's all about. So it's good to get that done from the get go.

I: Sure. Really providing information…

R: So that we're all on the same page.

I: Sure. I'm just wondering, what happens if a family is - obviously as you mentioned they're grieving and they're not really accepting the situation. Not that you can really accept it, but do you know what I mean? Like, having trouble coming to terms with what's happening.

R: I think just ongoing one on one supportive counselling. Usually they do - like you say they don't accept it, but actually just, you know, having - providing that safe space for them to unpack whatever it is that they're going through at the time is sometimes enough for our families.

I: Sure. This also maybe a bit of a tricky question, but I'm just wondering how you see other practitioners implementing whole of person practice.

R: How do they implement it. By ensuring that patients are heard, that they participate and that their rights are protected. Family are consulted and just using that multi-disciplinary team approach.

I: Sure. There are always challenges or difficulties in providing rehabilitation to people. I'm just wondering what external factors do you think might influence the real world delivery of whole of person practice?

R: External factors. Like outside?

I: Yes, it could be…

R: I can't really understand…

I: It could be anything. It could just in your opinion - so for example some contextual issues or there could be some service problems or something along those lines.

R: Sure. Well obviously like you know, looking at - evidently support services for people with a disability is quite limited. So it comes down to resources really. I find even when they have the brain injury they still fall through the cracks, you know, because some of our patients are really very - they present really well even though there's still cognitive impairment. See when they get assessed that doesn't always get picked up, so usually they don't get the support that they require.

I: Sure. I'm just also wondering, because [organisation name withheld] is a sub-acute setting, do you find that blockage is a problem?

R: Yes.

I: Yes. Could you just elaborate on that one please?

R: Well, in terms of [organisation name withheld], I've learnt - I've only been here two - just over two years. I learnt that initially when the unit went up in 2000, some of the beds - so there are two units. In [name withheld] which is solely ABI, some of those beds were actually given for long term residential care. So there's about 10 beds, 10 or nine beds that are still with young people who have come here from nursing homes. They're going - you know, some of them are still in their 30s, some of them are into their 40s. I think they're going to block those beds until they're in their 50s.

I: Then what will happen to them do you think?

R: Then they can look at nursing homes.

I: So I guess well, while trying to get those few people out of the nursing homes, it is kind of causing a bit of a bed blockage for the new people coming through?

R: It has, yes.

I: Isn't that upsetting that some young people have to resort to the nursing homes situation?

R: Yes. It all comes down to lack of resources, lack of support. There's just not anything out there for young people. Where if somebody needs aged care, you know, there's plenty of places for them to go, but unfortunately nothing like that for young people.

I: So that could be a real external factor that could come into it, sure.

R: Yes, definitely.

I: We spoke briefly about this earlier before as well, but rehabilitation goals for patients may change across settings. So I understand you do agree with this and that it is the case that goals may change. How exactly might whole of person practice change across settings? So through the acute, sub-acute and community continuum?

R: How would that change?

I: Mm.

R: I suppose it shouldn't change.

I: Yes?

R: It's something that should be practised by everybody, you know, throughout whatever city, whether it's acute or sub-acute or community.

I: Sure. I'm just wondering if you just could elaborate just a little bit more, just perhaps highlighting some important issues?

R: About that?

I: Mm.

R: How do they change. Well, I suppose because I've also had experience working in an acute setting, it - you know, it's very busy. So a lot of the intervention - well, this is my experience. When I used to work in an acute setting, it used to be brief and it's all about identifying what their goals are. It's really about referring them on to other services to help with re-integration to the community and all those sorts of things. So I guess when they come to sub-acute and the community, then again, even though it's still whole of person practice, but you know there's a - I suppose we've got time on our side because there are admissions as for slow to recover rehabilitation. So we could delve into these a bit more because that's what we need to do to equip them with the skills to be able to live in the community, you know.

I: Yes. It's like you said earlier as well, like really participating in their recovery in a collaborative way.

R: Yes. So you can do that a lot more in this setting I suppose as opposed to the acute setting.

I: Yes, but - so the underlying whole of person practice shouldn't change really. It should still be there across the continuum?

R: Yes, definitely.

I: Sure. That's all the questions that I have for you this afternoon. I'm just wondering if there's anything else you would like to add that we haven't talked about today?

R: No. I think we've talked about…

I: Covered it all.

R: …everything, yes.

I: No worries. I'll stop recording now.

**Transcription ID12:**

I: Thank you for agreeing to participate in this study examining brain injury rehabilitation. I understand you have read the participant information sheet and have consented to complete a short interview.

R: Yes.

I: To remind you, our conversation will be tape recorded however, any information you provide will remain confidential and will not be disclosed to anyone other than as part of a summary report, which means results will be aggregated and your answers will remain non-identifiable. Your participation is also voluntary and you are free to withdraw from the project at any stage. Do you have any questions?

R: No, that's fine.

I: Okay, so to begin, can you tell me what are the guiding principles of brain injury rehabilitation within your practice?

R: Okay, so I guess there's a couple of different guiding possibly principles or just sort of guiding over our rehab. Some of the things that come to mind are client centred planning. So our rehab is very much on what the clients want to achieve and what the client's goals are. The other sort of principles we use is sort of a strength based approach, so looking at what the strengths are of their client and concentrating on those strengths, focusing on those to be able to help with community integration and being able to work towards what the client's particular goals are. Some of the other principles that we use is obviously within the ICF framework. So we're predominantly - well we are a community based service, so we work with people once they're home from hospital at that next stage of rehabilitation. So we're particularly interested in the principles around participation and activity and getting people back into their community.

I: Sure. It sounds like it's a collaborative approach with the person, would that be correct?

R: Yes, that's right.

I: Sure. These principles that you've mentioned, do they differ from general rehabilitation principles?

R: I guess - I know you sort of touch on some of the questions later on. I guess some of those principles would be in sort of general rehab however, it depends on what setting you're in. So we're very much about clients in their own environment, so I'd imagine that you wouldn't be able to do that in the acute settings. I guess the other - one of the other principles I haven't mentioned that we look at is asset based community development. So we're looking at what assets, you know, every community has certain assets whether it's services or people or things that our clients are living within. We'd be looking at trying to work out what those assets are and trying to help link our clients to that. So I guess it would be quite - I think some general rehab can touch on that but not always.

I: Thanks. The next few questions ask about your thoughts on what is whole of person practice in brain injury rehabilitation. From your experience how would you define holism?

R: So I guess holism, I'm thinking about the whole person and their environment. So rather than working in discipline specific or within different components of a person, whether that might be physical or social or sensory, rather than breaking the person down we'd be looking at that whole person and how they interact and engage within their environment.

I: Sure. I'm just wondering if you could elaborate just a little bit more by perhaps providing a recent patient example?

R: Of the whole person?

I: Yes.

R: So I'm just trying to think about a client I'm working with at the moment. I've got a client that's living at home with his family. Sorry, he was previously living independently before his brain injury. He has now moved back to live with his step mum and father. So I guess when you're looking at whole of person practice with him it's not looking at just his difficulties, his impairments as such. It would be looking at him as a - so rather than just the components of what he does every day or components of his disability, it would be looking at whether he's engaging in his occupation, how he's going about those occupations, how he's interacting with his environment and looking at his goals in relation to that.

I: Sure. It sounds like it's a very collaborative contextual focus, is that…

R: Yes, definitely contextual. So it's about where he is at the moment, where he's living, what sort of - who he's interacting with, his friends, his - you know, his work place, all of that sort of thing.

I: Sure. Some people may find it difficult to describe whole of person practice. I'm just wondering your thoughts as to why this might be the case?

R: I guess, I think like anything probably giving examples is easier. I think everyone's situation is so different that it might be difficult to explain it relevant to that person so that they can make sense of that term. Or they might - it's not very concrete, I guess. It's easy to say to someone something that they can see, so often it's a lot easier for our clients to understand what's happening for them in physio, particularly around their mobility, but it's harder for them to conceptualise or even their family to see what's happening in other areas.

I: Sure. Does whole of person practice look different for children and adults in brain injury rehabilitation?

R: We only work with clients, with adults. I have worked in the area with children as well. I guess probably - well really if you're looking at whole of person and that person within their environment, probably shouldn't be any different however, I guess some of the things that come into mind like capacity and family and those sorts of things; so some of our clients would have impaired decision making capacity and I'm assuming that some children might have those difficulties as well at just even through different stages of their development. So I could imagine that it would look different for children and adults.

I: Sure. Then I guess you sort of mentioned that you don't think that it should but it probably does?

R: Yes, probably not necessarily, like in the strictest form. You know, if you're looking about the person within their environment, participating in activities that they want to do it probably shouldn’t look any different.

I: The next few questions ask about how you might achieve whole of person practice in your setting. I'm just wondering how do you achieve whole of person practice as an OT at [organisation name withheld]?

R: I guess as part of my training, I've sort of got the model then the theories about occupational performance and occupational roles. So I guess it's the background to the way I work with clients. It's about help to achieve that. So rather than looking at the clients and just their impairments, I'm able to look at that whole person and are directed by their occupations roles and the environment they're in. I guess I'm lucky in this setting too that my setting support staff and the frameworks that we use support staff. We don't have strict you've got to have therapy done within, I don't know, 12 week blocks or - it's very guided by where that particular person is at, which I guess allows you to look at all of those different areas rather than something that's very structured and time limited.

I: Sure. How do you see other practitioners implementing whole of person practice?

R: I guess it depends. So you've got your good examples and bad examples. I guess - I think - how would you see other practitioners. I think it's very setting dependent from my experience. So I think certain settings are more limited than others around whether that can be achieved. So whether there are restrictions on - and just availability of staff or availability of therapy and - for instance, I know we work closely with the day hospital at the [organisation name withheld]. Obviously there are certain limitations there around how a therapist can engage with someone outside of that setting. So they're seeing a client within an acute setting dealing with things outside of that setting and often that's quite difficult for clients to contextualise.

I: Sure.

R: So I think that would be difficult to do looking at that whole person and getting the exact picture of actually what's happening for that client in their community or how they're participating in their community. I think that would be very difficult to see.

I: Yes, as opposed to later on in the continuum…

R: Yes.

I: …when they are actually sort of I guess more able to participate?

R: Definitely. I guess there's other- does that come under that? I'm just thinking about - no, that comes under challenges. I'm just thinking about clients difficulties with impairments in insight and reasoning and things like that. It would be quite hard to - but that's kind of the next question.

I: You're leading…

R: So good - I've seen more good examples I would say as whole of person practice happening within the community than I have acutely.

I: Yes.

R: Because of the limitations of the setting.

I: Sure. I guess the ability for the person to be able to participate as well?

R: Yes, that's right. We deal a lot with families here. We don't just see the client as our - the person with a brain injury as our client. We're actually working with the whole family. So brain injury can often have an impact on lots of different areas of a client's life and also their carers and family situation.

I: Definitely.

R: Yes, so that transition time from hospital to home is an area that we cover, but also just every day [unclear] events that happen. So I guess we've got that flexibility that it may not necessarily have been maybe a brain injury related situation. It could be just every day life's ups and downs, but I guess our service is able to tap in and assist with those things as needed.

I: Sure. There are always challenges or difficulties in providing rehabilitation to people. I'm just wondering what external factors do you think might influence the real world delivery of whole of person practice?

R: Yes. I guess I've touched on some of those. The other things is when we're being client centred, sometimes clients have quite poor insight and reasoning and have difficulties setting goals. Just the nature of brain injury. Sometimes there's family dynamics and those sorts of external factors that make it difficult to carry out rehab. I guess some of the other things are around those things that I mentioned with day hospital when it's working on community goals but with someone in a setting that's not where they're actually going to be completing that task or participating in that community. So that's often hard for them to translate between that setting and where they actually need to maybe do that task or activity.

I: Yes. Just to clarify I've heard you correctly. Would an example of that be for example, of a client participating in therapy in the hospital and then not being able to translate that back into their home setting?

R: Yes. If they've done a - if the client's goal is around getting planning and organising and maybe learning how to use their mobile phone, for instance, as a reminder and then they get their mobile phone and we would see the client at home. They're struggling even to remember where they put their mobile phone let alone how they're using it. Or when they take a phone call they're not then putting that information into their phone or - so it's difficult if that's not happening in their home environment where they actually need to do it.

I: Yes, definitely. You mentioned earlier as well the availability of staff in therapy as a factor. I was just wondering…

R: I've worked in New South Wales as well as Queensland. There appears, depending on the areas, there's limited therapy available in Queensland I guess in comparison to other States. Also depending on where the client lives in Queensland as well, depends on whether they have, you know, going in as an outpatient to have therapy or whether they're having therapy in their own home. Then our service is only able to see people face to face within 150 ks of Brisbane, so anyone outside of that, we would try to do intervention with over the phone, which is very difficult.

I: Yes. It makes you really think about what life might be like for rural populations, doesn't it?

R: Yes, very difficult. Some of the external factors. I guess we're always weighing up the difference between client centred goals and - so sometimes clients are going through a period of transition and are actually - their goals are very impairment focused goals. I want to improve my memory. I want to improve my speech. I want to improve those sorts of things. I guess part of our role is obviously accepting those goals, but also talking to them around participating in their community and having therapy as everyday life and that transition from having therapy to engaging in normal activities.

I: I'm also just wondering what challenges you may face within your discipline as an OT?

R: In doing person [inaudible] practice? Sorry, holistic, yes.

I: Yes.

R: Challenges as an OT. I guess it's some of those things about location, travel. Like some of the more practical things, I guess. It's not just as an OT maybe. Maybe expectations at times from other professions, maybe - there might be situations where other professional, other services involved might have an expectation of us doing - we don't do technically hands on therapy here. We work more as case co-coordinators. So I guess expectations from other services that we're going to do ongoing OT therapy with people, which is funding limited.

I: Yes, I was just about to sort of clarify that, yes.

R: Yes. The way our team is set up, we work more as rehabilitation co-coordinators. We just come from different backgrounds.

I: Yes. We touched on this a little bit earlier as well. We have spoken a little bit about how whole of person practice may change across the settings. You mentioned that it could perhaps be more difficult in the acute setting as opposed to the community settings. I'm just wondering whether you believe goals for patients may change across these settings as well?

R: Yes I think they do. I think we see that in our setting. That's part of our role is to assist with that transition. So often clients I feel within acute or rehab, are thinking about more impairment based goals, so I want to improve my memory, I want to be able to walk, those sorts of things. We find when clients come to us and have been involved in - they've been at home, their goals are more around getting back to work and participating in things within the community. I want to be a parent again to my children, that sort of thing.

I: Sure.

R: I will say though I imagine like across the setting, clients sometimes find it difficult across this whole spectrum, find it difficult to link the small goals sometimes to those longer term goals. So finding it easier to have, yes, I want to return to work, but the little steps that you need to go through to be able to get to return to work is often- is sometimes difficult. I think that would probably happen across the whole spectrum.

I: Sure. I'm also just wondering if there was anything more you wanted to add about how whole of person practice may change, seeing as we touched on a little bit earlier, but not under this question?

R: Whole of practice. I guess possibly there might have - within our setting we work with clients and their families and then we assist in linking with other services. So I'd imagine that when you're working in acute or sub-acute, you're often working within multi-disciplinary teams doing your discipline specific roles with that client. So I'd imagine that that might have an impact of whole of person practice, just working within different teams as well as different settings.

I: Sure. My last question which isn't actually on the list. I'm just sort of wondering about it. When you say that you link patients into services in the community, do you find it often that there aren't many services? Like, that that's a problem that there's not many services to link in with?

R: It really depends on the client and what their goals are. So we're not just looking for disability sector services often. So we'd be linking with TAFE or with, I don't know, different leisure pursuits. I guess because of our focus around participation and community integration, it's not just looking for another rehab service or those sorts of things. But yes, it can be quite difficult. It can be difficult because of the hidden nature of - the hidden disability of brain injury. So often people - there's a lot of services for people with intellectual disability, but brain injury isn't that obvious often. So part of our role is around educating those non-disability sector services around brain injury and how best to support our clients.

I: Sure. That's all the questions that I have for you this afternoon. Is there anything else that you would like to add that we haven't spoken about today?

R: I think that's probably most of it, yes.

I: Pretty well covered?

R: Yes.

I: I will stop recording then if that's everything.

R: Thanks, yes.

**Transcription ID13:**

I: Thank you for agreeing to participate in the study examining brain injury rehabilitation. I understand that you have read the participant information sheet and have consented to complete the short interview. To remind you, our conversation will be tape recorded. However, any information you provide will remain confidential and will not be disclosed to anyone other than as part of a summary report, which means results will be aggregated and your answers will remain unidentifiable. Your participation is also voluntary and you are free to withdraw from the project at any stage. Do you have any questions?

R: [Inaudible]

I: Sorry, you might have to speak up a little bit, it's a bit hard to hear.

R: Sure. There we are.

I: Yes, that's better. Okay, to begin, can you tell me what are the guiding principles of brain injury rehabilitation within your practice as an OT?

R: Wow, that's a deep question. I guess my guiding principles are definitely from a paediatric perspective - family [unclear] practice. So I guess identifying key areas of function and so it's all about participation and engagement in the occupational roles that they want to engage in. So working with the families to identify what the problems are and then working back from there on an activity and impairment level, to work out what is it that's causing their lack of participation. Then obviously it's the environment and all those other aspects to facilitate their maximum participation.

I: That sounds really interesting. Is there an example of a recent client that you may have in mind, just to demonstrate those principles?

R: Okay - oh gosh, I haven't been in clinical work for about six months [inaudible] let's bring this forward. There was a young 11 year old boy who had a traumatic brain injury as a result of a pedestrian versus bus type accident…

I: Oh no.

R: So he - I saw him through the acute stages through to outpatient reengagement into community. At the initial stages he wasn't able to talk, he was in PTA. So I guess at that stage the key thing was surrounding communication and monitoring his cognitive status in regards to his PTA. Then once that resolved then we started working on things that he - he has a left hemi so it was working on the things around self-care.

So the occupational roles about becoming independent and being able to independently shower, dress and do those sorts of things. His left hemi was impacting that so we identified getting dressed in the morning after the shower was an issue. So we then worked on return of strength and sensation to that arm. Then worked on grading that up to how does that fit into the task? So we do some related activities about practicing dressing and all that kind of stuff to achieve that goal. Then working with the family to ensure that that was their goal that they wanted to identify, wanted to work on in the initial stages. Then I worked with them because they had to put those strategies and practices into place when we weren't there during the day.

I: Yes. Excellent. Do you believe that they are different to general rehabilitation principles?

R: Nope. They [unclear].

I: Yes, no worries. The next few questions that I have ask about your thoughts on what is whole of person practice in relation to brain injury rehabilitation. From your experience, how would you define holism?

R: I guess it's just making sure that you look at all aspects of their life, not just focus on the deficits that you see or even the obvious deficits because sometimes they can present quite obviously a left hemi or communication problem or something like that. But really to see them as a whole person that comes from a certain family background who had certain life roles that they wanted to engage in. Yes, their head injury has probably changed that but we need to investigate how it's changed the whole of their world, not just what we can physically see then and there.

I: Definitely. So just to make sure that I've understood you correctly, it's about looking at the person's pre-morbid functioning as well as their presenting functioning while taking into account problems that may not be directly evident?

R: Yes.

I: Excellent. Some people may find it challenging to define whole of person practice. I'm just wondering if you have any suggestions as to why this might be.

R: I don't know. [Laughs]

I: [Laughs]

R: Because I guess they maybe work - I work in a very cohesive multidisciplinary team so I can get perspectives from all kinds of people in regards to that whole of person picture. Whereas people that work a bit more in isolation - so not so much in a tight team - they get a little bit fixated on their own specific problem. As a physiotherapist they may get fixated a little bit on walking [inaudible] and stuff. So maybe that's one of the reasons …

I: I'm just curious that you say that. I'm just wondering how do you see other practitioners implementing whole of person practice within your team?

R: I think the culture of our organisation facilitates that, in that we are always getting together, setting joint goals and having multidisciplinary team meetings. So I think everyone's perspective is always included in that. By nature it helps you start to see the person in its entirety. I guess also the background that I have as an OT and my training [unclear] encouraged that from day one. So I think depending on your background and all that kind of stuff, I think most disciplines now have a bit more of a holistic view of the person than they used to.

I: Also really trying to collaborate and come together I guess as a team - yes. So what do you think would be the opposite of whole of person practice?

R: You're really fixating on one specific aspect and probably becoming a bit more impairment focused rather than participation.

I: Yes, definitely. Could you possibly give me an example of what that might look like?

R: I guess maybe focusing on maintaining a joint range of a person with significant spasticity but without the goal of that being functional. You maintain a good joint alignment for maintenance later so you don't get pain or something like that. But if they're not using that hand functionally being only focused on all that splinting and all that kind of stuff when they're actually wanting to achieve other goals like how do I learn to dress when I only have one arm…

I: Yes.

R: …and ignoring that goal and just focusing on the fact well, we need to be wearing a splint. We need to do this and we need to do that. You need to do your stretches and all that kind of stuff. That probably would be one example.

I: Yes. What about in terms of environmental and contextual factors?

R: What do you mean - in regards to how that impacts holistic function?

I: Yes.

R: So I guess we try really hard to do as much contextual based stuff as possible. But service restrictions - in that we can't often get out to the community and stuff like that - I think with head injury it's really important that you do contextual based therapy and that's what the research is showing. But when you work in a tertiary hospital, having the capacity to go out and assess within the school is kind of limited when you've got major caseloads and stuff. So we do it on an as needs basis but I think it would benefit the whole picture and understanding of how they're functioning within their environment if you could actually get out there as much as possible.

I: To actually see it, yes. Now this question I've been dying to ask you from a paediatric perspective. Does whole of person practice look different for children and adults in brain injury rehabilitation?

R: I think it does because I think that the child doesn't exist outside of their family, whereas sometimes adults have the capacity to exist outside their function and their family. Whereas with kids their whole capacity goes back to that whole environmental thing - is that one kid's presentation - if you just look at two children who present exactly the same - you put them in different family contexts and their presentation is completely different, given the way that they have - just in things like how they encourage levels of independence, what is the grief reaction from the family. You get that in adults too but I guess when it's an adult to a child - like the parent to a child - that protective instinct is a lot harder. They never experience also what life is like as an adult. So they have their - they need to be developing a lot more skills and they've had that injury then that's going to impact their ability to develop those skills later. Whereas the family of an adult has experienced them living independently. So they have that hope that they will get back to that stage.

I: So rehabilitation could potentially be quite different?

R: Definitely.

I: Yes - because of the environmental factors around the injured person?

R: Yes.

I: The next few questions are about how you might achieve whole of person practice in your setting. I'm just wondering how you might achieve whole of person practice as an OT at the [organisation name withheld]?

R: I guess we work together. We have our own disciplines and specific kinds of assessments and perspectives and things like that and use frames of reference that are relevant to us. But I think it's then coming back to the team and collaborating with them and identifying what are the key issues from everybody's perspective. Then I guess it's a process of reflecting to the family and making sure we're on the same page as what they are in regards to what their goals are, what the child's goals are and things like that.

I: Yes. I'm just wondering how you might - which setting you would identify with? Whether it would be acute, subacute or community?

R: Our service does all three.

I: All three, right. So you'll be able to see them all the way through?

R: Yes.

I: No worries. I just wasn't sure. I just wanted to check with you.

R: We follow them through until they finish year 12.

I: Then where do they go?

R: Well, that's what my job is at the moment - to find out where they can go.

I: Yes. So this sounds like it might touch on this question. There are always challenges or difficulties in providing rehabilitation to people. What external factors do you think might influence the real world delivery of whole of person practice?

R: I guess the constraints tertiary hospitals place on you in regards to hours, level of need, demand and things like that for service. I think that operating a community based service within a hospital also has some challenges in that they become - even though it's great that they're familiar with us and all that sort of stuff, they contextualise being out there in the community makes it harder from a hospital base. It tends to be a little bit more medical driven than what I think rehab requires. I think the medical component is very important but I think long-term a lot of the therapy and all that sort of stuff - the adjunct therapies and things like that - are important as well and that they're easier done in the community in the context that they need to be done.

I: Yes, definitely. I guess because it is a lifelong injury as well it becomes very important.

R: Yes.

I: Could you possibly provide an example when you felt that you didn't or couldn't achieve whole of person practice and why?

R: One of the kids that we've just recently transitioned, he had quite a significant traumatic brain injury and had a high level of needs at the end of his year 12 journey. He's still with high level. I guess then transitioning him into [unclear]. The parents were still wanting quite intensive therapy for him but the availability of that in the adult service, given that his injury was now three years post, was a bit tricky. So it was not only getting him some more rehab but the family's focus was more on the impairment levels. We were trying to move him to more of a - well, what's his participation level? But the parents weren't ready to go there. Then even when we did try and go there, the availability of services for respite and all that kind of other support, there's nothing really available for him.

Then it was interacting with other agencies like BSQ and other things like that to do that whole of picture support - because we can't provide respite services for these families and sometimes they need that. So it needs to have that funding package. If you don't have insurance from a head injury then there's actually very little resources in the community to support that. I get feedback a lot from GPs and all that sort of stuff that people that have what we consider minor injuries but are having the changes of behaviour and stuff like that, the stress and impact that has on the family is quite significant. There are no services then to support them in the community doing that. Our hospital doesn't support that either. We can't provide support to give the family a weekend away or something like that.

I: Definitely. I guess that really reflects back on the holistic rehabilitation. I mean, if there's nothing there to support the family, who then is supporting the injured person? Yes, it's a bit of a dilemma isn't it?

R: Yes.

I: In terms of the transition between the paediatric services to the adult services that you were talking about, I'm just wondering what the major factors that might influence the care of the person are in that sense?

R: At the moment we are relooking at the transition practices that we have between the children's services and the adults' services. I guess some of the key barriers we have identified so far is the fact that adult services are - to our knowledge - quite overburdened with their current case loads. So someone who’s had a childhood onset injury, the priority of their treatment is a little bit less than someone who's just had an acute stroke or something like that. So it's finding an adult service that will assist with the management. Also their understanding of the impact of having a head injury during key developmental stages is a little bit less in the adult sphere.

So they kind of expect - there was a myth that the earlier you had your head injury the better off you would be but actually research is now showing that when you have your injury early on it has a greater impact on your [unclear] functioning and that's going to be long-term. So paediatric head injury was prioritized lower because they thought they should be fine by now, whereas it's not like that. I guess having the time and the ability to establish the links with adult services, given that we don't actually have a high flow through to the adult services. I guess connections - yes, in small degrees is back to adult services [unclear] management and stuff like that. But really it's about the connection back to the GPs and also a lot of non-medical type services. So these guys need employment agencies and things like that that really understand their history, as opposed to needing significant ongoing therapy. They're the kind of key people that we're transitioning to. So we're reviewing processes and trying to make sure that we're doing that well and getting that sorted. But it's a bit of a work in progress at the moment.

I: Yes. You've raised some very significant issues with that, especially just underlying it all with the education and, like you said, people not really understanding the impact that an injury can have on a young person. Yes, very interesting. So rehabilitation goals for patients may change across settings. Firstly, do you think this is the case?

R: Definitely.

I: Yes. Could you elaborate on that one?

R: You get a lot of changes in their initial acute phases and so you're working with that. Then as it gets towards the end of their acute phase it starts going okay, what are the key issues for the next phase of integration back into the community? We do a lot of school liaison and getting them connected back into the school. So that's very different to the acute management of let's get you from day to day activity, let's get you eating and swallowing and dealing with basic bodily functions. Then as we move to less acute it becomes more about how do we get you back into your world and into the occupational role that you want to establish and want to maintain.

Then after that again it's how do you learn to self-manage your condition and [unclear] learn to self-applicate and deal with the residue effects and having - we change your environment and remediate the kinds of things … to have maximum participation in their life. As their life goals change as they get developmentally older and they have different expectations placed on them by society, you have to re-evaluate. Well, what's the goal now? The goal is to maintain your participation at a developmentally appropriate level as much possible. It's those kind of things.

I: That was an excellent example and you've just answered by next question. [Laugh]

R: [Laugh]

I: So I was just wondering, is there anything else that you'd like to add that we haven't talked about today?

R: No, I think that's all.

I: We've got it covered?

R: Yes.

I: All right. I'll stop recording now then.

**Transcription ID14:**

I: Thank you for agreeing to participate in the study examining brain injury rehabilitation. I understand you have read the participant information sheet and have consented to complete a short interview. To remind you, our conversation will be tape recorded. However, any information you provide will remain confidential and will not be disclosed to anyone other than as part of a summary report, which means results will be aggregated and answers will remain unidentifiable. Your participation is also voluntary and you are free to withdraw from the project at any stage. Do you have any questions?

R: No.

I: Okay. Firstly, can you tell me what are the guiding principles of brain injury rehabilitation within your practice?

R: I work for - the funders that support me, the CPP scheme - so the guidelines are very much set by the MAIC industry. So there are industry standards that have to be upheld. So basically the principles are related to those which include providing services in a timely manner, assisting the client to return to pre-injury status, providing consistent and ongoing support and liaising and communicating with other key stakeholders.

I: Can I just get you to elaborate a little bit on MAIC?

R: The Motor Accident Insurance Commission.

I: Thank you. I'm just wondering whether the principles you've just mentioned differ to the general rehabilitation principles?

R: I think that they do in some sense because you are very much - what you're allowed to provide is very much guided by the funding body. So it's different to a rehabilitation setting where all the needs are recognised and that you're allowed to provide all the support that's required rather than being restricted by the support that is considered to be reasonable and appropriate.

I: Yes, I understand what you're saying. The next few questions ask about your thoughts on what is whole of person practice in brain injury rehabilitation. From your experience, how would you describe holism?

R: Holism is addressing all the person's needs. So it includes their physical, social, cognitive and emotional needs.

I: Could you elaborate using perhaps an example - a recent example?

R: Of where this has been provided? A holistic approach?

I: Yes. Towards a patient if possible.

R: There's a patient that I work with that has - he had a severe TBI - traumatic brain injury. He remains in PTA, in post-traumatic amnesia. So he has never emerged. He will not be able to go to work so it's incredibly important that a holistic approach is considered for him. This includes looking at - he has no day to day memory function. So it's really important that his medical needs are considered and explored because he's unable to report on those. Within that he has very limited insight, so his emotional needs are also really important to be considered because he can't really express how he's feeling about what's happening to him and there's confusion. He doesn't really understand what's going on. So emotional needs are also really important there. Physically he has reduced balance skills so he is at a high falls risk so that needs to be considered, as well as - because he remains in PTA, it's really important to make sure that he does still engage in purposeful activity. So looking at - that's where you get your really holistic approach. It's considering all of those elements and then drawing them into activities that are purposeful to him.

I: What a beautiful example. I'm wondering, why might it be challenging for some people to define whole of person practice?

R: I think when they're restricted by the funding body. So even though as practitioners we recognise how important it is, some of the funding bodies now are shifting away from, I guess, acknowledging the importance of those purposeful activities. Although they will allow you to work within the home, they are not allowing some community involvement - community involvement is just as important. An example of that is Lifetime Care will no longer consider any vocational activities. So for somebody that cannot return to work, it has severe implications.

I: Yes, it does. In that case, what do you think would be the opposite of whole of person practice?

R: Where you're just targeting specific - it could just be specific goals identified by the client that don't - where you're being required to provide a client centred or client focused practice, which is really important. But sometimes because of some of the ABI sequelae - so some of the things like they might not have insight into other areas that are really important as well. So your practice could be not holistic at all.

I: Just to make sure that I've understood you correctly, it's more in a way where you're focusing more on the injury or the goals that the client may have and not taking into account those environmental factors?

R: Environmental and social - it could even be medical or other physical issues as well. But if they're not recognised by the client - for example I've got somebody else with a traumatic brain injury who also has no insight into her condition and she has reduced balance. She has safety issues - she's very impulsive - but because they're not goals for her because of her reduced insight, if I was to purely focus on client centred practice then other very significant factors would not be considered.

I: Yes, I understand. I'm wondering as well, does whole of person practice look different for children and adults in brain injury rehabilitation?

R: I think that whole of person practice can be because often the child is represented by the parents and so their needs are often conveyed to the parents - sorry, conveyed to the therapist working in ABI - by the parents. So the parent may not recognise the whole of person issues. They tend to - often when you're working with kids in the community, the environment in which they're living in can be very - it can be a very stressed environment where the family are a little bit disorganized because they're completely overwhelmed by what they're dealing with - particularly with an acquired brain injury. So what happens is that instead of being proactive they tend to be reactive.

I: Yes. I guess sometimes you would see sort of cotton-wooling the child - trying to overprotect them, I guess, within the rehabilitation?

R: Yes, that's very true, particularly if there's some guilt associated to the injury. That happens a lot with a lot of the parents, that they - yes, that there is because it's an acquired injury and they carry the guilt, particularly if they were the driver of the vehicle. We've got one young girl where the mum - the little girl ran across the road and the mum couldn't do anything about it but saw it. That guilt…

I: The poor thing.

R: …stops her from allowing this young girl to really develop independence. So definitely.

I: Yes. Are you involved in the transition between the paediatric services and the adult services?

R: I have quite a few teenagers that I work with - so yes.

I: How do you feel whole of person practice changes with that transition?

R: Actually, I wouldn't be able to comment on that yet because they're all low teens and they haven't got to the point where they're transitioning into adulthood - so probably not actually. But it'll be something that I'll deal with in the next couple of years.

I: Yes - as you follow them through?

R: Yes.

I: Yes, that's no worries. The next few questions are about how you might achieve whole of person practice in your setting. I know you've touched on it a little bit but I'm just wondering if there is anything else you'd like to elaborate? How do you achieve whole of person practice as an OT in private practice?

R: It's really difficult actually. It - because in private practice you're relying on funding bodies to fund what you do and you have to stick so stringently to their requirements and their framework - because otherwise you don't get repeat referrals. So it's very important to stick within what they will fund. Because of that it is very hard. There are often needs that you know are there but you can't actually address them because the insurer won't see them as reasonable and appropriate or related to the accident specifically, even though they're probably secondary issues that definitely need addressing.

I: My goodness. What happens in that case if needs aren't being addressed? Are you able to refer out or what happens there?

R: You basically advise the family to seek other services and you can inform them and educate them. But they have a significant impact on what you're trying to achieve. So things like there's a marriage breakup - which is very common after a child suffers a traumatic injury where the family are living with trying to deal with the disability and particularly if there's guilt associated. The breakup - which is secondary to the rehabilitation that's been provided but very much impacts significantly on how that person copes with the rehab - you're not allowed to address it because it's a social issue. So because it's a social issue where it's secondary to the motor vehicle accident, the insurer doesn't see it as their…

I: As important.

R: …concern. There's a little boy that I'm working with at the moment and he lays awake all night long worrying that he's the reason, because of his accident, that his parents are split up…

I: Poor thing.

R: So he is not coping at school. He experiences increased pain because pain's often associated with depression. He - yes. It just goes on and on and on - the impact really of the injury on the social situation but because it's not a primary link it's not…

I: It's not covered.

R: …something we can touch.

I: Wow. I'm wondering as well how you might see other practitioners implementing whole of person practice?

R: It's hard for me to comment on that one because I'm…

I: Yes, I thought it might be.

R: …a sole practitioner.

I: No worries. Also, what external factors do you think might influence the real world delivery of whole of person practice? I know you've mentioned the funding bodies. I'm just wondering if there's anything more.

R: I guess some other factors would just be how the different stakeholders work together. For instance, I work quite closely with the hospital. It just depends on how because the hospital - particularly for children, the [organisation name withheld] provides six monthly monitoring of the kids with an acquired brain injury. So I am - sometimes I'm invited to those reviews so that I can then follow up on the issues that are raised. But there are other times that I'm not. It just really depends on the openness and willingness of public institutions to work with private practitioners. I think that that has a really significant impact too.

I: Yes, it definitely would. I'm not too sure - from what I've gathered you work with the patients along their continuum - from time of injury through to…

R: That's right. It can be life. I have one client where - this is a young girl and her case is closed but because her funds will be - she'll require case management for the rest of her life. So I've now been engaged, not through the insurer, but through the Public Trust to provide that on an ongoing basis. So it is, it's a very long-term relationship with many clients.

R: Yes. So how exactly might whole of person practice change along the continuum?

I: I think that that whole of person practice changes according to - it's really the access that you have to the client and the services. So if - I've mentioned a lot the funding body - but while they've got a claim open then it's very claim driven. When the claim closes then often the funds are managed by public trusts and you have a lot more freedom to be able to deliver a whole of person practice then. So it really just depends. Then when the client themselves become able to make decisions about their care, it will be very much reliant on the person reporting to you what they need and having access to them to be able to identify what else is required. So it really depends on access - access to the client, access to their home situation, school, work environment and the different reasons why that access might not be provided. Sometimes it might be the client not allowing you in and not giving you the information you require. Other times it's the framework that you're working within.

R: Yes. That's so interesting that you say that because, like you said, every injury is unique and each person is going to be in a different situation.

I: Yes.

R: That's all the questions that I have for you. Is there anything else that you would like to add that we haven't talked about today?

I: No, I think that that pretty much covers it.

R: It pretty much covers everything?

I: Yes.

R: All right. I'll stop recording if that's the case.

**Transcription ID15:**

I: Thank you for agreeing to participate in this study examining brain injury rehabilitation. I understand you have read the participant information sheet and have consented to complete a short interview. To remind you, our conversation will be tape recorded, however any information you provide will remain confidential and will not be disclosed to anyone other than as part of a summary report, which means the results will be aggregated and your answers will remain non-identifiable. Your participation is also voluntary and you are free to withdraw from the project at any stage. Do you have asny questions?

R: No.

I: Okay. So my first question is just regarding the brain injury principles and I'm just wondering if you could please tell me what are the guiding principles of brain injury rehabilitation within your practice as an OT?

R: So I guess this - when I saw this question I was like this is really tricky to answer. I guess from an OT's perspective, we would always use something like the Occupational Performance Model to guide whatever we do. So that's looking at the person within the context of their environment and their occupation. That would really just guide anything that we would do, regardless of, I guess, whether it's brain injury or whether it's cystic fibrosis or whether it's a hand injury or something. So that's sort of what's guiding us. Within that, we can look at the more medical aspects of the person; so their physical injuries. So a brain injury would come through that way. We also take into account the developmental stages of a child. So I guess that's a principle that we're really looking at and making sure that what we're expecting of a child is appropriate at their developmental level and what to expect a child to be able to do at that developmental level. I guess within that Occupational Performance Model, development comes into it as well and also just being family centred and goal orientated, so like the family is a huge part of that child's functioning as well; so we look at that aspect - yeah. Is that…

I: Yes. No, that's very informative. It's interesting that you mentioned that the Occupational Performance Model sort of guides your practice as an OT. I guess that ties in with my next question. I am wondering whether brain injury rehabilitation principles differ to general rehabilitation principles.

R: Generally they wouldn't, I guess. I guess brain injury and so cognition and the impact that that has on a child's function would play a larger part when we're looking specifically at brain injury. So, for example, if the child had a spinal cord injury, you know that kind of thing would play a larger part or a larger focus on what we're doing. So it's probably more not the over-arching models or ways that we're approaching our thinking. It's probably more the tool that we would then use within that - like that under - it's really hard to explain. So the tool - so if we're looking at a child's tone or looking at assessing a child's PTA or using like a Bobath approach to like neurodevelopmental treatment. So the tools, I guess, change when we're looking at brain injury, but probably not the model that we use.

I: Sure. No, that makes complete sense, yeah. The next few questions ask about your thoughts on what is whole-of-person practice in paediatric brain injury rehabilitation. From your experience, how would you define holistic practice in paediatric brain injury rehab?

R: I guess we would look at holistic practice - how would I define it - it would be - you defined it really well; whole-of-person approach. So I guess we'd be looking at not just the child in hospital with the medical condition, but looking more at their developmental level, looking at them in context of their family and their community and looking at their interests and what sort of drives them. I guess looking at it as a lifespan approach as well; so not just the child at this point in time, but looking at how the brain injury will affect them within their lifetime.

I: Yes. It's interesting to note that some people do find it difficult to describe and to define whole-of-person practice. I'm just wondering your thoughts as to why this might be.

R: I guess it depends - I think I'm a bit biased because I'm an OT, but I think the way that we learn at university and the way that we are taught is very holistic anyway. So right from the outset, the way that we are taught to approach things is very holistic, whereas maybe other professions don't get that training from that early stage and don't have that way of approaching a person.

I: Yes. So…

R: I mean from the outset.

I: So sometimes going through a university degree and learning in that environment may be different once practitioners enter the real world of their practice?

R: Yeah, and also maybe at that actual university level it's not drilled into them as much or - yeah, I'm not sure.

I: Hmm. It's very interesting.

R: Yeah.

I: What do you think would be the opposite of whole-of-person practice?

R: A biomedical approach. Yeah, I would say a biomedical approach. Just looking at the person and their illness and not really seeing that in context, I guess.

I: Yes. I'm also wondering your thoughts on how you think whole of practice might change from working with a child to working with an adult.

R: Maybe with a child - yeah, you've definitely got to take that lifespan approach. I guess it's hard to do that with an adult, but maybe with a child you're taking into account developmental stages and developmental milestones and also children - like you've actually got to teach them new skills, new learning, as opposed to just relearning skills. I guess for a child whole-of-person maybe takes into account family a lot - not - I guess the families are the caregivers a lot more. Hmm.

I: It's a bit of a tricky one.

R: A tricky one. Change across - between paeds and adults. I guess their occupations are different, so - I guess it doesn’t really - I guess you're dealing with different occupations, so you're dealing with schools as opposed to workplaces or things like that. So you're still probably taking that whole-of-person approach. I guess the context and the occupations might just be different and the family role might just be a little bit different.

I: Yeah.

R: Because you're sort of still looking at it as a whole-of-person approach.

I: So my last question for this section is how exactly might whole-of-person practice change across practitioners and settings? So your answer can be as broad or as specific as you like.

R: I guess across settings I think acute is very difficult. I think being in an acute hospital is very medical. Children are very acute and still often are quite unstable in the extremely acute setting. So I think it's quite focused on their medical issues and may not be as whole-of-person. Community, I think is - well, sub-acute I think we can be a little bit more - I guess that's where I'd probably put [organisation name withheld], in that acute to sub-acute section, and I guess we can be a little bit more whole-of-person. So I guess that's also incorporating a multidisciplinary team and each of us can really have a snapshot of that whole of person and all together we come up with, I think, quite a holistic approach. But I guess the gold standard would probably be more from the community side of things I would say, because you can really be within that person's environment. You can be at home, you can provide services in the home and really see that person within their environment and really understand the whole person.

I: Can I just clarify and confirm what you're saying? From that, I feel like your perspective it might be more difficult to achieve whole-of-person practice in an acute setting, but this sort of becomes a bit easier as they continue through the trajectory?

R: Yes, yeah.

I: As they get through - back into their more familiar environment?

R: Yeah, yeah.

I: Do you feel that whole-of-person practice may be able to be improved in an acute setting to equal that of community?

R: I would say probably not totally, because I think like we can't really do much in the person's home. I mean we're definitely looking at getting that child home and doing home visits, but I think in that acute setting it's really quite artificial. I mean you're providing therapy in a hospital setting. There are definitely things that we - oh, I guess that's the next section, isn't it? How do you achieve [laughter] - I'll talk about that. There are definitely things that we can do to make it more like…

I: A home.

R: …whole person, but I think particularly - because I work in the intensive care unit and the wards, particularly in that really acute setting it's medical dominance.

I: Yes.

R: Medically dominated. I think it's slowly improving to where OTs have a little bit more presence in [the acute ward] and I guess like present in the acute hospital setting, but I'd still say it's quite medically…

I: Medically dominated?

R: Yeah.

I: Sure. The next few questions are about how you might achieve whole-of-person practice in your discipline and setting. So firstly I was wondering how do you achieve whole-of-person practice as an OT.

R: Definitely by working in a multidisciplinary team and making sure that we've got the perspectives of social work, of neuropsych, of physio and speech, because I think not one person can do it all, and being open to what the other disciplines have to offer. I think by being family centred and making sure that the family enters what you're doing and that they're really guiding what they want as well; what the child and the family want by doing goal setting. I think just from using our models of thinking; by using the Occupational Performance Model. I think that gives a really nice holistic view of the child in the context of their environment, whether it's social environment, the cultural environment, the actual hospital environment, plus their occupation. Yeah, I think that…

I: What external factors do you think might influence the real world delivery of whole-of-person practice?

R: Time and staffing. When you're short staffed I think you can't do everything that you would love to be able to do. Lack of the - so the same thing would be for community services as well, I think. There's a real lack of community services for us to refer on to and lack of staffing in those centres, so ideally what you'd like to be able to offer the child is hardly ever…

I: Available?

R: …available.

I: Yeah.

R: So I know they have exactly the same issues that we do. The same with presence in school; the schooling system as well. There's really a lack of support staff for children with brain injuries to reintegrate back into the schooling system. So if there was like an advisory visiting teacher specifically for brain injury, that would just be brilliant. I think the medical model that we have in the hospital…

I: Just the dominance of a medical model?

R: Yeah, yeah, and funding [laughter]. Because, yeah, there's limited follow up that we can offer. I think from our point of view as well, like we're only a short-term rehab service, so [organisation name withheld] is really the only service that can provide that long-term follow up brain injury specific, whereas we've only got funding to do that for a year for each child, so if we had more funding that would be amazing.

I: Yes. From what I've heard, I've heard a lot of sort of systemic influences and just on some things that you've mentioned earlier, just about how each - that it is a multidisciplinary approach and it sounds like each discipline may have their own tools, I'm just wondering if there are any challenges in how each discipline comes together and how they use their individual tools to promote whole-of-person practice.

R: Sometimes I think from an OT point of view, because we can be quite holistic, we do cross over into some other specific disciplines. So I know physio and OT cross over quite a lot. I mean there have been difficulties with OTs and physios and understanding each other's roles and who does what, but I think, yeah, that has been a challenge, but I think open communication is the key to that; making sure we're really working on the same goals as well. I've heard recently - actually yesterday, that apparently OTs are the main threat to social workers. I've never heard that before, but, you know, you hear new things all of the time. I didn't think we were, but I think, yeah, communication is the key.

I: Yes. My final question has just come up, just listening to speak. In the literature it suggested that for children with a brain injury, time for the injury indicators to appear as well as important transition phases may be an area of interest or, you know, that may provide challenges. I was just wondering your thoughts from a professional's perspective.

R: Yeah, definitely. Yeah, we would say, particularly from a developmental point of view, you're not going to know the full extent of a brain injury on a two-year-old until they really do get through school and have more of an academic challenge and - yeah, the whole of their brain and development is challenged, you're just not going to know that. Then those transition phases from two - actually going to school and then transitioning from primary school to high school and becoming a teenager and all of those things. So we just don't have the capacity to follow kids for that long, so we definitely draw on [organisation name withheld] as our resource to make sure that they can continue to monitor those kids.

I: It sounds like the referral processes are extremely important in that regard?

R: Yeah, definitely, yeah.

I: Perfect. Well, that's all of the question that I have. Is there anything that you would like to add that we haven't spoken about today?

R: No.

I: Is that everything?

R: That covers it, yes.

I: Okay. I'll just turn the recording off.

**Transcription ID16:**

I: Thank you for agreeing to participate in this study examining brain injury rehabilitation. I understand you have read the participant information sheet and have consented to complete a short interview. To remind you, our conversation will be tape recorded, however any information you provide will remain confidential, and will not be disclosed to anyone other than as part of a summary report. Which means results will be aggregated and your answers will remain non‑identifiable. Your participation is also voluntary and you are free to withdraw from the project at any stage. Do you have any questions?

R: No, that's all good.

I: Firstly, can you tell me, what are the guiding principles of brain injury rehabilitation within your practice as a music therapist?

R: I guess the overriding principle for all brain injury rehab - for us - is about rhythmic timing, and a particular principle called rhythmic entrainment, which is about using an external rhythmic cueing to cue movement or speech for a person with a brain injury. Yeah, that's probably the main one. I guess the other principles that apply across the board - but particularly as well, in brain injury rehab - would be things like client-centred practice. So that would be - or patient-directed programs, and then for me, working with kids, it's more about family-centred practice.

I: Sure. Are brain injury rehabilitation principles different to general rehabilitation principles?

R: Yeah. I'm trying to think of how to explain how they are. I guess - that's a tricky question this early in the morning.

[Laughter]

R: They are, and I guess primarily we are looking at things like neuroplasticity. Trying to find either compensatory strategies, or restorative strategies, that are more to do with brain training. So, either creating neural pathways in different ways, and for us, using music as a way to do that. Sometimes the musical neural pathways might be a little bit more unique, as opposed to the traditional way that they might have done a particular function or skill previously.

I: Sure. I'm just wondering as well, if you could please tell me a little bit about your discipline as a music therapist?

R: Yeah. The best way to explain music therapy is that it is an allied health profession. But, rather than a particular area of function that we're working on, it's more that we're - it's more defined by the tool that we use, which is music. That could be listening to music, playing instruments, singing, and the reasons for doing those particular interventions depends on the goal of the patient. We might work really closely in with the other allied health professionals. The physios, OTs, speechies, and work alongside with their goals, but using musical strategies to meet the particular area of function for the patient.

I: Sure. That explains it well. The next few questions ask about your thoughts on, what is whole of person practice in paediatric brain injury rehabilitation. From your experience, how would you define holistic practice?

R: I think there are two parts to holistic practice in paediatrics. The first would be about the whole of the child, and the second is about the child within the family system, because that is such a big part of their life when they're younger. So, I guess the whole of child is just looking at: what's their history? What are their skills? Pre-morbidly, particularly. What are their interests? Then looking at the family system as a bit of a bigger picture, just in terms of: what are the other relationships within the family? How does the family function? What are their general coping styles? That sort of thing.

I: What do you think would be the opposite of whole of person practice?

R: I guess that would be more looking at a particular area of function for example, but in isolation. So, if we were just focusing on getting them up and walking, and disregarding thing like, whether - how much the parents wanted to be involved for example, or what was actually going to motivate them to get walking. So, whether it's using things like talking about getting back to school, or getting back to their favourite sport, or something like that. If we disregarded all of those things, I guess that would be, yes, the opposite.

I: Family appears to be an important theme for paediatric brain injury rehabilitation as you've mentioned. I'm just wondering how practitioners actively involve families in their conceptualisation, or approach to rehabilitation?

R: I think it's a tricky thing to do sometimes. I guess, ideally, it would be about discussing with the family, first of all, what their goals for the child would be. Obviously, that's going to change along the road of rehab, but making sure that whatever we're doing is actually tying into what the family sees as priorities for the child. Then working out ways to help to teach the family members things like the exercise programs, or just giving them the skills and the knowledge, to actually be the ones doing the most amount of the intervention with the child.

I: Yes, because I can imagine it would be so important for the families to be involved in the rehabilitation program.

R: Definitely.

I: I'm also wondering - I can imagine the reaction the families might have when their son or daughter experiences a brain injury, and I'm wondering what happens when you come up to resistance? When you come up against resistance from the family?

R: I guess when that happens, it usually has a lot to do with the, as you say, the - how the family's feeling in terms of, do they have a - are they still experiencing shock and grief and loss at the impact of this injury? Then I guess it would be really important to give the family members as well the opportunity to process and work through that. So that might be the role of the social workers or even the neuropsychologist to give more information about this is the particular impacts of the brain injury, in these areas. So I think sometimes that grief and loss could be the cause of the resistance, and when it does happen, I guess it's important for health practitioners to be really, just aware and mindful that this is a really traumatic experience for the whole family to go through. Perhaps if it is important to go a little bit slower, or a slightly different direction with the therapy, that's actually going to be more beneficial in the long run than pushing, the rehab agenda onto the family.

I: It sounds like education is very important, but in terms of a brain injury, a lot remains unknown. I can imagine it would be very difficult.

R: No-one can really predict exactly what the outcome's going to be for their child, which I think would be a scary thing for a family.

I: Absolutely. How exactly might whole of person practice change across practitioners and settings?

R: This is a tricky question too, I think.

[Laughter]

R: I guess - you've listed there some acute, sub-acute community, and I think it definitely does change. Perhaps it changes as well, as the child particularly is progressing through their rehab. They would - the focus of the rehab would go from very small gains just for maybe - just for the child, if that makes sense. So, just for a particular area of the functioning, to then as they move into the community, it's more about - how do they interact with the community? How do they access just general day-to-day things? So, I guess it's almost like the focus goes from a very small focus of just the child and their particular functioning, and then gradually, looking at the family system. Then looking at the whole community system, including school, and any hobbies and things that they have, and that becomes the whole of person - if that makes sense - as they progress.

I: Yeah, that does make sense. I'm just wondering, from your explanation, whether you believe the person's environment has - plays into that?

R: Oh, definitely. You see that really clearly when they're in hospital. It's just a foreign environment [laughs] I think, it impacts on their rehab. Which is why I guess as much as possible we try to get them either out at hospital accommodation with their family, or at home as soon as they are safe to be there. Just to try to normalise that as much as possible for them.

I: The next few questions are about how you might achieve whole of person practice in your discipline and setting. Firstly, I'm wondering how you achieve whole of person practice in your discipline as a music therapist?

R: I think one of the beneficial things about music therapy is that we're not necessarily focussing on any one area of functioning. So, whereas a physio might be thinking mobility is a primary goal. For a music therapist, the initial goal really, is to build rapport and see how the patient is going to respond to music. So there's not a lot of - I don’t necessarily go in with an agenda of well, this is how they're going to respond. A lot of it comes from them. So that's where that patient or child-centred practice comes into play, and I guess when it - when the goals are coming from the child, they’re going to be a little bit more rounded, rather than one targeted, specific area.

I: How do you achieve whole of person practice in your setting? We've mentioned, a hospital setting verse a community setting, and about involving the families. I'm just wondering how you guys go about implementing that in the hospital?

R: I guess the most important thing is having really regular meetings, and having a lot of communication between team members. So, it's almost like we're all standing on different sides of this child, and seeing a slightly different picture of them, and the family too. So, if we can get together as often as possible and discuss all those different angles, we start to see the whole picture, as a whole team. Then if we can do that, and have that communication and collaboration, we can actually start to all work from a whole of child perspective, and use a more trans-disciplinary approach, where we are all working on the same sorts of things, but just in slightly different ways.

I: What external factors do you think might influence the real world delivery of whole person practice?

R: I think probably the most challenging thing is having the time to meet and discuss, and like I said, that's probably the most important thing. But it gets so busy, and actually taking the time out is really difficult, and health services are so stretched. So, I think the time constraints would be the most difficult factor. Actually, that's really the [laughs] the main one I can think of.

I: It sounds like the time will have a flow on effect in other areas.

R: Definitely, yeah. Absolutely. Though, I think as soon as you feel like you're pushed for time, you're not necessarily going to take the time to discuss the whole family system, or what everybody's perspective of this child is, which yeah…

I: Is fundamental, isn’t it.

R: Yeah, and it takes away from their rehab, and their experience as well.

I: Do you - do you see any challenges in working within a trans-disciplinary team?

R: I think the only potential challenge is misunderstanding of other people's roles. So, I think - and that just comes back to a lot of education, and really clear communication. The other challenges - so, not only do we need to sit down and discuss how the child is progressing, but actually try and collaborate and set goals together, so that we're all on the same page. Again, that's a time factor as well, that we need to actually set aside the time to do that. In my experience, the teams that I've worked in have been really open to that, and for the most part, I probably - the most functioning multi-disciplinary teams, I think, would be in paediatric rehab.

I: I wonder why that is? I wonder why paediatrics is somehow a bit easier to fit everything together, in terms of paediatric verse adult…

R: Yeah, no I'm not sure. I'm not sure why that is.

I: That's all the questions that I have for you this morning. Is there anything that you would like to add, that we haven’t spoken about today?

R: Nothing that I can think of.

I: No? Okay. I'll just hit stop.

R: Okay.

**Transcription ID17:**

I: Okay so thank you for agreeing to participate in the study examining brain injury rehabilitation. I understand you have read the participant information sheet and have consented to complete a short interview. To remind you our conversation will be tape recorded, however any information you provide will remain confidential and will not be disclosed to anyone other than as part of a summary report. Which means results will be aggregated and answers will remain unidentifiable. Your participation is also voluntary and you are free to withdraw from the project at any stage. Do you have any questions?

R: No

I: Okay. So the first section is just regarding brain injury principles and I'm just wondering if you can tell me what are the guiding principles of brain injury rehab within your practice as an OT?

R: Yes sure. I think probably a lot of regarding principles for general OT terms of reference. So things like the PEO and the occupational therapy models that really give a kind of good idea of function and the way that that relates to the way that your kind of body mechanics relate to activities that you can participate in. How the environment and other influences kind of impact on that. So I think a lot of that is really applicable to brain injury as well.

I: Sure. Sorry can I just clarify what PEO is?

R: Yeah I was trying to think as I said that.

I: [Laughter].

R: So it's the Physical Environment and Occupation Model. So it has like circles that kind of overlap that each represent. So physical parts of the body, the environment and then occupation that occurs in that overlap.

I: Sure. Are brain injury rehabilitation principles different to general rehab principles in your view?

R: I think there's probably some specific areas that are a bit different. I think the general rehab principles kind of sit alongside but there's a lot, I guess perhaps less capacity to achieve independence in some areas and that more emphasis on cognitive function and the impact of cognitive deficits on function than, I think, the general rehab population.

I: Yes sure. The next few questions ask about your thoughts on what is whole of person practice in paediatric brain injury rehab. From your experience how would you define holistic practice in paediatric brain injury rehabilitation?

R: Yeah so I think a family centred team approach is probably really important in terms of providing holistic practice. I think - I mean that's not really a definition is it? But that holistic practice is about providing family centred care for the person. So care that fits into their priorities, their needs and also addresses all of their needs and the person as a whole rather than just looking at them as a hemiplegic or looking at them as [unclear] but looking at the whole picture.

I: Yes and I guess with the family centeredness as well, especially for children it becomes very important.

R: Yes.

I: Some people might find it challenging to define whole of person practice, I'm just wondering your thoughts on why this might be.

R: That's a tricky question [laughter]. I think in some ways it's because it's part of what we do as a practice. So I think we like to think that we're doing holistic practice anyway. But I think it's that actually defining it's trickier and maybe it's because it changes in each situation and each individual and each kind of - depending on where you're working.

I: The discipline.

R: Yeah or I mean like physically what setting you're in.

I: Yes.

R: Yeah. Hard isn’t it?

I: It is.

R: I think - yeah.

I: Well I'll just ask you the next one. What do you think would be the opposite of whole of person practice?

R: Well I think the opposite would be so not being flexible or individualised in your practice. So being more rigid I guess and being very focused on the - like I've said bottom up. Like you're bi-mechanical and very focused on the underlying condition of the child rather than the whole context that they fit into.

I: Yes. So that would include the family and the school, those sorts of environment factors?

R: Yeah.

I: Yeah.

R: So not considering that I think would be [unclear].

I: Sure. How exactly might whole person practice change across practitioners and settings?

R: Yeah well I think, like I was saying with the bottom up kind of approach, I think that there are some situations that you really have to look at the person as - like look at their deficits first. So in the acute setting I think we really do need to hone in on that and look at that because it's part of their assessment and that allows us to provide their right care. Sometimes when families are grieving and it's all very acute, we need to be a bit more direct in what treatment looks like. Because that family, I guess, is in a position to be able to make decisions or don’t necessarily have the whole picture and you still need to work with them. But you need to, I guess sometimes provide a lot of that education so I think it looks a lot different to a family that's been living with a condition for a long time, then they are able to kind of dip in and out of services and direct it.

I: Direct their care.

R: Yeah. So I think it kind of - as you go on, there becomes more of that emphasis on the individual's function and participation in their community and them kind of seeking input.

I: Yes. The next few questions are about how you might achieve whole of person practice in your discipline and setting. Firstly I'm wondering how do you achieve whole person practice as an OT?

R: Yeah so I think - I mean I think it's quite well in OT because we do look across a number of areas with the child. But I think - so using those tools and using goal setting and I guess proforma to ensure that we're looking across the area, so across self-care, productivity, leisure which we often forget about [laughter] and making sure that we're using all of those. So I guess kind of our rapport proforma and our resources help us to do that. But also using tools like the Canadian Occupational Performance measure that kind of guides interviewing in that area and allows a family to have an input into what they want to address using goal settings.

I: Sure. So as an OT then that's fairly well defined as well. So I'm just wondering how you achieve whole of person practice in your setting because there's a lot of multidisciplinary team work involved, is that right?

R: Yeah. So I guess we have like regular team meetings and also a lot of communication that happens in between the meetings. Also being planned, so doing joint sessions with other disciplines where that's appropriate or where there's a goal that works for the child. I guess that just allows us to be consistent with the communication with the family so that there's no extra burden on them to kind of work out what's going on and minimise time. So be more effective in their use of time in coming to the hospital or coming off the ward or whatever it is, by working together. I think we also try to have family meetings as a team for that reason.

So we're a kind of setting where the family can raise their issues and the whole team is there to be able to direct it to the appropriate person rather than them just being said oh you'll have to talk to OT about that and wait until they see you or track you down. I think probably even our use of timetables that we use, weekly timetables so the family know who's coming which helps them to be able to fit into the hospital setting.

I: Yes. It sounds very collaborative and trying to sort of minimise miscommunication.

R: Ideally yeah, I think if all of those things can happen I think it helps. I guess the other thing with the goals it might be partly within OT providing opportunities for the family to get off the ward and do therapy in different environments as well. As part of their rehab but also that can make it more relevant or more real....

I: Absolutely.

R: ...for that family.

I: What external factors do you think might influence the real world delivery of whole of person practice?

R: I think time is the huge one. So practitioner's time to even make sure that you're sitting down discussing goals, explaining everything well to the family, when in reality if we're short of time we just have to run in, assess and run out. So I think that that can be challenging and also balancing the time that other disciplines need to see that child, with that child's need for rest and recovery. The family's time commitments as well, like if they need to be out of the hospital or doing other things. So I think definitely time is the big one.

I: Yes.

R: Also I guess resources, so just having the most up to date therapy resources and knowledge. So being able to attend courses and things like that to be able to have the best kind of evidence based practice. Yeah and I think also that team dynamic, that time, that kind of ability to trust other team members to do things or to be clear about roles and all that communication. Which often works really well but sometimes it can fall apart and I think that can make it challenging for families.

I: Definitely. Just thinking, listening to you speak and especially about time, the paediatric literature suggests that for children with a brain injury time is also a big issue as in an injury indicator. For children it can take over a period of time for those injury indicators to appear.

R: Yeah.

I: Would you agree with that statement?

R: Yeah I think so. I mean I guess it depends - yeah without knowing exactly what literature and what timeframe but definitely I think there's a lot of different reasons that someone can appear more functional than you think. Or as they develop and as they are meant to be acquiring these skills, if they fail to acquire those new skills it gives us information that perhaps their brain injury is more significant than was first thought. I think also a lot of that is us - sometimes I think, especially in the medical world, it's reliant on family's reports and family identifying concerns than - if they're not necessarily tuning into really small bits of information that there's no reason that they would be able to identify that that was due to a brain injury. Like if they don’t report it on that one day, then sometimes, yeah it does go...

I: Unrecognised.

R: ...aside, yeah.

I: Yeah because I guess for you guys being in the hospital there's only a certain amount of time that you can follow up with these children.

R: Yeah and I think - I mean that's probably one of the big barriers to external factors, is that pressure on - it's happened to us just this week, like the - so being able to keep families in for as long as we feel is maximising their recovery. But the ward who have a push for beds and that child is stable and medically able to go home so it's sometimes really hard to fight that fight and keep them in as well, pressure on time.

I: Absolutely. I just have one last question that's just come to me. I'm just wondering about the transition phases that occur in a child's life and the referral processes that you encounter. Is that potentially a barrier from a paediatric perspective?

R: In terms of their ability to access services?

I: To access services, to follow up with them - more so as the child is transitioning - as brain injury is a lifelong injury, how they transition through the paediatric sector, then into the adult sector. Or whether as they transition across sort of life stages, so graduating from school or going from primary school to high school sort of thing.

R: Yeah and I mean I think it's something in general, certainly for OT, we would often say that those key transitions are the times when it might be useful to have OT involved or it might be the time that kind of things pop up that had been flying under the radar. Because, yeah there's changes and you need to adapt to a new situation which is sometimes really tricky for children with a brain injury. So I think, yeah, they are definitely key times that it's worth flagging kids. Like in our setting we don’t really have that ongoing follow up so I guess it's hard for us to really do much about it and I think it is a bit of a hard kind of thing that a lot of the community services also go around those transition times.

So they might see kids up to five years when they're transitioning to school and then they need to start with a new service. Not so much, I guess, throughout the rest but definitely then their transition to adulthood is the same in terms of they have to negotiate a whole new service at that kind of critical time. So I think that's unfortunate and I think it does make an added challenge.

I: Poor little things, it sounds like it's kind of eligibility criteria that get in the way sometimes of access.

R: Yeah well - yeah definitely and I think in terms of that, providing ongoing care for kids with brain injury they do fall through the gap sometimes, especially those really high physically functioning kids who have significant cognitive impairments. It can be really hard to get them the support that they need at school and probably into adulthood as well. They're often not necessarily a priority. I think it's that thing sometimes about brain injury, for those kids not being identifiable, not being physically identifiable by strangers or by the general community so it's really hard to understand their behaviour and identify their needs and prioritise them.

I: Absolutely. I can definitely understand that. It's kind of like the injury is sort of invisible so they're not identified.

R: Yeah.

I: That's all the questions that I have for you. Is there anything that you would like to add that we haven’t talked about today?

R: I don’t think so I mean there's lots to talk about but I think that's all good.

**Transcription ID18:**

I: Thank you for agreeing to participate in the study examining brain injury rehabilitation. I understand you have read the participant information sheet and have consented to complete a short interview. To remind you, our conversation will be tape recorded however, any information you provide will remain confidential and will not be disclosed to anyone other than as part of a summary report, which means your results will be aggregated and your answers will remain non-identifiable. Your participation is also voluntary. You are free to withdraw from the project at any stage. Do you have any questions?

R: No.

I: Okay, so firstly, can you please tell me what are the guiding principles of brain injury rehabilitation within your practice, as a neuro-psychologist?

R: Yes. So from a neuropsych perspective, when I am seeing someone - kids who’ve had a brain injury, I’m thinking first of all about the most likely sequelae given their injury severity and size. What I might expect to see in terms of changes post-injury and my experience from brain injury in terms of providing education and information to the family and the child about that. Then certainly balancing that with the stage of recovery that they’re at but also what they’re presenting or observing as concerns. So, making sure that I’m tailoring any of my rehab interventions towards - yeah, balancing observed concerns with what I know or might expect to see from my experience and knowledge.

I: It sounds like there may sometimes be a little bit of discrepancy between what you see with your trained eyes and what the child and their family may sometimes mention?

R: Absolutely. That can depend on how well the family is adjusting. It can be influenced by their grief reaction or denial around some of the changes that we might see particularly early on in terms of their adjusting or just wanting to get out of hospital for example.

I: Sure.

R: Or like you said, the trained eye - that I might knowing about what might - so sometimes educating them about what they’re actually seeing or experiencing and then putting that into context of the brain injury itself for them.

I: Sure. In your opinion are brain injury rehabilitation principles different to more general rehabilitation principles?

R: Yeah, definitely. So from - I guess from my perspective general rehab, my understanding of general rehab principles are to help the patient return to their usual function after an accident.

Brain injury - we don’t really know what we’re aiming for because brain injury’s long term and often does impact; so I guess different to a knee injury for example where we might be working on returning to full function of the knee. A frontal lobe brain injury for example, we don’t know, especially in kids, what the ongoing impact is going to be of that injury. So looking at that it’s a bit of a crystal ball question in what we’re aiming for in terms of the recovery.

I: It sounds like it might be a little bit different for paediatrics in particular, due to developmental stages, is that correct?

R: Definitely. So I guess with some paediatricians, particularly like you said, that the injury happens to a developing brain and so any skills that may not have been acquired yet, the future development of those skills may be affected by the injury. So in terms of minimizing the impact of that injury damage, we don’t know what we’re aiming for in terms of the development of those skills in the future.

I: Sure. The next few questions ask about your thoughts on what is whole of person practice in paediatric brain injury rehab. From your experience, how would you define holistic practice in brain injury rehabilitation?

R: So - can you repeat the question for me, [Bonnie]?

I: Yeah, no worries. How would you define holistic practice in paediatric brain injury rehabilitation?

R: So for me, holistic practice refers to seeing the whole child rather than just the aspect of the child that my discipline specifically might be interested in. So from a neuropsych perspective I’m, of course, interested in cognition, memory, behaviour, emotions but I need to see that holistically in the context of the child who also does other things likes walks and talks and other skills that might be affected by the injury.

Then also see that child, in context of who they are in terms of their identity, their likes and their dislikes; what they spend their time doing other than being a student at school but then in terms of their roles as a student; as a peer; as a sibling; as a daughter or son and how they fit in their context and see the child in their context, as well, I think is true holistic rehab.

I: Sounds like a big job that you have?

R: Yes.

I: [Laughs] Some people might find it challenging to define whole of person practice; I’m just wondering your thoughts as to why this might be?

R: I think like you said it’s challenging. I think that trying to put all those pieces together can be quite overwhelming; it’s quite complex. I think working through all of those different layers, I guess might be - unavoided is the wrong word but it's simpler to look at things more specifically rather than holistically, I think. I also think there are differences in terms of our discipline's training as to what holistic care or rehab might involve and it’s very easy to see, I guess a more narrow focus to work on.

I: In terms of perhaps university education and training do you mean?

R: Yes. Yes that’s what I meant. So, each discipline has different levels of training but also different focus of training in terms of what you specialize in. We don’t necessarily - we learn about our specific areas and the aspects of functioning post-injury that we might be interested in. I don’t know whether we do learn so specifically about how to think about that in the context of the big picture for the child.

I: Yes that’s a very good point because just thinking about university experiences, I guess disciplines don’t often train working together at uni; it’s not until you, I suppose, get out into the profession where you actually start working together with other practitioners from different disciplines?

R: Absolutely.

I: Very interesting. What do you think would be the opposite of whole of person practice?

R: I think looking just at your specific area. So for me, if I was just considering memory without thinking or looking at memory strategies for example without thinking about the impact of that on other areas of rehab, or other areas of function, or if I was just looking at - if I was a physio and I was looking at just knee rehab or knee function and wanting to suggest strategies or an exercise program to improve knee function without thinking about the fact that the child may have memory difficulties or thinking that they’re in a family context, that doesn’t really have the resources to follow through on that program. I think that would be an example.

I: How exactly might whole of person practice change across practitioners and settings? So for example the acute, the sub-acute and community - your answer can be as broad or as specific as you like.

R: Yeah. I think it does change in terms of in the more acute phase. The approach needs to be more specific or medically orientated with specific goals that are more driven by the medical staff and the therapists, and I think that changes over time in terms of how as the child moves from the hospital to the home and their other contexts in the community, school, what have you, the goals change as the families and the children involved become more able to participate in devising rehab goals and driving what’s actually happening.

I: It sounds as though family appears to be an important theme for paediatric brain injury rehabilitation. I’m just wondering how practitioners actively involve families in their conceptualization or approach to rehabilitation?

R: So personally, all of the work that I do involves the family so the family would be my starting point. I wouldn’t work with the child before I’d spoken with the family to find out where they’re at in terms of their adjustment and grief and coping with what’s happened to the child, but also their understanding of what’s happening and what their concerns are. Then we’d work on goals to address whatever those concerns might be together with the family and the child and also the rest of the team, the rehab team.

I: What specific interventions, I guess, or how do you go about involving families?

R: Ah yes, so when they’re here on the ward, I might meet with them for interview. Interview them either by myself or together with someone else on the team such as social work or I might gain their permission to come along to an OT session, a therapy session to observe and then interact with the families about how that’s gone. If I don’t catch them while they’re here on the ward, then I would do that by phone but it would always be either a face to face or phone interview to introduce myself and introduce my role in the rehab process to recruit, I guess a shared goal, some shared goal setting.

I: Sure, sounds like a very collaborative sort of approach.

R: Try to make it that way.

I: Yeah. [Laughs] So we’ve touched on this a little bit in terms of family, but in a more sort of broader, more general scope of discipline and setting, how do you achieve whole of person practice within [organisation name withheld]?

R: How do I achieve that? I’m just trying to think back to those questions. If you’re asking me in terms of my role as neuro-psychologist, how I approach it myself?

I: Yes and then as well as within the setting. So within a multi-disciplinary team.

R: Yeah. Sorry, I’ve gone completely blank, [name withheld].

I: It’s okay.

R: In terms of - I think, when I’m working with the families and the kids, I’ll always, I guess spend a bit of time myself trying to think through what my understanding or expectations might be of what I might see from the kids and the families and find all the information out that I need to. Then talk with the team about what’s happening from other perspectives in terms of what progress has already been made; what concerns are coming up; tips on how to work with the family. Then talk with the family about what their concerns and their goals are and try and match those together and balance what I know and the information I have in my head, with working together and moving them forward in the rehab process from a neuropsych perspective. Does that answer the question?

I: Yes, thank you. What external factors do you think might influence the real world delivery of whole of person practice?

R: Yeah, so external factors might be things like the willingness and motivation of the families to be involved in other aspects of care. I think some families potentially might just want a physio just to look at the knee rather than get into the whole of person type approach. I think sometimes, because as we said before, it’s quite complex. I think time can get in the way for practitioners. So it’s easier just to stick to your stuff. I think that sometimes the training and experience that we’ve had might prevent that from happening as well.

I: How about working within a multi-disciplinary team, does that cause frustrations at times?

R: Absolutely and I think it can kind of, in terms of providing holistic care, I think it can go either way. So sometimes, certain disciplines might be more readily able to stick to their own, I guess, specific areas or specific functions of interest and not be amenable to be thinking about the impact of others. Whereas on the other side of that, sometimes I think being exposed to that multi-disciplinary environment, where you get to hear about different ways and different perspectives that people are thinking about the same child, invites and encourages that more holistic thinking about things.

I: Sure. My final question is just regarding two terms, sort of more of a statement I guess; holistic practice and family- centred practice – how would you define those terms or perhaps are they more inter-related in a way?

R: I think they go hand in hand. I don’t think that you can treat a child holistically in terms of rehab without considering them in their family context. I mean, I guess it gets hard to define because your family-centred care is not necessarily the same as seeing a child in context either. You might be aware of the context but not necessarily modify your practice accordingly.

Personally, I don’t think you can see the whole child without seeing who they are and their identity and their roles within the family and how, I guess, client-centred care then for me and that whizz kids goes hand in hand with family-centred care in terms of understanding where the family’s at and what their goals are and aligning rehab goals.

I think often, therapists have their own goals and then go for those goals, rather than aligning that with where the family and child are at.

I: Sure; that’s wonderful. Is there anything you would like to add that we haven’t spoken about today?

R: No, that’s it.

I: Thank you.

**Transcription ID19:**

I: Thank you for agreeing to participate in this study examining brain injury rehabilitation. I understand you have read the participant information sheet and have consented to complete a short interview. To remind you, our conversation will be tape recorded. However, any information you provide will remain confidential and will not be disclosed to anyone other than as part of a summary report, which means results will be aggregated and your answers will remain non‑identifiable. Your participation is also voluntary and you are free to withdraw from the project at any stage. Do you have any questions?

R: No.

I: Okay. So firstly, just regarding brain injury principles, can you tell me what are the guiding principles of brain injury rehabilitation within your practice as a speech pathologist?

R: So I guess for me the main things are looking at developmental principles, so that’s really important in considering a child post‑acquired brain injury. Family‑centredness principles I think are really important, and then also what I know about communication and acquired brain injury in the developing brain, so that’s really - those principles need to guide my input and what my framework is.

I: Sure. Are brain injury rehabilitation principles different to general rehabilitation principles in
 your opinion?

R: I think they probably are. I think there's probably different factors that you need to be aware of in terms of the brain injury side of things, but I think overall the principles potentially are - there is some crossover.

I: In what way, if you could just elaborate just a bit more?

R: Yeah, sure. So I think - and it probably is more about the strategy that you use, so I think that there’s certainly, definitely, similarities between, say, a brain injury where you have perspective versus, say, a child that’s having developmental difficulties in certain areas of language, so I think it’s probably more about the strategies that are similar, that can be similar, but the reasons for needing them are different.

I: Sure. The next few questions ask about your thoughts on what is whole‑of‑person practice in paediatric brain injury rehab. From your experience, how would you define holistic practice?

R: So for me holistic practice is looking at the child and the family and where they’re at in terms of what they’re wanting to achieve for the child and where they’re at in terms of coping with their grief, loss and adjustment. So holistic practice for me is understanding that what I’m seeing clinically and observing from a communication or a swallowing point of view may not actually be what the family is prioritising at that point in time, and having that - I guess working with the family with that, so not me saying to them, well, these are the things you need to be worried **a**bout. Me having awareness of what I’m looking at, giving the family enough information, but also sitting with the fact that that might not be a priority for them at that point in time.

I: Sure. It sounds like it’s a very collaborative effort with the family.

R: Yeah.

I: Cool. Some people might find it challenging to define whole-of-person practice. I’m just wondering your thoughts on why that might be.

R: On why it’s hard to define?

I: Yeah, why some people might find it a bit difficult to define whole-of-person practice.

R: Yeah. I think - well, I think whether they find it difficult to define, or whether they find it difficult to put actually in practice, I think sometimes as clinicians we get caught up in what we perceive as being important and we do lose the fact that maybe that isn’t a priority at that point in time. So I think it’s balancing what you’re clinically responsible for and what you’re clinically seeing with whether or not that’s a priority at that point in time.

I: Sure. What do you think would be the opposite of whole-of-person practice?

R: I think probably more compartmentalising, so me seeing a child purely as their language issue or their speech difficulty rather than putting it into, well, actually, the reason why you need to speak clearly is so that you can talk to your friends and family, so becoming more - very specific, I guess, and impairment‑focused.

I: Yes. How exactly might whole-of-person practice change across practitioners and settings, so for example, the acute, sub‑acute and community trajectory?

R: I think probably in the acute phase there is more of a safety of the patient and there’s perhaps some less flexibility in targets. So it probably is a bit more impairment‑based at acute phase, and then it becomes less impairment‑based and more functionally‑based, I feel.

I: Yeah. So how the patient is able to interact with their environment?

R: Yeah. Yeah.

I: Sure. The next few questions are about how you might achieve whole-of-person practice in your discipline and setting. Firstly, how do you achieve whole-of-person practice in your discipline as a speech pathologist?

R: I always consider where the child is at in terms of their life stage and what they need to be doing in that phase, so are they - is the focus on getting them back to school, so if that’s the case, I need to make sure that therapy requirements are supportive of that process, rather than hindering to that process.

I: How do you achieve whole-of-person practice in your setting within a multi‑disciplinary team?

R: I think having team consultation, team discussion, on a regular basis is really important, but also really making sure that you tap into the resources that you know - you actually do understand where the family are at and where - what they see as a priority.

I: Sure. I’ve just thought of this one as well. How do you see other practitioners implementing whole-of-person practice?

R: I think it’s very variable. I think when people can move away from just them being the expert and that that’s really - I see when people perceive their role as they’re the expert, that impacts on whole-of-person practice, so when you’re seeing the family as the experts in their child, that to me is more whole-of-person practice.

I: Yeah. What external factors do you think might influence the real world delivery of whole-of-person practice?

R: I think the clinical safety aspects. There’s going to be an element of, as a clinician, you may not feel comfortable, like I’m just thinking, for example, with a boy we have at the moment, his swallowing is significantly impaired because of his brain stem injury. Now, for me as a speech pathologist, I need to make sure I’m offering him a safe option in terms of his swallowing so that I’m not going to cause any harm to him in terms of infections or aspiration or choking. But that might actually be quite different in terms of what he wants to eat or drink himself - a 16 year old boy, so I think that clinical safety can sometimes impact.

But I think also a big thing is people’s personality, in terms of whole‑person practice, because I think there’s a level of you have to have some self‑awareness in terms of why you’re pushing for something if the people aren’t on board with that. I think everyone has a different level of feeling comfortable with, hey, this is my job and I have to do it, with also, well, actually, the family aren’t perceiving that as an issue or a need, so maybe you need to step back. So yeah, I’m not sure if I’ve explained that very well, but I think people’s personalities and I guess their need to control situations can impact on that.

I: Yes. It sounds like the family‑centred practice is very much a driving force in the way practitioners approach whole-of-person practice.

R: Yeah.

I: I’m just wondering, in your view, whether there’s a difference between defining holistic practice and defining family‑centred practice.

R: Possibly. I think, you know, if you look at whole of person, well, we’re looking at the child and we’re looking at what we’re perceiving as the priorities for that child, considering their age or - but you kind of can’t just look at the child. You’ve got to look at them in the context of their family and their environment and what is going to be realistic to achieve and what…

I: Yes.

R: Yeah. So I think they probably are quite different, but certainly impact on each other. I think the family‑centredness impacts on whole of person.

I: Absolutely. You mentioned earlier as well the developmental stage of a child. The paediatric literature has suggested that for children with a brain injury time elements can be an implication because of the developmental stages, and I guess sometimes the injury can remain invisible until they reach these developmental stages. I’m just wondering, in the sometimes short amount of time that you spend with a client or a patient, how that can impact on your practice.

R: In terms of that longer term. Well, I think you have to - the literature does say - indicate that there are those longer term issues, but I think it also has to come back down to the mechanism of injury, and certainly there is quite a difference between, say, traumatic injury and age of injury and, say, acquired, so more likely a bleed. So I think the mechanism of injury is really important to consider, and then, I guess, in making sure that in your management that you’ve given the family enough education to - and tapped in the resources that are needed for the longer term, and giving them the transition times where you potentially would find some difficulties and then what the options would be in terms of support for that.

So, yeah I think you’re right, it’s a very short window of input, but it’s about making sure the family are educated with the longer term plan, but also not taking away - like we can’t predict, so, yes, we know that the children are at higher risk, say, for their high‑level language development once they hit 16. But we also don’t know - there’s so many other influences that can impact. It can’t all just be about - the brain injury is part of it, but I guess there’s other - lots of other things that can happen that can influence as well.

I: Yes. The important transition phases that you’ve mentioned as well, are they sometimes problematic in terms of attempting to provide whole-of-person practice?

R: I’m not sure, so - potentially. I mean, if you’re saying to a family, well, look, prior to entry to school, you might find some issues around whatever it is communication‑wise, and if the family aren’t wanting to necessarily - that’s not a priority for them, well, then, I guess, yeah, it would, because there’s a need there that you’re seeing but it’s ultimately then - if you’re back to - sorry, I’m talking family‑centred rather than whole person. Yeah, I think it can be an impact.

I: My final question is family appears to be an important theme for paediatric brain injury rehab. I’m just wondering how exactly practitioners actively involve families in their conceptualisation or approach to rehabilitation?

R: Well, they’re certainly part of sessions. You’re gathering a lot of information in terms of pre‑morbid difficulties, which can be quite overwhelming for families, which you need to be, obviously, sensitive about. But it’s - you really need to have lots of conversations and consultations with the family and on explanations as to: what you’re looking at, what they’re seeing, how you can problem solve together.

I: Yes. And I guess the education element that you’ve mentioned becomes a major part of that, so that it is collaborative?

R: That’s right, and also through the assessment process that we do post‑injury. That’s a good way of feeding back to families. I would say the majority of families are very good at knowing what - they see the areas that they’re wanting to focus on. So I mean I think most of the families do have an awareness of what’s going on for their child.

I: What do you do as a practitioner when there’s an element of resistance on the family’s behalf? Because obviously they will be in shock and trying to come to terms with what’s happened to their loved ones and their children, so I guess I wonder what happens when you come up against resistance?

R: Well, I think you do have to be self‑aware, that, you know, there has been plenty of patients over my time that you feel like you’ve had a good rapport, and it’s usually the area that is not progressing in the way the family - as quickly, and sometimes communication can be one of those areas.

I think having some self‑awareness as to what the grief process is for the family and then how that may or may not impact on you, and making sure you seek out the support that you need as a clinician so that you’re not then confusing things with your, I guess, feelings about where things are at. I think you need to work closely with the psychosocial team on the team. I think just feeling - making sure that you do respect where the family is at, and maybe they don’t want you to assess their child, and maybe that’s actually fine, even if there is a communication issue.

I: Yes, absolutely. Thank you so much…

R: Thanks okay, [name withheld].

I: …for doing the interview. Is there anything else that you would like to add that we haven’t spoken about?

R: No.

I: No, that’s fine. Okay, I’ll turn the recording off.
